# Negative frequency-dependent selection and asymmetrical transformation stabilise multi-strain bacterial population structures

**DOI:** 10.1038/s41396-020-00867-w

**Published:** 2021-01-06

**Authors:** Gabrielle L. Harrow, John A. Lees, William P. Hanage, Marc Lipsitch, Jukka Corander, Caroline Colijn, Nicholas J. Croucher

**Affiliations:** 1 MRC Centre for Global Infectious Disease Analysis, Department of Infectious Disease Epidemiology, Imperial College London, Norfolk Place, London, W2 1PG UK; 2 Center for Communicable Disease Dynamics, Department of Epidemiology, Harvard T.H. Chan School of Public Health, 677 Huntington Avenue, Boston, MA 02115 USA; 3 Department of Biostatistics, University of Oslo, Oslo, Norway; 4 Helsinki Institute of Information Technology, Department of Mathematics and Statistics, University of Helsinki, Helsinki, Finland; 5 Parasites & Microbes Programme, Wellcome Sanger Institute, Wellcome Genome Campus, Hinxton, Cambridge, UK; 6 Department of Mathematics, Simon Fraser University, Burnaby, BC Canada

**Keywords:** Population genetics, Microbial ecology, Microbial genetics, Bacterial genetics, Phylogenetics

## Abstract

*Streptococcus pneumoniae* can be divided into many strains, each a distinct set of isolates sharing similar core and accessory genomes, which co-circulate within the same hosts. Previous analyses suggested the short-term vaccine-associated dynamics of *S. pneumoniae* strains may be mediated through multi-locus negative frequency-dependent selection (NFDS), which maintains accessory loci at equilibrium frequencies. Long-term simulations demonstrated NFDS stabilised clonally-evolving multi-strain populations through preventing the loss of variation through drift, based on polymorphism frequencies, pairwise genetic distances and phylogenies. However, allowing symmetrical recombination between isolates evolving under multi-locus NFDS generated unstructured populations of diverse genotypes. Replication of the observed data improved when multi-locus NFDS was combined with recombination that was instead asymmetrical, favouring deletion of accessory loci over insertion. This combination separated populations into strains through outbreeding depression, resulting from recombinants with reduced accessory genomes having lower fitness than their parental genotypes. Although simplistic modelling of recombination likely limited these simulations’ ability to maintain some properties of genomic data as accurately as those lacking recombination, the combination of asymmetrical recombination and multi-locus NFDS could restore multi-strain population structures from randomised initial populations. As many bacteria inhibit insertions into their chromosomes, this combination may commonly underlie the co-existence of strains within a niche.

## Introduction

One of the earliest descriptions of “strains” of an infectious disease referred to “strains of lymph”, pus containing cowpox or vaccinia viruses, that were propagated between individuals in smallpox vaccination programmes [1]. In the early 20th century, the term was applied to the bacterium *Streptococcus pneumoniae* (the pneumococcus) [2], an obligate commensal of the human nasopharynx, variants of which could be differentiated by their ability to cause disease in a mouse model [3]. Similar variation in strains’ propensity to cause invasive diseases is observed in humans [4, 5]. This underlies the effectiveness of the pneumococcal polysaccharide conjugate vaccines, which do not change nasopharyngeal carriage rates, but instead alter the bacterial population composition to reduce overall invasiveness [6]. Hence understanding pneumococcal strain dynamics is critical when designing interventions to reduce disease [5].

Like many other bacteria, *S. pneumoniae* was originally shown to cluster into genetically similar groups through electrophoretic analysis of polymorphic enzymes or restriction fragments [7–9]. Multi-locus sequence typing confirmed such clusters persisted despite ongoing exchange through recombination [10, 11]. Population genomics has shown isolates within these clusters are similar in both core genome sequences and genome content [12, 13]. The consistency of typing assignations by different methods suggests strains are meaningful biological entities [13, 14]. Yet, mirroring the broader problem of understanding bacterial speciation, co-existence of diverse genotypes in multi-strain populations (MSPs) has proved challenging to reproduce through evolutionary models [15–17].

Neutral models have sought to account for the persistence of MSPs in the absence of selected phenotypes. MSPs could be mixtures of ephemeral genotypes [18], with strains representing multiple sampling of local transmission chains, or microepidemics, within a diverse population [19]. This could account for the divergent strain compositions of cross-sectional samples from different countries [4, 19, 20]. Yet, longitudinal sampling of individual populations [21, 22] and phylodynamic reconstructions of strains’ evolutionary histories [4, 23, 24] concur that strains persist over decades. An alternative neutral explanation for high intraspecific diversity is allopatric diversification through “isolation by distance” [25, 26]. However, this is unlikely to explain the diversity of *S. pneumoniae* strains, given their intercontinental distribution [4, 23, 24], and the similar frequencies of polymorphic loci in distant locations with different strain compositions [20]. This mixing is consistent with rapid migration between locations, which can facilitate population diversification if combined with repeated dissemination and local species-wide extinctions [27]. Yet, *S. pneumoniae* is stably endemic worldwide [4, 22].

Alternatively, transient separation of bacterial lineages may allow sufficient diversity to accumulate to inhibit homologous recombination, preventing subsequent convergence if they re-encounter one another [15, 28]. However, neutral simulations predict even different streptococcal species would eventually merge through homologous recombination [15, 29], as exemplified by previously distinct *Campylobacter* species [30]. Correspondingly, interspecies recombinations between streptococci are readily detectable [31, 32]. At the intraspecific level, there appear to be few mechanistic limitations to exchange of core loci between *S. pneumoniae* strains [33], with no evidence of increased frequencies of recombination between closely related genotypes [34], and variable loci frequently diversifying through transformation [23, 24, 35].

Divergence may instead reflect bacterial ecology. In the ecotype model [36], two processes preserve strains’ distinctive genotypes. The first is “isolation by adaption” [37], with exchange between strains limited by their confinement to distinct [38], or even overlapping [39], niches. Yet, with the possible exception of an atypical lineage [12, 39], *S. pneumoniae* strains co-circulate in the same populations, and frequently directly compete with one another within individual hosts [40, 41]. This implies their niches are insufficiently separate to prevent sequence exchange. The second process is selection against acquisition of locally adaptive [42] or niche-specifying [43] loci. These are maladaptive in the recipient’s niche, but are individually insufficient to enable the recombinant to outcompete the donor genotype in its niche, assuming ecotypes’ adaptation to involve multiple loci [44, 45]. This preserves MSPs by selecting against sequence exchange between different strains, a situation termed “outbreeding depression” [46], as recombinants’ fitness is typically reduced relative to the parental genotypes. However, *S. pneumoniae* strains have little private gene content [12, 20, 47], suggesting there are few stable niche-specifying loci. Such a pattern could indicate strains are not irrevocably adapted to particular ecologies, with transiently associated mobile loci instead facilitating “recurrent invasion” [38] of different niches. However, while conjugative elements often carry phenotypically important “cargo” in *S. pneumoniae*, they are stably associated with strains [12, 24, 48]. Prophages are more variable across the species [12, 13, 24, 48], but these elements have not been observed to commonly carry cargo genes [12, 49], as in some other bacterial species. Hence, there is limited scope for frequent specialisation of *S. pneumoniae* to “nano-niches” [43].

Alternatively, strains may be adapted to different immunological niches, diversity in which results from variation in immune responses across a host population [50–52]. Variable antigens are likely to be under negative frequency-dependent selection (NFDS), which results from a phenotype conferring its greatest benefit to an individual when it is rare in the population [53], as epitopes are more frequently recognised by adaptive immunity when they are common [54, 55]. If multiple strongly immunogenic antigens are simultaneously under NFDS, then strains are predicted to emerge from a freely recombining population through selection of discordant antigen combinations that minimise cross-strain immunity [56]. MSPs are thereby maintained by outbreeding depression resulting from recombinants being recognised by cross-immunity induced by either parental strain [57]. However, the distribution of antigen variants is not strongly discordant across *S. pneumoniae* populations [58], which may reflect immunity induced by *S. pneumoniae* colonisation only weakly protecting against reinfection by the same antigen profile [59, 60], and the immunity induced by exposure to one strain recognising much of the species-wide diversity of even variable antigens [61]. This model also predicts the structuring of MSPs by immunity should change with hosts’ contact network [62], which is not apparent from comparisons of Western *S. pneumoniae* populations with those from a more isolated refugee camp [20]. Strong immune selection is also predicted to drive similar levels of diversity at each antigenic locus [63], whereas *S. pneumoniae* antigens exhibit very different levels of variation within and between strains [22–24, 58].

Discordant antigen models have been extended to incorporate virulence [21] and ecotype-style metabolic adaptation to niches through particular sets of core gene alleles [64]. More generally, strains may emerge as combinations of co-evolving loci [46]. The disruption of advantageous epistatic interactions contributing to each strain’s fitness can cause sufficient outbreeding depression to maintain MSPs [46]. However, the polymorphic loci characterising *S. pneumoniae* strains are individually found in many combinations across the species [12, 47], and genome-wide epistasis analyses suggest co-evolutionary associations are focussed on a few key loci [65, 66]. Hence, none of the models of MSPs conceived prior to routine population genomics studies can be easily reconciled with such datasets, and there is an opportunity to use this information to improve our understanding of bacterial evolution [27, 67].

One model developed using multiple collections of genomic data assumed all intermediate-frequency accessory loci (i.e. those present at between 5 and 95% prevalence in the population) were maintained at “equilibrium frequencies” by NFDS acting on multiple phenotypes (the multi-locus NFDS model [20]). This framework was able to replicate the short-term post-vaccine dynamics of *S. pneumoniae* MSPs [20, 68]. The multi-locus NFDS model differs from its antecedents in that genotypes compete within a homogeneous niche across multiple loci, each of which independently contributes to a genotype’s overall fitness. Yet, the model did not feature recombination, and therefore whether it was consistent with the emergence and persistence of MSPs was unclear.

The majority of pneumococcal intermediate-frequency accessory loci are not autonomously mobile [20], and therefore their transfer primarily occurs through homologous recombination, which is frequent in the naturally transformable pneumococcus [69]. While core loci are typically modelled as being symmetrically exchanged (i.e. allele transfer rates are independent of donor and recipient genotypes) [29, 70], transformation with accessory loci is asymmetric [71]. This is because any multi-gene accessory locus can be deleted, through recombination between a recipient encoding the locus and a donor lacking it, substantially more efficiently than it can be inserted through the reciprocal recombination [72]. Hence, the multi-locus NFDS model was modified to incorporate different modes of recombination affecting the core and accessory genomes, and simulations were run to test whether MSPs could be maintained over longer timescales.

## Materials and methods

### Structure of the model

The previously described multi-locus NFDS model [20] was constructed using a Wright–Fisher framework. At time *t*, each isolate *i* contributed a Poisson-distributed number of progeny, *X*_*i,t*_, to the next generation. The total number of isolates in the population at *t*, *N*_*t*_, was constrained by density-dependent selection, parameterised by the carrying capacity, κ. Each isolate encoded *L* biallelic accessory loci, each denoted *l*, as encoded by the binary matrix *g*_*i,l*_. If *g*_*i,l*_ = 1, *l* was present in *i*; if *g*_*i,l*_ = 0, it was absent. NFDS was incorporated through comparing the instantaneous frequency of *l* at time *t*, *f*_*l,t*_, with its equilibrium frequency, *e*_*l*_. Therefore, the fitness of an isolate was dependent on π_*i,t*_, quantifying the extent to which it encoded accessory loci at instantaneous frequencies that were above, or below, their equilibrium frequencies

$$\pi _{i,t} = \mathop {\sum }\limits_{l = 1}^L g_{i,l}(e_l - f_{l,t}).$$

The effect on *X*_*i,t*_ was mediated by the strength of NFDS, as parameterised by σ_*f*_

$$X_{i,t} \sim {\mathrm{Pois}}\left( {\left( {\frac{\kappa }{{N_t}}} \right)\left( {1 + \sigma _f} \right)^{\pi _{i,t}}} \right).$$

The model was modified to enable exchange of loci between isolates through transformation. This was parameterised by three variables:

1. Transformation rate, τ: the per-timestep probability of an isolate being a recipient, *r*, in a DNA exchange through transformation.
2. Proportion of genotype affected by transformation, ϱ: the per-transformation probability of each locus *l* being exchanged by recombination.
3. Transformation asymmetry, ϕ: the per-recombination probability of *l* being acquired if it was present in a donor, *d*, but not in *r*.

The probability of an isolate being a transformation recipient was τ per generation. For each transformation, a single *d* was selected from the extant population at random. Each locus in *g*_*r,l*_ underwent recombination with probability ϱ. If *g*_*r,l*_ = *g*_*d,l*_, *g*_*r,l*_ was unmodified. If *g*_*r,l*_ = 1 and *g*_*d,l*_ = 0, then *l* was deleted (*g*_*r,l*_ set to 0). If *g*_*r,l*_ = 0 and *g*_*d,l*_ = 1, *l* was inserted with probability ϕ, else *g*_*r,l*_ remained unmodified. The *f*_*l,t*_ values were recalculated following transformation, prior to the calculation of *X*_*i,t*_, to enable NFDS to reflect the altered gene frequencies.

A further modification was the incorporation of *S* neutral loci, corresponding to core genome single-nucleotide polymorphisms (SNPs; each denoted *s*), into each isolate’s genotype. These were also biallelic, and encoded in a matrix *c*_*i,s*_. These were not under NFDS, and for each pair of *d* and *r*, each SNP underwent recombination with the same probability, ϱ. However, transformation was always symmetrical (φ = 1) for SNPs.

### Parameterisation of simulations

The input data were the 616 genomes from the Massachusetts *S. pneumoniae* collection [22], as represented by the 1090 intermediate-frequency accessory loci in the pre-vaccination population, and 1090 intermediate-frequency biallelic core genome SNPs (Text S1). For each analysed parameter combination (Text S2), 100 stochastic simulations were run for 60,000 timesteps (corresponding to 5000 years). Each simulation was independently initialised as a random mixture of the sequenced genotypes of size κ through sampling with replacement.

**Fig. 1:**
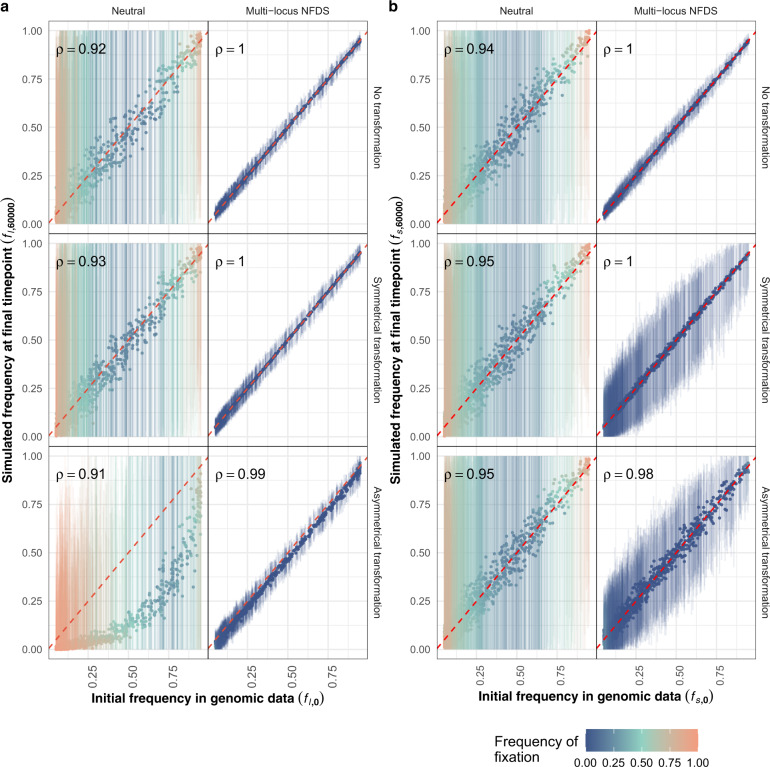
Scatterplots comparing the frequency of alleles at the initial timepoint in the genomic data to their frequency in the final simulation timepoint (*N* = 616 isolates sampled from each simulation). Each panel shows data from a different model parameterisation. Each point (*L* or *S* = 1090 in each panel) represents the mean, and the vertical lines show the range, from 100 replicate simulations. These are coloured by the frequency with which an allele fixed at the corresponding polymorphic locus (i.e. the displayed allele’s final frequency was zero or one in an individual simulation). Spearman’s correlation statistic (ρ) is shown in each panel; all *p* values were <10^−10^. **a** Frequency of accessory loci, in which the alleles correspond to the presence of intermediate-frequency genes. The initial frequencies correspond to the equilibrium frequencies in the model. **b** Allele frequencies at biallelic single nucleotide polymorphism sites. The displayed frequencies are those of the alleles differing from the bases at the corresponding sites in the reference genome, *S. pneumoniae* ATCC 700669.

**Fig. 2:**
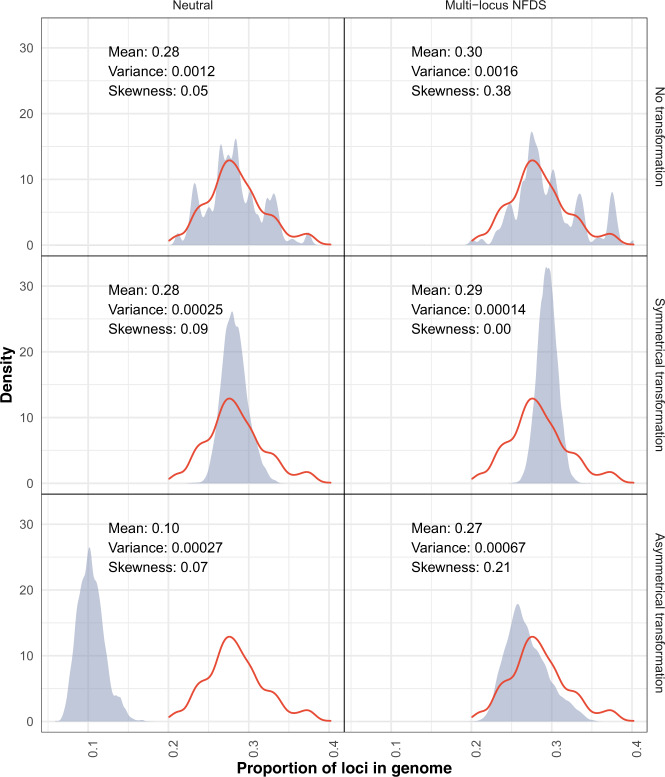
Density plots comparing the variation in gene content in the genome data and at the final timepoint of simulations. Each panel shows data from a different model parameterisation. The horizontal axis represents the proportion of intermediate-frequency loci (*L* = 1090) present in isolates (calculated as Σ^*L*^*g*_*i,l*_*L*^-1^ for each isolate, *i*). The red outline shows the distribution from the 616 genomes in the original dataset (mean: 0.28, variance: 0.00132, skewness: 0.10). In each panel, the blue shading and displayed statistics describe the overall distribution (*N* = 61,600) from 616 isolates sampled from the final timepoint of 100 replicate simulations.

**Fig. 3:**
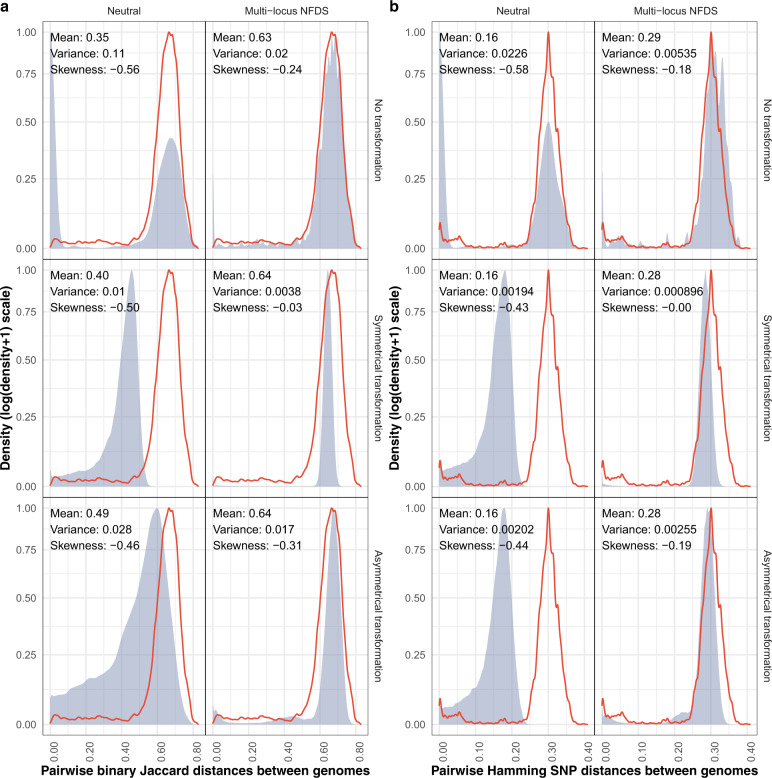
Density plots comparing the distribution of pairwise genetic distances between isolates in the genome data and at the final timepoint of simulations. Each panel shows data from a different model parameterisation. The red outline shows the distribution calculated from the genomic data (*N* = 189,420). The blue shading and displayed statistics in each panel describe the overall distribution of a random 2% sample of the equivalent distances calculated from the final timepoint of 100 replicate simulations (overall *N* ≅ 378,840). **a** Pairwise binary Jaccard distances calculated from isolates’ accessory loci compared with the distribution calculated from the genomic data (mean: 0.63, variance: 0.015, skewness: −0.22). **b** Pairwise Hamming distances calculated from single-nucleotide polymorphisms compared with the distribution calculated from the genomic data (mean: 0.29, variance: 0.0043, skewness: −0.13).

**Fig. 4:**
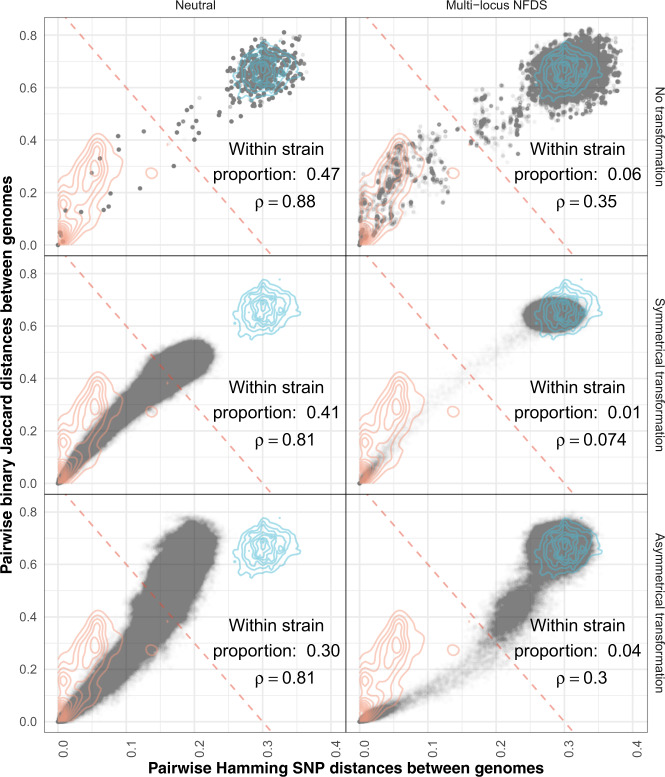
Scatterplots comparing the distributions of pairwise genetic distances between isolates at the final timepoint of simulations. Each panel combines the data from the equivalent panels in Fig. 3 (*N* ≅ 378,840). Each point is a single comparison between isolates, with the horizontal axis representing divergence in core genome single-nucleotide polymorphisms, and the vertical axis representing divergence in accessory loci. The red diagonal is a threshold distinguishing within- and between-strain distances (Fig. S36). The contours describe the distribution of within- and between-strain pairwise distances in the genomic data (orange and blue lines, respectively; *N*  = 189,420). The proportion of pairwise comparisons classified as within-strain, based on the displayed threshold (0.06 in the genomic data), and Spearman’s correlation statistic (ρ; 0.38 in the genomic data) are shown in each panel; all correlation *p* values were <10^−10^.

**Fig. 5:**
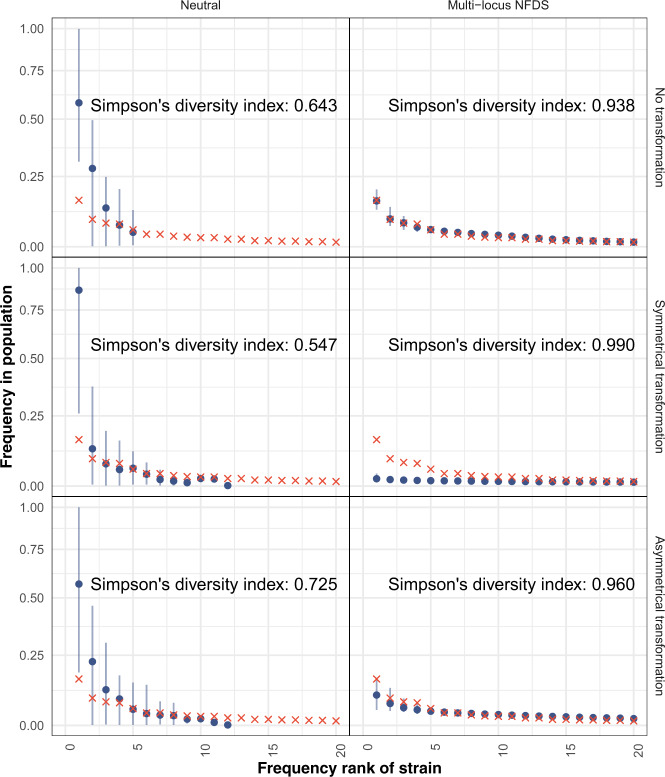
Rank–frequency plots comparing the prevalences of strains in the overall set of 616 genomes (red crosses; Fig. S37), and those from samples of 616 isolates from the final timepoint of 100 replicate simulations. Each panel shows data from a different model parameterisation. The blue points show the mean, and the vertical lines show the range, of strain frequencies for each observed rank across replicate simulations. Each panel shows the Simpson’s diversity index, calculated from the strain frequencies (0.940 in the genomic data).

**Fig. 6:**
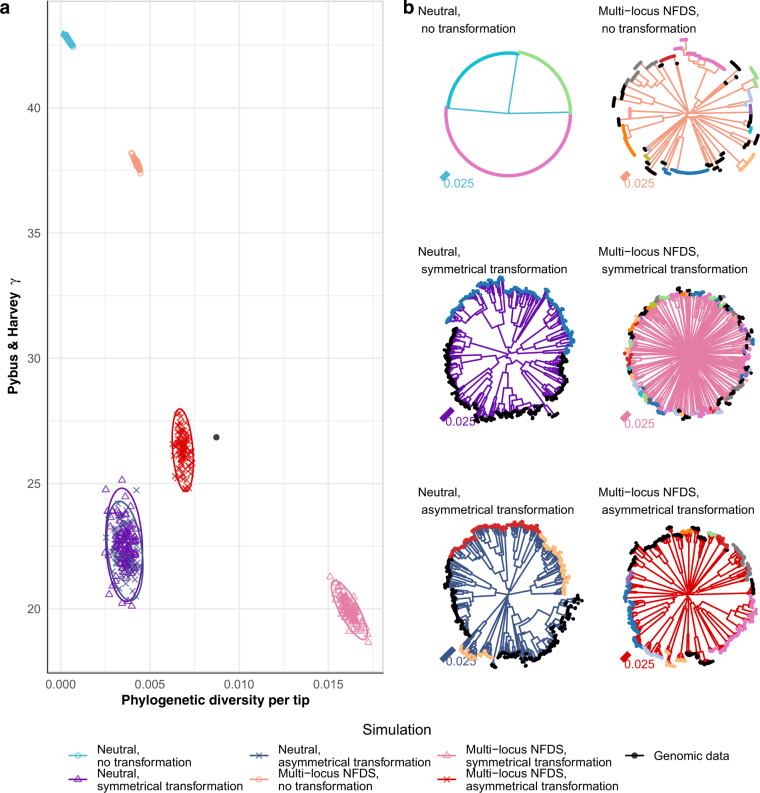
Comparison of trees between the genomic data (Fig. S50) and simulation outputs. **a** Scatterplot comparing the characteristics of the neighbour-joining tree calculated from the core single nucleotide polymorphisms (*S* = 1090) in the genomic data (black point) and those from the final timepoints of 100 replicate simulations for each of six different model parameterisations. The horizontal axis quantifies the variation in the population as phylogenetic diversity (sum of branch lengths) per tip (*N*  = 616). The vertical axis quantifies the branching pattern as Pybus and Harvey’s ɣ. Point shapes and colours represent simulation types. Ellipses describe the distribution of each set of points. **b** Representative trees corresponding to individual simulations from each parameter set. The branch colours indicate the simulation type, and the tip colours correspond to their original strain assignation in the genomic data. Strains that were rare in the genomic data were merged into a single category, which is marked by black tips.

Results presented in Figs. 1–6 represent simulations of a closed population evolving under a specified form of selection (neutral or multi-locus NFDS) and recombination (no transformation, symmetrical transformation or asymmetrical transformation). Some additional simulations modified these model properties:

1. Saltational simulations had altered transformation rates, such that exchanges were less frequent, but more extensive.
2. NFDS strength was reduced.
3. Simulations featuring migration mimicked geographic structuring through allowing inward migration of genotypes from independent simulations.

Other additional simulations modified the starting populations:

1. Permuted input files were generated by shuffling the alleles at each locus across individuals, diversifying the initial population without changing locus frequencies.
2. Randomised input files were generated by selecting the allele at each locus in each individual to be either zero or one with equal probability, such that each locus had an initial frequency of ~0.5.
3. Only a reduced subset of ten of the accessory loci was under multi-locus NFDS.

These differences are summarised in Table 1, and details are described in Text S3.

**Table 1:** Parameter values for each set of simulations.

Simulations	Initial data	NFDS strength, σ	Transformation rate, τ	Proportion of loci affected by transformation, ϱ	Transformation asymmetry, ϕ	Migration rate, *m*
Neutral, no transformation	Genomic; permuted; randomised; reduced subset	0	0	0	1	0
Neutral, symmetrical transformation	Genomic; permuted; randomised; reduced subset	0	0.002625	0.0167	1	0
Neutral, asymmetrical transformation	Genomic; permuted; randomised; reduced subset	0	0.002625	0.0167	0.05	0
Multi-locus NFDS, no transformation	Genomic; permuted; randomised; reduced subset	0.0356	0	0	1	0
Multi-locus NFDS, symmetrical transformation	Genomic; permuted; randomised; reduced subset	0.0356	0.002625	0.0167	1	0
Multi-locus NFDS, asymmetrical transformation	Genomic; permuted; randomised; reduced subset	0.0356	0.002625	0.0167	0.05	0
Weak multi-locus NFDS, no transformation	Genomic	0.0023	0	0	1	0
Weak multi-locus NFDS, symmetrical transformation	Genomic	0.0023	0.002625	0.0167	1	0
Weak multi-locus NFDS, asymmetrical transformation	Genomic	0.0023	0.002625	0.0167	0.05	0
Neutral, symmetrical saltational transformation	Genomic	0	0.000525	0.0835	1	0
Neutral, asymmetrical saltational transformation	Genomic	0	0.000525	0.0835	0.05	0
Multi-locus NFDS, symmetrical saltational transformation	Genomic	0.0356	0.000525	0.0835	1	0
Multi-locus NFDS, asymmetrical saltational transformation	Genomic	0.0356	0.000525	0.0835	0.05	0
Neutral, no transformation, with migration	Genomic	0	0	0	1	0.00001
Neutral, symmetrical transformation, with migration	Genomic	0	0.002625	0.0167	1	0.00001
Neutral, asymmetrical transformation, with migration	Genomic	0	0.002625	0.0167	0.05	0.00001
Multi-locus NFDS, no transformation, with migration	Genomic	0.0356	0	0	1	0.00001
Multi-locus NFDS, symmetrical transformation, with migration	Genomic	0.0356	0.002625	0.0167	1	0.00001
Multi-locus NFDS, asymmetrical transformation, with migration	Genomic	0.0356	0.002625	0.0167	0.05	0.00001

In each simulation, κ = 10^5^.

### Statistical analyses

At the end of each replicate simulation *R*, binary matrices *G*_*i,l,R*_ and *C*_*i,s,R*_ (equivalent to the input *g*_*i,l*_ and *c*_*i,s*_ matrices) were calculated from a randomly selected sample of 616 individuals, to match the input dataset size. The correlation between alleles’ initial frequencies (*f*_*l*,0_ and *f*_*s*,0_ for accessory and SNP loci, respectively) and final frequencies (*f*_*l*,60000_ and *f*_*s*,60000_ for accessory and SNP loci, respectively) was quantified with Spearman’s ρ [73]. Although there are no data against which these values can be directly compared, analysis of divergent populations indicates that allele frequencies should be stable [20].

The proportion of intermediate-frequency accessory loci encoded by each genome was used as a correlate of genome size. As the distribution calculated from *g*_*i,l*_ was asymmetric, it was compared to each *G*_*i,l,R*_ using the non-parametric Kolmogorov–Smirnov statistic [74]. The overall distribution across all replicate simulations for a given parameter set was described using the mean, variance and Kelley’s measure of skewness [75]. The same approaches were used to compare the pairwise distances between individuals, as the negative skew of these distributions in MSPs is fundamental to their division into strains. Hamming distances were calculated from the *c*_*i,s*_ and *C*_*i,s,R*_ matrices, as the assignment of alleles to binary values was arbitrary, whereas Jaccard distances were calculated from the *g*_*i,l*_ and *G*_*i,l,R*_ matrices, to only consider genes present in the pair being compared [12, 70].

As these pairwise distances are proportional to each other in the genomic data, Spearman’s ρ was used to compare their joint distribution across simulations. These data were also described using the proportion of distances classified as within-strain, based on an empirical threshold [13]. The strains defined with this approach were analysed with rank–frequency plots, with the mean frequencies of the ranks observed across replicate simulations summarised using Simpson’s diversity index [76]. The phylogenies generated from the *C*_*i,s,R*_ and the *c*_*i,s*_ matrices were also compared in terms of their branch lengths and topologies. Details of these analyses are described in Text S4. The code used for simulations and statistical analyses is available from https://github.com/nickjcroucher/multilocusNFDS/.

## Results

### Multi-locus NFDS prevents transformation-mediated decay of the accessory genome

One hundred simulations were run for each of six parameter combinations, corresponding to the absence of transformation, symmetrical transformation or asymmetrical transformation, either under neutrality or multi-locus NFDS (see “Materials and methods”). The frequencies of accessory loci at simulation endpoints (*f*_*l*,60000_) were compared to the starting frequencies (*f*_*l,*__0_; Fig. 1a). In neutral simulations in which transformation was absent or symmetrical, *f*_*l,*__0_ and mean *f*_*l*,60000_ correlated significantly. Yet, *f*_*l*,60000_ varied between simulations through drift, with polymorphism often lost at loci with extreme *f*_*l*,0_ values, consistent with initial allele frequency determining probability of fixation [77]. The unfixed polymorphisms likely reflected the simulations having not yet reached equilibrium (Fig. S1).

However, asymmetrical transformation reduced mean *f*_*l*,60000_ and increased the probability of the null, or empty, allele fixing in the population [72]. In such simulations, there was a disproportionate loss of rare accessory loci, resulting from the high prevalence of recombination donors lacking the locus able to cause deletion in recipients possessing the locus. This reduced the overall number of accessory loci per genome (Fig. 2). Hence, under neutrality, asymmetric transformation accelerated the loss of accessory loci.

The loss of polymorphism through both drift and asymmetrical transformation was prevented by multi-locus NFDS, which strengthened the correlation between *f*_*l,*__0_ and mean *f*_*l*,60000_, and reduced the variation in *f*_*l*,60000_ across replicates (Fig. 1a). Correspondingly, the mean number of accessory loci per isolate was maintained close to that observed in the genomic data (Fig. 2). The sequenced genomes’ accessory locus content had a positive skew that was replicated by the simulated populations, except when transformation was symmetrical, which instead homogenised genome characteristics. Excepting those simulations in which isolates’ accessory locus content was preserved by a lack of transformation, the overall distribution of loci between genomes was best reproduced by the combination of multi-locus NFDS and asymmetrical transformation (Fig. S2).

Further simulations tested the robustness of these conclusions to altered starting genotypes or parameters (see Text S3 and Table 1). Initialising simulations with populations in which alleles had been permuted between genotypes had little effect (Figs. S3 and S4). However, using starting populations with randomly generated genotypes resulted in *f*_*l*,60000_ being maintained near their *f*_*l*,0_ of ~0.5 in neutral simulations, unless they were uniformly reduced in frequency by asymmetrical transformation (Figs. S5 and S6). Multi-locus NFDS was only able to shift the *f*_*l*,60000_ close to the locus equilibrium frequencies when the genotypes were reassorted through transformation.

When transformation was saltational, such that exchanges were more extensive but less frequent, few differences were observed from Fig. 1 (Figs. S7 and S8). Neutral simulations featuring inward migration from external populations exhibited less variation in *f*_*l*,60000_ across replicates, as importation of genotypes mitigated some loss of diversity through drift (Figs. S9 and S10). However, reducing the strength of multi-locus NFDS increased the variation in *f*_*l*,60000_ (Figs. S11 and S12), and failed to prevent asymmetrical transformation reducing genome content (Fig. S13). No such effect was observed when undiminished multi-locus NFDS acted on a reduced subset of ten accessory loci (Figs. S14 and S15). Hence, there is a minimal NFDS strength required to prevent loci being lost through asymmetrical transformation.

### Multi-locus NFDS stabilises frequencies of unselected polymorphisms

Neutrally evolving SNPs were also included in the simulations. If transformation occurred, these were exchanged symmetrically. In neutral simulations, the mean final SNP frequencies (*f*_*s*,60000_), and probabilities of fixation, were similar to those of the accessory loci when transformation was absent or symmetrical (Fig. 1b). Multi-locus NFDS indirectly stabilised the SNP frequencies, despite only acting directly on the accessory loci. This effect was greatest in the absence of transformation, such that all accessory and SNP loci maintained the linkage embedded in the original population. Greater variation in *f*_*s*,60000_ was evident between replicate simulations featuring transformation, likely reflecting the weakened linkage with the selected accessory loci.

Permuting the initial genotypes had little effect on *f*_*s*,60000_ (Fig. S3), whereas these frequencies remained close to 0.5 when the starting population was randomly generated (Fig. S5). In simulations featuring saltational recombination, variation in *f*_*s*,60000_ was reduced in simulations combining NFDS and asymmetric transformation (Fig. S7), whereas inward migration reduced *f*_*s*,60000_ variation in neutral simulations (Fig. S9). Weakening NFDS (Fig. S11), or reducing the number of loci on which it acted (Fig. S14), increased the variation in *f*_*s*,60000_ between replicates across all non-neutral simulations. Therefore, the correlation between *f*_*s*,0_ and *f*_*s*,60000_ was higher when multi-locus NFDS was stronger, and when transformation was less frequent.

### Pairwise distance distributions are shaped by selection and recombination

The variation in gene content across the population was described by calculating the pairwise binary Jaccard distances between all genotypes sampled at the simulation endpoints, based on the intermediate-frequency accessory loci they encoded. In the genomic data, there is a single mode of between-strain distances, and a tail of shorter within-strain distances that make the distribution negatively skewed (Fig. 3a). In the neutral simulations, the mean pairwise distances were reduced through two mechanisms. Firstly, the loss of genotypes through drift meant the within-strain peak increased in prominence relative to the between-strain peak, as the population simplified. Secondly, transformation drove convergence between genotypes, such that the range of pairwise distances was decreased [13, 70, 78]. The effect was least pronounced for asymmetrical transformation, as the number of accessory loci per genome (to which the Jaccard distance is inversely proportional) was decreased across the population (Fig. 2).

Multi-locus NFDS maintained the position of the between-strain mode, regardless of transformation. However, the tail of within-strain distances was only maintained when transformation was absent or asymmetrical. Symmetrical transformation instead homogenised the distances into a unimodal distribution with low variance. Consequently, multi-locus NFDS simulations consistently reproduced the observed pairwise distance distribution more accurately than neutral equivalents, and simulations featuring symmetrical transformation were the least accurate (Fig. S16).

The pairwise Hamming distances were also calculated from the SNP alleles encoded by the simulated genotypes (Fig. 3b). In the genomic data, these again had a between-strain peak, and a tail of shorter within-strain distances. Each of these simulation outputs was similar to the corresponding set of accessory genome distances (Fig. S17), with the exception of neutral simulations featuring asymmetrical transformation, in which SNPs were symmetrically exchanged. Hence, all the neutral simulations had a mean pairwise Hamming distance approximately half that calculated from the genomic data. In all multi-locus NFDS simulations, the mean divergence was maintained near its observed value. However, symmetrical transformation again homogenised the pairwise distance distribution, and hence the tail of within-strain small SNP distances was only evident if transformation of accessory loci were absent or asymmetric.

There was little change to the output of neutral simulations when genotypes were either permuted (Figs. S18–20) or randomised (Figs. S21–23). Simulations initiated with such inputs most accurately replicated the negative skew, and overall shape, of the accessory distance distributions when combining asymmetric transformation and NFDS (Figs. S18, S19, S21 and S22), albeit with simulations featuring NFDS and symmetrical transformation performing similarly well when the input genotypes had been randomised (Fig. S21). These simulations also reproduced the negative skew of the SNP distance distribution most consistently, although none of the simulations using these starting datasets accurately emulated the overall observed pairwise SNP distribution (Figs. S20 and S23). Saltational transformation improved the reproduction of the genomic data if simulations combined NFDS with either form of transformation (Figs. S24–26).

Simulations featuring inward migration mitigated the loss of diversity through drift in neutral simulations, increasing the mean pairwise distances between genomes through the import of strains (Figs. S27–29). Yet, when NFDS was weakened (Figs. S30–32), or acted on fewer loci (Figs. S33–35), it was less effective at preventing transformation driving convergence in the core genome. Hence, multi-locus NFDS acting on a large number of loci maintained between-strain variation, while the preservation of within-strain variation was sensitive to transformation and the starting population.

### Multi-locus NFDS and asymmetrical transformation stabilise MSPs

MSPs are characterised by a positively correlated, discontinuous distribution of accessory and core genome pairwise distances [12, 13]. All parameter sets generated positive correlations in pairwise distances, but the discontinuity separating within- and between-strain distances was less consistently preserved (Fig. 4). This was a consequence of reduced diversity in neutral simulations. Without transformation, the plot became sparse, with most comparisons between identical genotypes at the origin of the graph. With transformation, the convergence between genotypes resulted in a continuous distribution of points, which no longer spanned the observed range of genetic distances. By contrast, simulations with multi-locus NFDS maintained the between-strain distances near their observed position. However, when transformation was symmetrical, there was a paucity of within-strain points. Therefore, multi-locus NFDS only generated MSPs when transformation was asymmetric or absent.

A diagonal boundary can be used to define strains through separating within- and between-strain distances in the genomic (Fig. S36) and simulated (Fig. 4) data [13]. A strain rank–frequency plot demonstrated *S. pneumoniae* populations typically consisted of a few common strains, and a tail of many rare genotypes [4] (Fig. 5, Fig. S37). Neutral simulations consistently generated populations containing few dominant strains, as rare genotypes were lost through drift, and transformation drove the merging of previously distinct strains [28, 79]. By contrast, simulations combining multi-locus NFDS with symmetrical transformation output populations in which all strains were rare, consistent with the absence of small pairwise genetic distances. A mixture of common and rare strains was only observed when transformation was asymmetric or absent.

However, when initialising simulations with permuted (Figs. S38 and S39) or randomly generated genotypes (Figs. S40 and S41), NFDS did not produce a discontinuous pairwise distance distribution without transformation. Yet, simulations combining NFDS and asymmetrical transformation remained effective at restoring MSPs, as judged by the joint pairwise distance distribution and strain diversity. Simulations with this parameterisation further improved their replication of the observed MSP structure when transformation was saltational (Figs. S42 and S43). Migration instead improved the fit of neutral simulations, through importing diversity that mitigated the loss of strains through drift (Figs. S44 and S45). Weakening NFDS did not have a substantial effect (Figs. S46 and S47), although reducing the number of loci on which NFDS acted substantially reduced strain diversity in simulations featuring transformation (Figs. S48 and S49).

### Multi-locus NFDS and asymmetrical transformation shape species-wide phylogenies

Bacterial population structure is often analysed using trees constructed from core genome SNPs. As the distribution of these polymorphisms was affected by both transformation and multi-locus NFDS, neighbour-joining trees were inferred from the genomic data (Fig. S50) and simulation outputs (Fig. 6a), and compared using two statistics. The first, the phylogenetic diversity of the tree (the sum of branch lengths) per tip, represented the overall diversity of the population. The second, Pybus and Harvey’s ɣ, summarised tree shapes using the relative positioning of internal nodes [80]. Higher ɣ values correspond to internal branching events being closer to leaf nodes than expected under neutrality. The highest values were generated by the simulations without transformation, in which many clades were flat (Fig. 6b), as in the absence of diversification through recombination, identical genotypes were repeatedly sampled from the population.

The lowest ɣ values were associated with multi-locus NFDS simulations in which transformation was symmetrical. This reflected the consistently high pairwise SNP distances generating star-like trees, with little internal structure. The many long branches meant these trees also had the highest phylogenetic diversity per tip. The trees describing neutrally evolving populations exchanging sequence through transformation had similarly low ɣ values, but exhibited less phylogenetic diversity, reflecting the convergence of genotypes through recombination (Fig. 3). The trees most similar to that representing the genomic data were generated by multi-locus NFDS simulations in which transformation was asymmetrical. These featured discernible clades separated by deep branches, previously regarded as characteristic of NFDS on a limited diversity of genotypes [81, 82].

The tree statistics were sensitive to simulation conditions. When simulations were initiated with permuted (Fig. S51) or randomised (Fig. S52) genotypes, only phylogenies generated from simulations combining NFDS and asymmetrical transformation had properties that matched those of the genomic tree. Saltational transformation resulted in higher ɣ values, particularly for trees generated from multi-locus NFDS simulations featuring asymmetrical transformation, as flat clades of identical genotypes were more common (Fig. S53). Migration had little effect (Fig. S54). Both weakening NFDS (Fig. S55) and reducing the number of loci on which NFDS acted (Fig. S56) lowered the phylogenetic diversity per tip in non-neutral simulations. Hence, changes to processes that only affected the accessory genome nevertheless shaped trees constructed from core genome variation.

## Discussion

Despite the extensive genotypic and phenotypic variation observed across diverse species such as *S. pneumoniae*, genetic analyses have struggled to identify evidence of selection [27]. This reflects both neutral models’ ability to reproduce particular aspects of bacterial populations [19, 70], and some statistics’ insensitivity in assessing model fits [78, 83]. Population genomics datasets enable multiple comparisons between observation and simulation. The results presented in Figs. 1–5 generally found the best-fitting simulations to be those featuring multi-locus NFDS and no transformation. Given the density-dependent selection inherent to the model, and the absence of recombination making each genotype immutable, multi-locus NFDS stably preserved the original strain frequencies (Fig. S57). This was contingent on NFDS being too weak to drive the chaotic or oscillatory dynamics seen in related models of influenza [57, 84], but acting on enough loci with sufficient strength to prevent the loss of genetic diversity (Fig. S48).

However, assuming no transformation requires a strong recombination barrier between strains, which is not consistent with observed exchanges [23, 24, 47, 48] or the sharing of loci between divergent genotypes [12, 20]. In addition, the maintenance of a realistic population structure by these simulations contrasted with their failure to restore MSPs when the properties of the starting population were altered. When randomly generated populations were simulated in the absence of transformation, multi-locus NFDS was unable to stabilise accessory loci at consistent frequencies (Fig. S5) or reproduce the pairwise distance distribution characteristic of MSPs (Fig. S40). Similarly, when genotypes were generated by permutation of alleles, such simulations resulted in diverse populations represented by star phylogenies (Fig. S51). These resembled the outputs of simulations combining NFDS and symmetrical transformation (Fig. 6). Such unstructured populations represent an equilibrium of the multi-locus NFDS model, in which the majority of isolates are separated by pairwise distances similar in magnitude to those separating strains in the genomic data. This corresponds to a higher divergence than expected between random genotypes, given genes present at the specified equilibrium frequencies (Figs. S18, S21 and S58). As competition occurs between bacteria that share accessory loci, this arrangement lowers the conflict between genotypes, through reducing the overlap between their complements of accessory loci. This is consistent with other NFDS models predicting pathogens will exhibit a strong tendency towards continuous diversification [50].

By contrast, the results of simulations combining asymmetrical transformation and multi-locus NFDS always replicated MSP structures. These simulation outputs either matched the observed data similarly well as (Figs. 2–5), or better than (Figs. 4 and 6), the equivalent simulations lacking transformation when the genomic data were used as the starting population. This was despite genotypes being able to diverge from the starting population (Fig. S57). The distinctive properties of MSPs, including the joint distribution of pairwise distances (Fig. S38 and S40) and the phylogeny structures (Fig. S51 and S52), also emerged when the simulations were initialised with randomised or permuted inputs. Examples where these simulations had not converged on the observed data (Fig. S39 and S41) may represent the simulations having not yet reached equilibrium, given the highly perturbed initial population and slow rates of transformation. Hence, these simulations best explain the emergence and maintenance of *S. pneumoniae* MSPs composed of divergent strain sets in different locations [4, 20].

This tendency to produce MSPs likely emerges from asymmetric transformation and multi-locus NFDS interacting to generate outbreeding depression through a mechanism that does not occur with other model parameterisations. The NFDS aspect selects for a diverse population through maintaining all intermediate-frequency accessory loci at their equilibrium frequencies. However, asymmetrical transformation inhibits MSPs losing their structure, as when new genotypes are generated through sequence exchange, the recipient and recombinant are typically distinguished by the deletion of loci present in the recipient but absent in the donor. This reduces the instantaneous frequency of these loci below their equilibrium frequencies, thereby increasing the fitness of the unmodified recipient genotype (which still encodes these loci) relative to the recombinant progeny (from which these loci have been removed). This mechanism explains both the stabilisation of existing MSPs, and the “pruning” of unstructured populations into distinct strains, through continual removal of low-fitness recombinants. Hence, MSPs are an equilibrium of simulations combining multi-locus NFDS and asymmetric transformation that is robust to strong perturbation of the starting population.

Although NFDS and asymmetrical transformation acted on the accessory loci, they also indirectly limited changes in SNP allele frequency both by selecting against genotypes drifting, through multi-locus NFDS, and against transformation, through outbreeding depression eliminating recombinants. These effects were more pronounced when accounting for the heterogeneity in the rate of recombination in “saltational” simulations (Fig. S7). While this change in transformation parameterisation caused little difference when transformation was symmetric, it resulted in a greater inhibition of recombination in simulations combining saltational asymmetric transformation and multi-locus NFDS (Fig. S59). This improved replication of the joint pairwise distances (Fig. S42) and strain rank–frequency distribution (Fig. S43). Hence, the simplistic modelling of transformation may account for those instances where NFDS simulations featuring asymmetric transformation perform less well than those lacking transformation (Figs. 1–5). Increasing the heterogeneity of asymmetric transformation rates is likely to limit the observation of inter-strain recombinations: larger transformations are expected to be strongly counter selected, if they remove many accessory loci, leaving many isolates essentially unmodified if they are affected by small, or no, transformation events. Yet, despite such disadvantages of inter-strain recombination, asymmetric transformation of *S. pneumoniae* can nevertheless be advantageous overall, due to the benefits of “chromosomal curing” through within-strain transformation [71].

There are many limitations to this model, which are discussed in detail in Text S5. The simulated genetics were simplified by not including mutation, epistasis, linkage or the role of mobile elements. Similarly, the ecology of the bacteria was assumed to be homogeneous and unperturbed. Nevertheless, the results were able to replicate many important aspects of bacterial population genomics, which are likely common to multiple species. Whether multi-locus NFDS can be applied to other microbes is unclear, but the functions encoded by the *S. pneumoniae* accessory genome hypothesised to be under NFDS are common across bacteria [20], and the model was consistent with the strain dynamics within an *Escherichia coli* population [85]. Although transformation is not ubiquitous across bacteria [71], the necessity for mechanisms blocking the integration of parasitic mobile elements is widespread [86, 87]. For instance, restriction-modification systems have frequently been proposed to shape bacterial populations through blocking exchange of DNA [28, 29, 88], but recent experimental work has demonstrated they primarily inhibit the integration of novel genes, rather than the exchange of core genome variation [89, 90]. This would impose a similar asymmetry on recombination at accessory loci as identified for transformation, suggesting multi-locus NFDS could still maintain MSPs through selecting against recombinants with reduced accessory genomes [28, 29, 88]. These processes are likely limited to accounting for diversity within species or subspecies, as the simulated genotypes compete within a homogeneous niche, and consequently the model does not account for ecological differentiation at higher taxonomic groupings [38, 39]. Nevertheless, it can be concluded that phenotypic variation between sets of bacterial genotypes should not be assumed to represent adaptation to distinct niches.

## Supplementary information

Supplementary material
