# Supplementary material for: Negative frequency-dependent selection and asymmetrical transformation stabilise multi-strain bacterial population structures
Source: ISME J. 2021 Jan 6;15(5):1523–38. doi: 10.1038/s41396-020-00867-w (PMC8115253; doi:10.1038/s41396-020-00867-w)
Supplement: Supplementary file 1 — Supplementary material [file 41396_2020_867_MOESM1_ESM.pdf]

Supplementary Information

Text S1: Input data for simulations

The input data for simulations, unless otherwise specified, were the 616 genomes from the Massachusetts *S. pneumoniae* collection [1], processed as described previously [2]. Only the 1,090 accessory loci present at intermediate frequencies (*i.e.* between 5% and 95%) in the peri-vaccination population were included as being under NFDS in the simulations. For simulating the evolution of core genome variation, 1,090 biallelic core genome single nucleotide polymorphisms (SNPs), with minor allele frequencies above 5%, were randomly selected from the genomic data to be included in the simulations. Hence the same number of core and accessory loci were analysed, to simplify the comparison of pairwise distances calculated from $g_{i,l}$ and $c_{i,s}$. At each SNP site, the allele denoted as '0' matched that within the sequence of *S. pneumoniae* ATCC 700669 [3], such that the SNP frequency at time t ($f_{s,t}$) was that of the alternative allele. This ensured core and accessory allele frequencies were distributed over similar ranges.

Text S2: Calculation of parameter values

The e_l were calculated from the peri-vaccination sample of the Massachusetts *S. pneumoniae* population [1, 2]. To enable parameters estimated from previous model fits to this population to be used with these simulations [2], each generation corresponded to a month, and the carrying capacity κ was set to 10^5. This population size corresponds to the estimated number of *S. pneumoniae* hosts in the region over which the isolates were collected. This was assumed to be a more accurate representation of the effective population size than the census count of all *S. pneumoniae* cells in the surveyed region. This is because the bacterium has a small within-host effective population size [4], and undergoes frequent bottlenecks during transmission [5], so any genetic variation arising within a host is likely to be lost or fixed by the point at which the genotype has been acquired by the next host in the transmission chain. This loss of within-host diversity is implicitly assumed to occur every generation in the model, as each timestep corresponds to one month, which is approximately the interval at which transmissions between hosts occur [6]. Additionally, this population size assumed a degree of confinement to a particular area, as international variation in *S. pneumoniae* epidemiology suggests population dynamics are localised [7].

NFDS acted homogeneously on each accessory locus. The value σ_f used in multi-locus NFDS simulations (0.0356 month^{-1}) was calculated from the fitted model parameters as a weighted mean of the strong (σ_f = 0.1363 month^{-1}) and weak (σ_w = 0.0023 month^{-1}) NFDS strengths, according to the fraction (p_f = 0.2483) subject to σ_f [2]. Simulations were also run with ‘weak NFDS’, using only the latter value (σ_w = 0.0023 month^{-1}).

The rate of inter-strain transformation was based on the best-fitting homogeneous rate recombination model (model 2: null model with over-dispersion) from a modelling study of divergence through recombination within *S. pneumoniae* strains [8]. Transformations detectable through exchanging sequence variation between strains were estimated to occur

at a mean rate, τ, of 0.21 y^{-1} (τ = 0.0175 $month^{-1}$) and span 6.4 kb of the genome. This rate does not include within-strain transformations, which are unlikely to be detectable through sequence divergence. Additionally, as these values were inferred from isolate collections, they correspond to the post-selection rate, and therefore underestimate the actual transformation rate.

Across the 616 genomes in the Massachusetts dataset, isolates encoded a mean of 309 intermediate-frequency loci. As an *S. pneumoniae* genome contains ~2,000 genes [1], this implies intermediate-frequency loci comprise ~15% of the genome. Therefore the monthly whole genome transformation rate (τ = 0.0175 $month^{-1}$) was scaled to represent the rate with which transformation would affect only the intermediate-frequency loci, assuming an homogeneous distribution of transformation events (τ = 0.002625 $month^{-1}$).

The typical transformation event size (6.4 kb [8]) would be consistent with the acquisition or deletion of a 5 kb accessory locus, given that transformation events affecting accessory loci typically include two flanking homologous arms of 0.75-1 kb [9]. Such an homologous recombination would correspond to approximately five intermediate-frequency genes, given each *S. pneumoniae* gene has a length of ~1 kb [3]. As each isolate encoded approximately 300 accessory loci, the proportion of loci affected during an exchange through transformation (ϱ) was set to 0.0167, such that the expected number of loci present in the donor and recipient potentially affected by transformation was approximately five. Given this size of accessory locus and the experimentally-determined relationship between the efficacy of insertion relative to SNP transfer [9], the magnitude of transformational asymmetry (φ) was estimated to be 0.05.

The product τρ, representing the mean rate at which transformation affects each *S. pneumoniae* accessory locus, dictates the timescale over which the effects of recombination are detectable. The calculated value of 4.38×10^{-5} $month^{-1}$ indicated each locus would only be

expected to be affected by transformation in a given isolate once every ~2,000 years. Therefore simulations were run for 60,000 generations (equivalent to ~5,000 years).

Transformation appears to be a saltational process, with substantial inter-strain exchanges occurring infrequently [8]. These rare, but extensive, recombination events may play an important role in the emergence of strains [10]. To model this, simulations were run in which τ was reduced five-fold (τ = 0.000525 month^{-1}), and ρ increased five-fold (0.0835), such that the mean transformation rate per locus ($\tau\rho$) remained constant.

Text S3: Details of additional simulations

Initialisation of simulated populations

All simulations were initialised with a population of size κ generated through sampling input genotypes with replacement. For analyses using permuted genotypes, each simulation was initialised with an independently-generated set of genotypes in which the alleles at each locus (i.e., the columns of the $g_{i,l}$ and $c_{i,s}$ matrices) had been separately shuffled. Hence the genotypes were expected to be in linkage equilibrium, but each allele remained at the same initial frequency as in the genomic data. For analyses using randomised genotypes, each simulation was initialised with an independently-generated set of genotypes in which all alleles in each individual (i.e., all elements in the $g_{i,l}$ and $c_{i,s}$ matrices) were selected to be zero or one, with equal probabilities. Hence the genotypes were again expected to be in linkage equilibrium, and each allele had an initial frequency of approximately 0.5.

For analyses using a reduced subset of the accessory loci, all simulations used the same ten loci to enable the results to be combined and plotted. These were selected to be uniformly spaced across the range of intermediate frequencies, to control against any observed effects being specific to loci that were near one extreme of the distribution.

Simulation of migration

The strain composition of *S. pneumoniae* populations sampled from different locations varies extensively [2, 7]. Hence simulations were run with inward migration from ten external populations, to reflect the effects of genotypes moving within a geographically-structured meta-population. High rates of migration would be expected to cause all communities within a metapopulation to homogenise [11]. Hence m was set at 10^{-5}, such that under neutral evolution it would be expected that the majority of isolates in a population at the end of the simulations would not have been imported from other sources (i.e., $(1-m)^{60000} > 0.5$).

For each analysed combination of parameters, ten independent replicate source populations were generated prior to the reported simulations. Each of these was produced by running a series of simulations, each for k generations, in which the final generation of one simulation was used to generate the starting population of the next. These simulations were themselves of closed populations. For this analysis, $k = 600$, and the series of simulations was run for the same overall number of generations as the analysed simulations (60,000 generations). At the end of each k generations, 5,000 genotypes were randomly sampled from the final population, without consideration of its categorisation into strains. These were used to generate a population of size κ through sampling with replacement to initiate the next phase of k generations. Once these serial simulations were complete, ten isolates were randomly drawn from each sampling timepoint (after each k generations) in each of the ten replicates.

This process generated pools of 100 migrants, denoted j_k, $j_{2k}\ldots j_{nk}$, each of which corresponded to a particular timestep of simulations run with a specified parameter set. These were supplemented with 100 randomly-selected genotypes from the genomic data used as the starting population, to provide a j_0 pool representing isolates in the early timesteps. When the analysed simulations featuring migration were run, the pool of genotypes from which migrants were drawn changed over time. Hence at generation t, while $nk \leq t < (n+1)k$, the migrant genotypes were selected from j_{nk}. Synchronising the immigrating and resident bacteria ensured migration did not artefactually disrupt long-term evolutionary trends in the simulations, such as the decay of the accessory genome in neutral simulations featuring asymmetric transformation.

The analysed simulations were each run as the equivalent simulations of closed populations, except that at each generation t, M_t isolates were imported into the simulated population at a rate determined by the parameter m:

$$M_t \sim Bin(m, \kappa)$$

Hence the reproduction function was changed to:

$$X_{i,t} \sim Pois\left(\left(\frac{\kappa}{N_t}\right)(1-m)\big(1+\sigma_f\big)^{\pi_{i,t}}\right)$$

Each isolate in the pool j_{nk} was equally likely to be randomly drawn to contribute to the M_t imported isolates.

Text S4: Statistical analyses of simulation outputs

Analyses and visualisations used R [12] with tidyverse [13], ggrastr [14] and ggpubr [15] packages. Pairwise genetic distance calculations were performed with rdist [16]. Distributions were analysed with propagate [17] and quantileDA [18]. Neighbour-joining trees were constructed using ape [19] and visualised with ggtree [20]. Pybus and Harvey's ɣ [21] was calculated using ape after trees were converted to be ultrametric using phytools [22]. Strains were defined using the genetic distance threshold indicated in Fig. 4 by constructing networks using igraph [23, 24]. Permutations of gene presence and absence were conducted with vegan [25].

Text S5: Limitations and assumptions of the model

The model did not include any introduction of novel variation through mutation, or horizontal gene transfer originating from external populations. Such emergences were difficult to incorporate, as only intermediate-frequency alleles were modelled. Such loci are unlikely to have been recently generated or acquired, as they are shared between distant populations with divergent strain compositions [2]. However, given the timescales of the simulations, it is likely that at least some loci would become polymorphic with minor allele frequencies above 5%. The inclusion of such processes could enable strain formation through divergence driven by an accumulation of novel accessory loci and SNPs. Yet analysis of *S. pneumoniae* populations suggests strains have little private gene content, instead being differentiated by their distinctive combinations of common loci [26]. Hence the accumulation of novel polymorphic loci is important to bacterial evolution, but may not be necessary for the generation of MSP structures.

Additionally, exchange of sequence through recombination was underestimated in these simulations. Interstrain transformation occurred at a single rate that was inferred from reconstructions of clinical isolates' evolutionary history [8], which is necessarily measured after selection. Hence the actual pre-selection rate of transformation is higher, although this should not qualitatively alter the results, unless it were high enough to be predicted to drive the elimination of some accessory loci.

The transformation rate was simulated as being uniform across genotypes, between loci and over time. This does not account for the variation in interstrain transformation rate observed across the species [1, 7], nor the apparent 'hotspots' of recombination within the chromosome [27, 28]. Additionally, this does not reflect the punctuate nature of interstrain recombination [8], which was approximated by simulations with 'saltational' transformation. It was assumed that these large, infrequent recombinations had the same properties as more common transformation events, but it is possible they would not exhibit the same deletional

bias. Large, more symmetrical exchanges would increase the rate of new strain formation, based on the diverse, unstructured populations generated by simulations combining multi-locus NFDS with symmetrical transformation. This would increase the necessity for a mechanism driving outbreeding depression to preserve MSPs. Correspondingly, the simulations initiated with permuted or randomised initial populations demonstrated that the combination of multi-locus NFDS and asymmetrical transformation can restore a unstructured population's division into strains.

In each exchange through transformation within the model, the number of loci affected by recombination was determined by a fixed parameter applied at a constant rate per site. However, transformation events in the core genome have an approximately exponential length distribution [29], consistent with greater variance in the number of SNPs being affected by each exchange between divergent genotypes in the population. The effect of transformation on accessory loci depends on how they are arranged within the chromosome as genomic islands, as short recombinations can nevertheless delete many accessory loci if they are present in the recipient as a contiguous stretch of DNA absent from the donor [9]. However, in these simulations all loci evolved independently, without consideration of the chromosomal architecture. Hence it is likely that the model underestimates the variation in the amount of recipient sequence affected by each exchange between cells, as well as the heterogeneity in the rate of such exchanges within the population.

The assumption of independently-evolving loci, without considering the linkage of non-mobile accessory loci into genomic islands [2, 26], had further implications for the analysis. Genomic islands are found in many combinations across *S. pneumoniae* population, which is consistent with the "modular selection" model of local epistasis [30]. The coherence of these distinct islands would decrease the number of possible accessory locus genotypes, and increase the variance and heterogeneity of the locus frequency, genome size and pairwise distance distributions generated by simulations featuring transformation.

Additionally, as the extent of transformational asymmetry is determined by the size of a genomic island [9], and the selective pressure acting upon it will be determined by the functions of the encoded genes, this means the prevention of decay by NFDS will actually be a property of each island, rather than being uniform across the population.

Contrasting with the strong linkage between accessory loci within a genomic island, there is little species-wide linkage between these islands and the proximal core genome SNPs in *S. pneumoniae* [2]. Hence there is limited evidence for localised “fronts” of diversification surrounding genomic islands, as was previously hypothesised to flank structural variation [31–33]. Therefore, the extent to which neutral SNP frequencies were preserved through selection on accessory loci should represent a minimum, that may be slightly higher in more realistic simulations that account for the limited linkage between some core and accessory variation.

Many genomic islands, and by implication the accessory loci within then, are autonomously mobile [2]. Therefore the net asymmetry of the recombination processes affecting their distribution will favour insertion over deletion. This could be simulated using a parameterisation of $\varphi > 1$. Assuming such elements to be parasitic, recombinant progeny would likely be outcompeted by the original genotype, which does not harbour the element. This would be consistent with the long-standing hypothesis that the steady-state frequency of ‘selfish’ elements reflects a balance between their mobility and selection against infected genotypes [34].

As well as the genetic simplifications, the simulations also assumed ecological homogeneity. The selection pressures on the population depended on the equilibrium frequencies of the accessory loci. These were identical for all genotypes, and were consistent over the duration of the simulations. This is in contrast to the observation that public health interventions can cause differences in these equilibrium frequencies, such as by suppressing loci through

vaccine-induced immunity, or antibiotic resistance loci being sustained at different levels in distinct populations [2]. Over the long timescales of the simulations, it is plausible that other processes may be important. For instance, “Red Queen” dynamics might drive variation in the mobile elements that infect *S. pneumoniae*, with concomitant changes to the loci comprising the cell’s defences against infection [35, 36]. These types of changes could destabilise the populations evolving under multi-locus NFDS. Such an effect might be amplified by changes in the equilibrium frequency of one locus affecting those of others. This could be the result of phenotypic interactions, or because different genomic islands compete for orthologous locations in the chromosome. Examples of the latter are the capsule polysaccharide synthesis loci in *S. pneumoniae*, which almost all insert at the same location in the chromosome [37]; vaccine-induced elimination of particular *S. pneumoniae* capsule types might therefore be expected to result in an increase in the frequency of loci encoding for the production of other capsule types. Such effects were not included in the model, which assumed all equilibrium frequencies to be independent.

Based on the results from simulations initialised with randomised genotypes, the consequences of changes in equilibrium frequency would depend on the level of recombination in the population (Fig. S5). In the absence of transformation, clonal interference appeared to prevent all loci from reaching their equilibrium frequencies simultaneously [38]. However, in simulations featuring either symmetrical or asymmetrical transformation, all loci reached their equilibrium frequencies. Furthermore, the consistency with which the characteristics of MSP structures emerged from simulations combining asymmetric transformation with multi-locus NFDS, despite differences in the initial locus frequencies (Fig. 4, S26, S28), suggests the overall properties of the population would be robust to alterations in equilibrium gene frequencies. The exception is the situation in which a high proportion of intermediate frequency loci were lost from the population. This would be likely to result in a reduction in the population’s strain diversity, based on the simulations run with a reduced subset of loci under NFDS (Fig. S49).

A further limitation of the simulations was the implementation as an individual-based model using a Wright-Fisher framework. This enabled individual genotypes to be modified through transformation over the course of simulations, but made the model computationally intensive to run. Hence the number of generations it was feasible to analyse was not sufficient for neutral simulations to reach an equilibrium (Fig. S1). It is also likely that the simulations featuring transformation that were initialised with permuted or randomised genotypes had not yet reached a final equilibrium state, given the relatively slow rate of transformation.

Supplementary References

1. Croucher NJ, Finkelstein JA, Pelton SI, Mitchell PK, Lee GM, Parkhill J, et al. Population genomics of post-vaccine changes in pneumococcal epidemiology. *Nat Genet* 2013; **45**: 656–663.
2. Corander J, Fraser C, Gutmann MU, Arnold B, Hanage WP, Bentley SD, et al. Frequency-dependent selection in vaccine-associated pneumococcal population dynamics. *Nat Ecol Evol* 2017; **1**: 1950–1960.
3. Croucher NJ, Walker D, Romero P, Lennard N, Paterson GK, Bason NC, et al. Role of conjugative elements in the evolution of the multidrug-resistant pandemic clone *Streptococcus pneumoniae*^{Spain23F} ST81. *J Bacteriol* 2009; **191**: 1480–1489.
4. Li Y, Thompson CM, Trzciński K, Lipsitch M. Within-host selection is limited by an effective population of *Streptococcus pneumoniae* during nasopharyngeal colonization. *Infect Immun* 2013; **81**: 4534–4543.
5. Weiser JN, Ferreira DM, Paton JC. *Streptococcus pneumoniae*: Transmission, colonization and invasion. *Nat Rev Microbiol* 2018; **16**: 355–367.
6. Lees JA, Croucher NJ, Goldblatt D, Nosten F, Parkhill J, Turner C, et al. Genome-wide identification of lineage and locus specific variation associated with pneumococcal carriage duration. *Elife* 2017; **6**: e26255.
7. Gladstone RA, Lo SW, Lees JA, Croucher NJ, van Tonder AJ, Corander J, et al. International genomic definition of pneumococcal lineages, to contextualise disease, antibiotic resistance and vaccine impact. *EBioMedicine* 2019; **43**: 338–346.
8. Mostowy R, Croucher NJ, Hanage WP, Harris SR, Bentley S, Fraser C. Heterogeneity in the Frequency and Characteristics of Homologous Recombination in Pneumococcal Evolution. *PLoS Genet* 2014; **10**: e1004300.
9. Apagyi KJ, Fraser C, Croucher NJ. Transformation asymmetry and the evolution of the bacterial accessory genome. *Mol Biol Evol* 2018; **35**: 575–581.
10. Croucher NJ, Klugman KP. The emergence of bacterial ‘Hopeful monsters’. *MBio*

2014; **5**.

11. Kareiva P. Population dynamics in spatially complex environments: theory and data. *Philos Trans - R Soc London, B* 1990; **330**: 175–190.
12. R Core Team. R: A Language and Environment for Statistical Computing. *R Found Stat Comput* 2019.
13. Wickham H, Averick M, Bryan J, Chang W, McGowan L, François R, et al. Welcome to the Tidyverse. *J Open Source Softw* 2019; **4**: 1686.
14. Petukhov V, van den Brand T, Biederstedt E. ggrastr: Raster Layers for 'ggplot2'. 2020.
15. Kassambara A. ggpubr: 'ggplot2' Based Publication Ready Plots. 2020.
16. Blaser N. rdist: Calculate Pairwise Distances. 2018.
17. Spiess A-N. propagate: Propagation of Uncertainty. 2018.
18. Hennig C, Viroli C. quantileDA: Quantile Classifier. 2016.
19. Paradis E, Schliep K. ape 5.0: an environment for modern phylogenetics and evolutionary analyses in R. *Bioinformatics* 2018; **35**: 526–528.
20. Yu G, Smith DK, Zhu H, Guan Y, Lam TTY. ggtree: an R package for visualization and annotation of phylogenetic trees with their covariates and other associated data. *Methods Ecol Evol* 2017; **8**: 28–36.
21. Pybus OG, Harvey PH. Testing macro-evolutionary models using incomplete molecular phylogenies. *Proc R Soc B Biol Sci* 2000; **267**: 2267–2272.
22. Revell LJ. phytools: An R package for phylogenetic comparative biology (and other things). *Methods Ecol Evol* 2012; **3**: 217–223.
23. Csardi G, Nepusz T. The igraph software package for complex network research. *InterJournal* 2006; **Complex Sy**.
24. Lees JA, Harris SR, Tonkin-Hill G, Gladstone RA, Lo S, Weiser JN, et al. Fast and flexible bacterial genomic epidemiology with PopPUNK. *Genome Res* 2019; **29**: 304–316.
25. Dixon P. VEGAN, a package of R functions for community ecology. *J Veg Sci* 2003;

14: 927–930.

26. Croucher NJ, Coupland PG, Stevenson AE, Callendrello A, Bentley SD, Hanage WP. Diversification of bacterial genome content through distinct mechanisms over different timescales. *Nat Commun* 2014; **5**: 5471.

27. Croucher NJ, Harris SR, Fraser C, Quail MA, Burton J, van der Linden M, et al. Rapid pneumococcal evolution in response to clinical interventions. *Science* 2011; **331**: 430–434.

28. Croucher NJ, Campo JJ, Le TQ, Liang X, Bentley SD, Hanage WP, et al. Diverse evolutionary patterns of pneumococcal antigens identified by pangenome-wide immunological screening. *Proc Natl Acad Sci U S A* 2017; **114**: E357–E366.

29. Croucher NJ, Harris SR, Barquist L, Parkhill J, Bentley SD. A high-resolution view of genome-wide pneumococcal transformation. *PLoS Pathog* 2012; **8**: e1002745.

30. Neher RA, Shraiman BI. Competition between recombination and epistasis can cause a transition from allele to genotype selection. *Proc Natl Acad Sci U S A* 2009; **106**: 6866–6871.

31. Lawrence JG. Gene Transfer in Bacteria: Speciation without Species? *Theor Popul Biol* 2002; **61**: 449–460.

32. Vetsigian K, Goldenfeld N. Global divergence of microbial genome sequences mediated by propagating fronts. *Proc Natl Acad Sci U S A* 2005; **102**: 7332–7337.

33. Nosil P, Funk DJ, Ortiz-Barrientos D. Divergent selection and heterogeneous genomic divergence. *Mol Ecol* 2009; **18**: 375–402.

34. Orgel LE, Crick FHC. Selfish DNA: The ultimate parasite. *Nature* 1980; **284**: 604–7.

35. Judson OP. Preserving genes: A model of the maintenance of genetic variation in a metapopulation under frequency-dependent selection. *Genet Res* 1995; **65**: 175–191.

36. Takeuchi N, Cordero OX, Koonin E V, Kaneko K. Gene-specific selective sweeps in bacteria and archaea caused by negative frequency-dependent selection. *BMC Biol* 2015; **13**: 20.

37. Bentley SD, Aanensen DM, Mavroidi A, Saunders D, Rabbinowitsch E, Collins M, et

al. Genetic analysis of the capsular biosynthetic locus from all 90 pneumococcal serotypes. *PLoS Genet* 2006; **2**: e31.

38. Maddamsetti R, Lenski RE, Barrick JE. Adaptation, clonal interference, and frequency-dependent interactions in a long-term evolution experiment with *Escherichia coli*. *Genetics* 2015; **200**: 619–631.

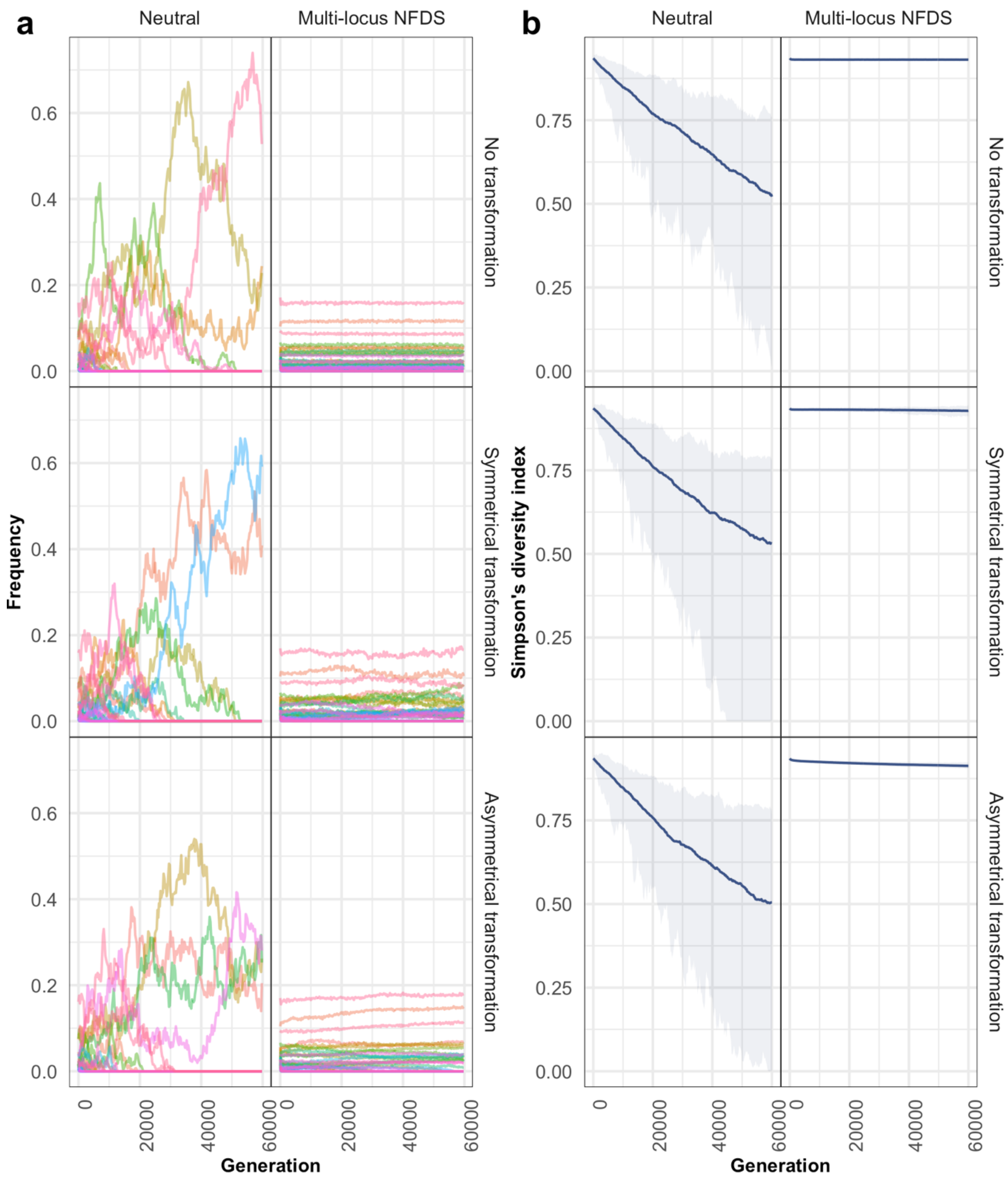

Figure S1: Plots describing the dynamics of simulated populations. All plots summarise the diversity of the population using the strains to which genotypes were assigned in the input genomic data, and therefore they do not reflect within-strain diversification occurring in simulations featuring transformation. **a** Line plots showing the frequencies of strains (each represented by a different colour) during an example simulation. **b** Line plots showing the change in Simpson's diversity index. The solid line shows the mean value over 100 replicate simulations, and the shaded area shows the corresponding range of values per generation.

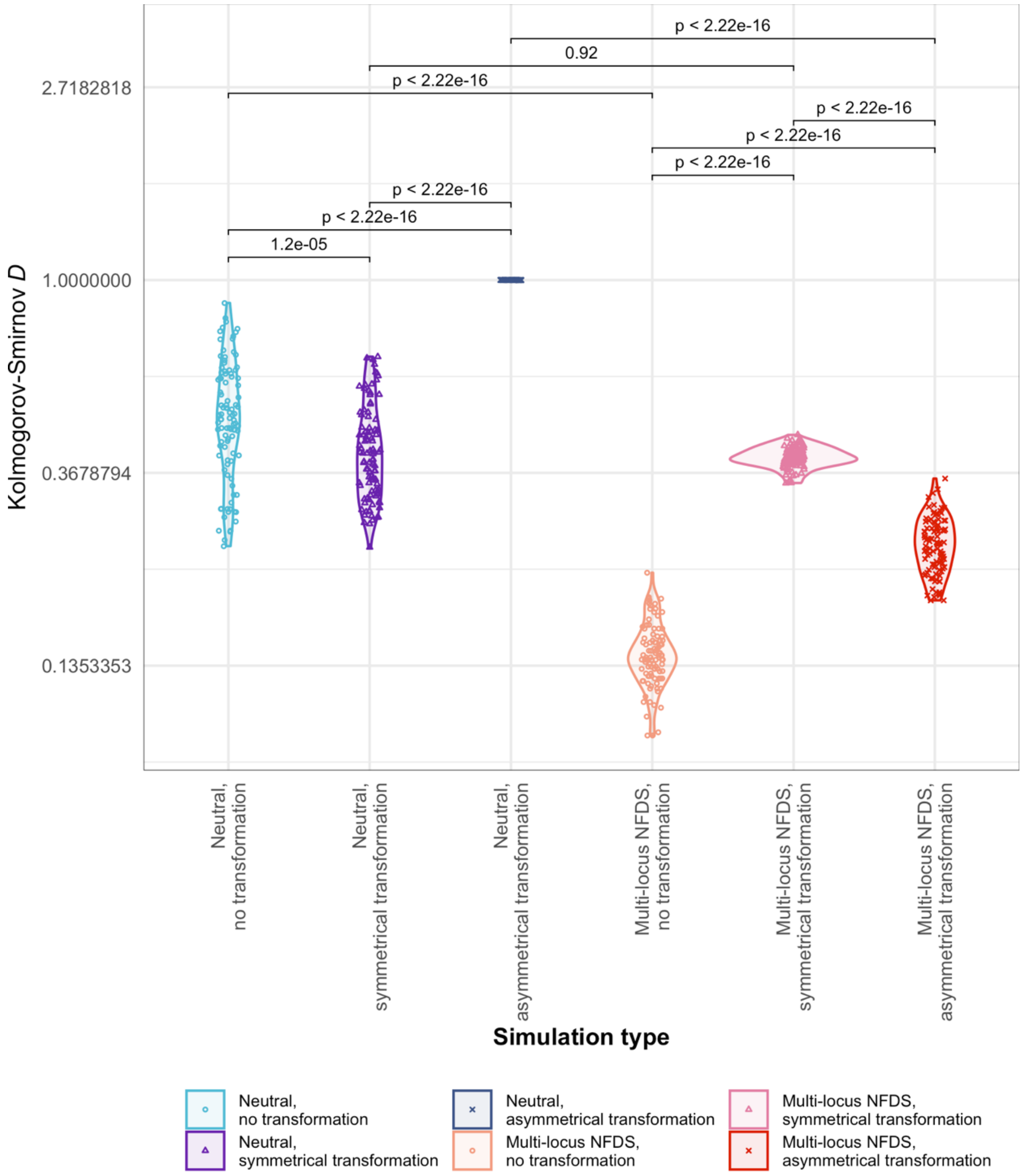

Figure S2: Violin plots comparing the observed distribution of intermediate-frequency loci per genome to those from the final timestep of simulations without migration (Fig. 2). The deviation between the observed and simulated data (*N* = 100 replicates for each parameter combination) was measured using the Kolmogorov-Smirnov *D* statistic. The values for the individual simulations are shown by points, and summarised over replicates as a violin plot. Pairwise Wilcoxon rank-sum tests were then used to compare the distribution of *D* statistics from sets of simulations that differed only in the mode of transformation or selection. The *p* values from these tests are annotated at the top of the chart.

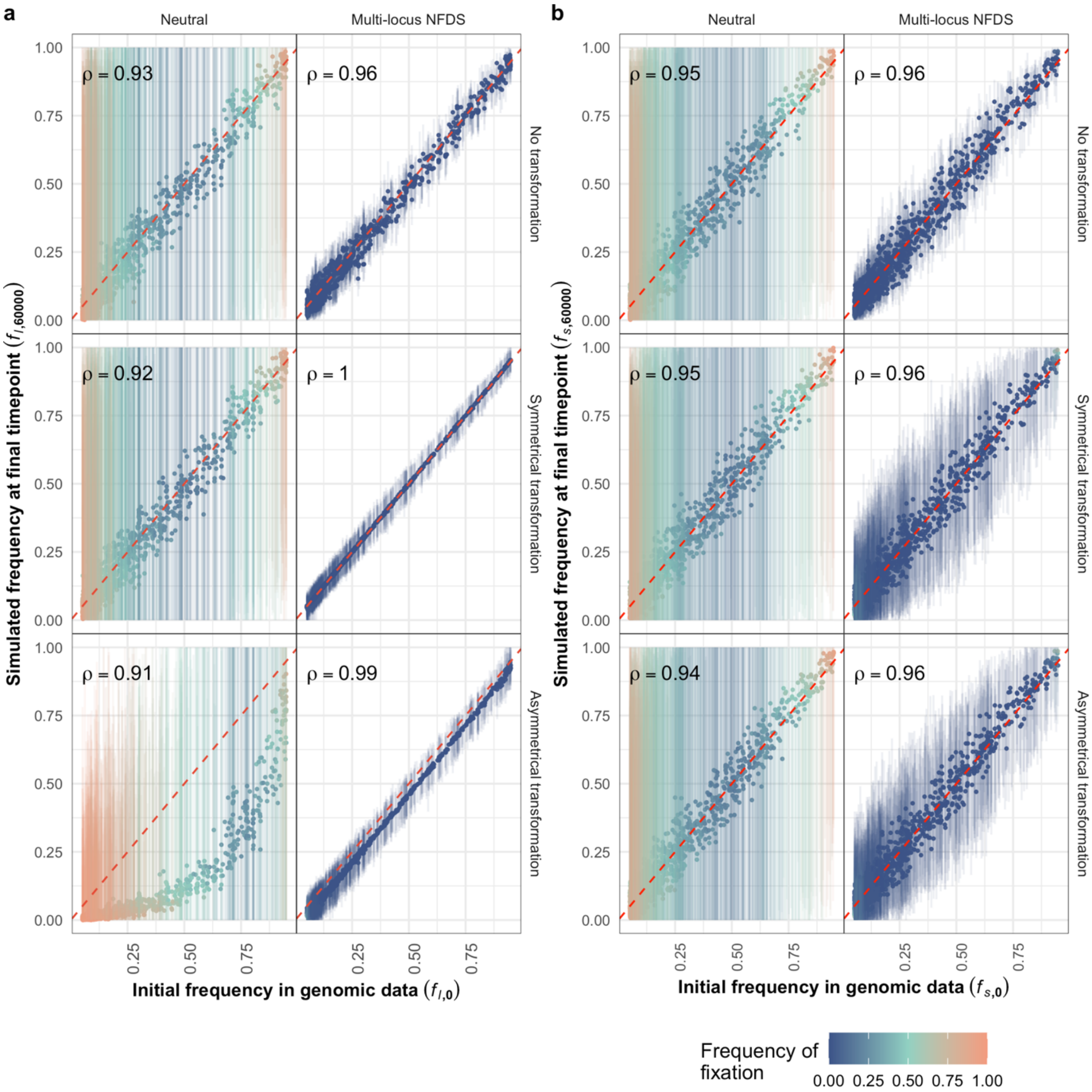

Figure S3: Scatterplots comparing the frequency of alleles at the initial timepoint in the genomic data to their frequency in the final simulation timepoint (N = 616 isolates sampled from each simulation). Data are displayed as in Fig. 1. These simulations were initiated with populations in which the alleles at each accessory locus, and SNP site, had been permuted across genotypes (Table 1).

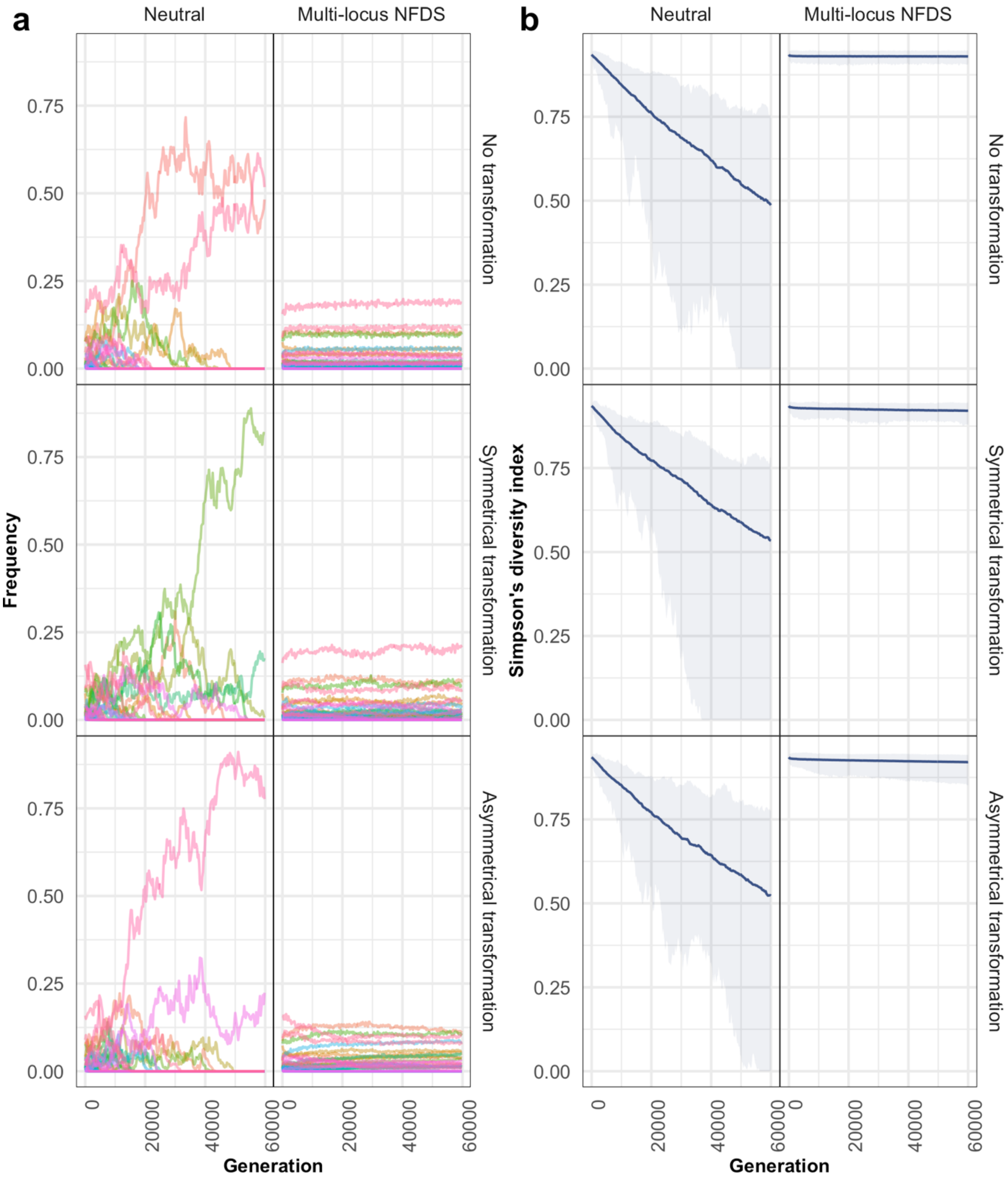

Figure S4: Plots describing the dynamics of simulated populations. Data are displayed as in Fig. S1. These simulations were initiated with populations in which the alleles at each accessory locus, and SNP site, had been permuted across genotypes (Table 1).

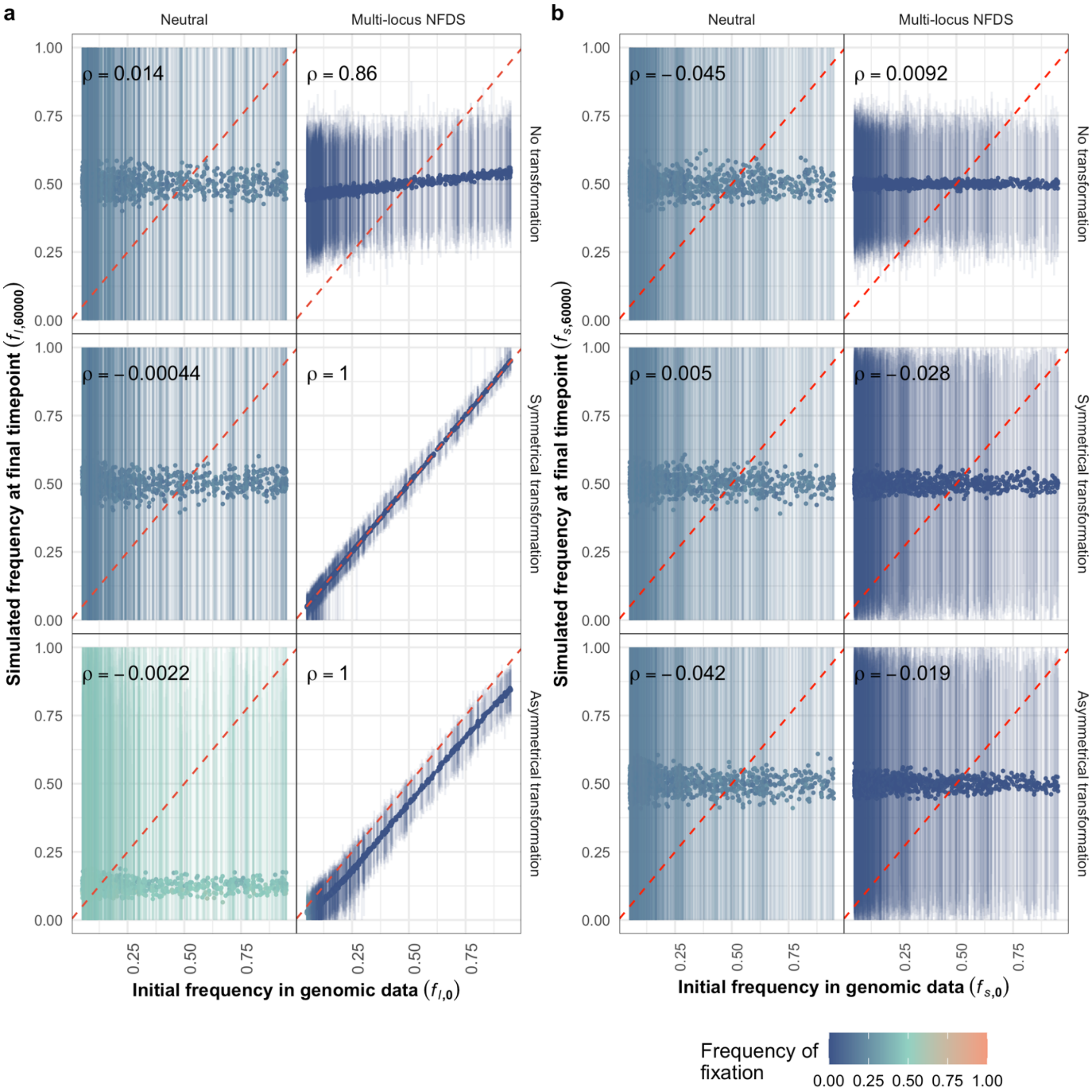

Figure S5: Scatterplots comparing the frequency of alleles at the initial timepoint in the genomic data to their frequency in the final simulation timepoint (N = 616 isolates sampled from each simulation). Data are displayed as in Fig. 1. These simulations were initiated with populations in which the alleles at each accessory locus, and SNP site, were randomly generated (Table 1).

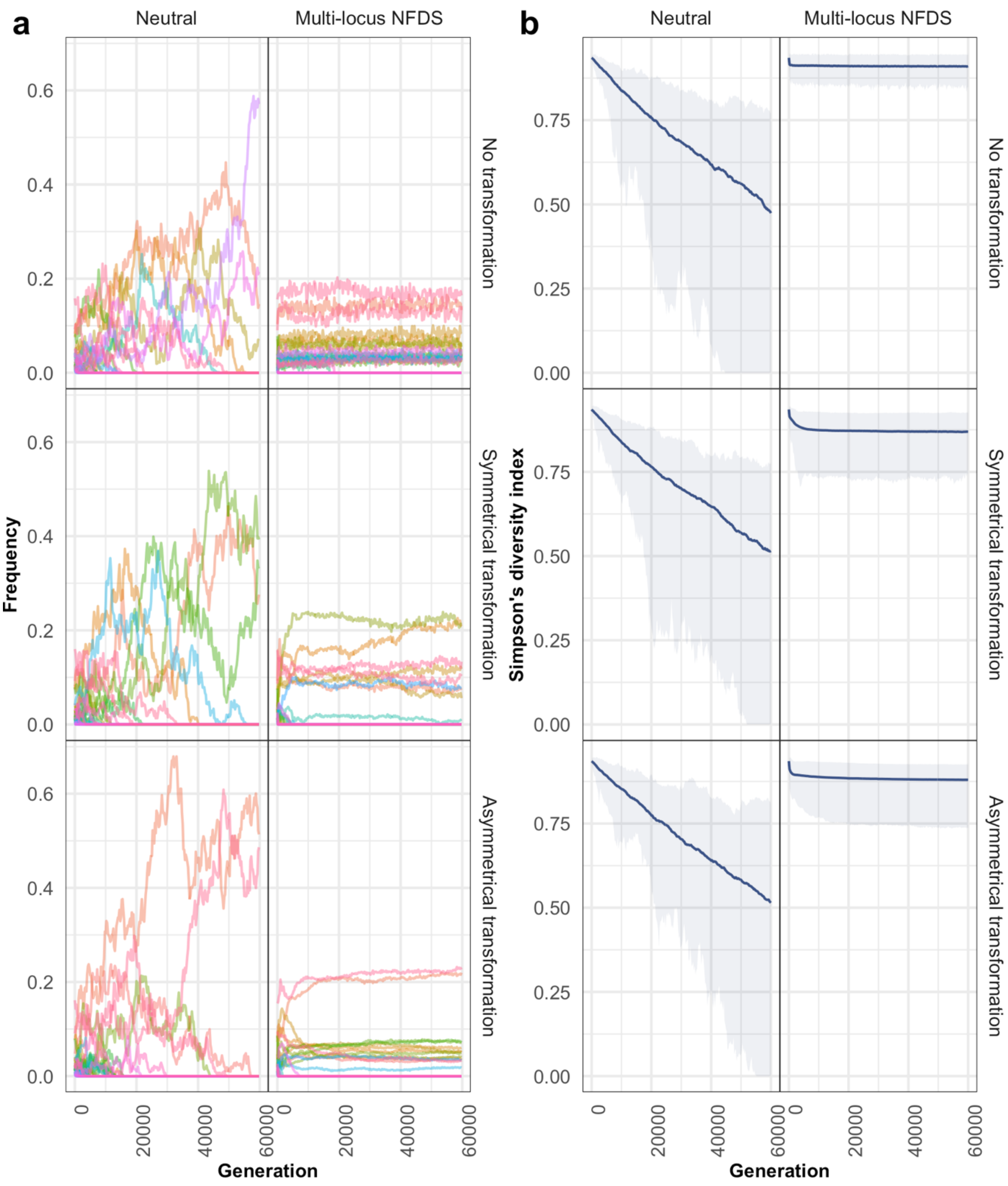

Figure S6: Plots describing the dynamics of simulated populations. Data are displayed as in Fig. S1. These simulations were initiated with populations in which the alleles at each accessory locus, and SNP site, were randomly generated (Table 1).

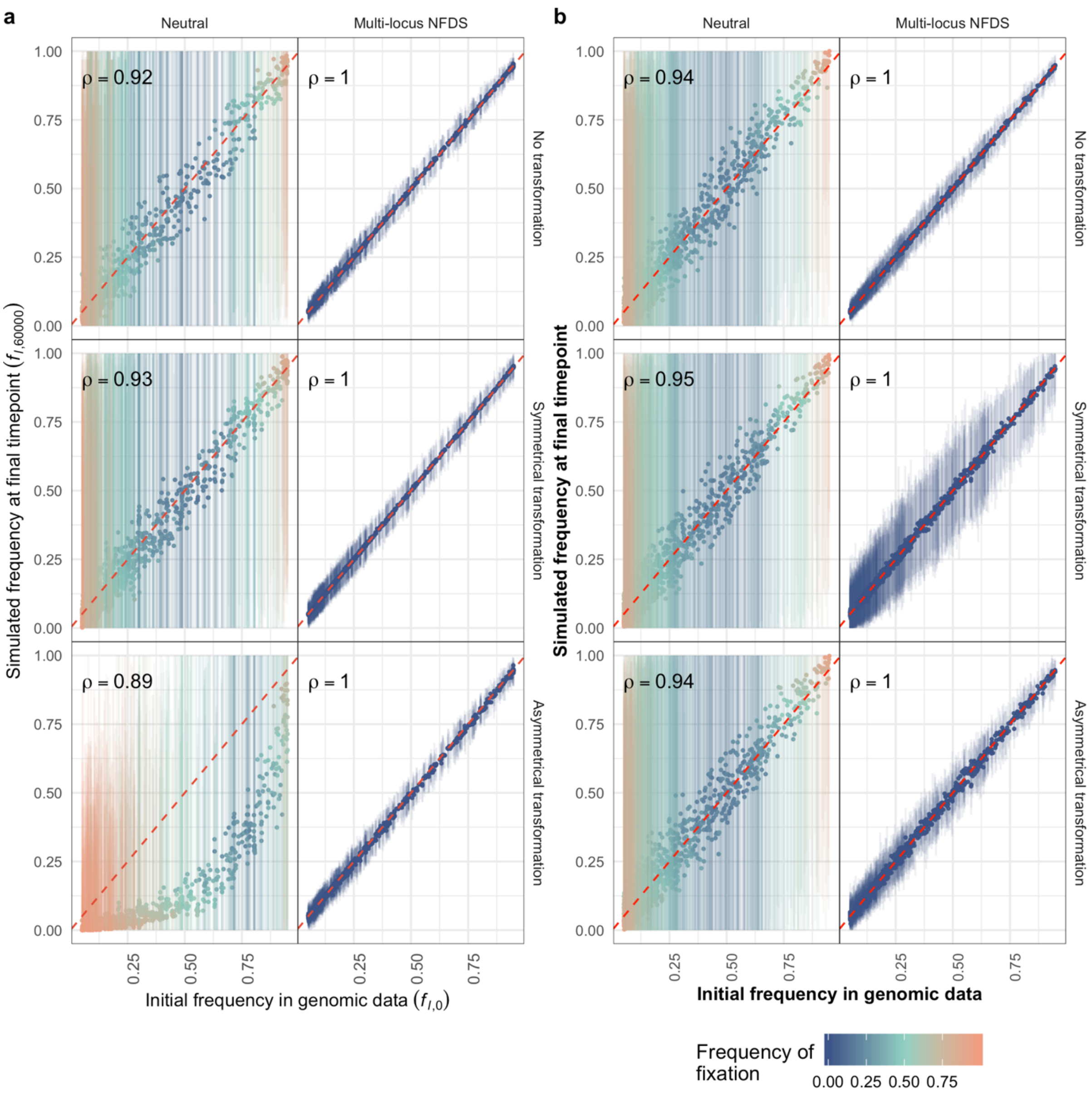

Figure S7: Scatterplots comparing the frequency of alleles in the initial timepoint in the genomic data to their frequency in the final simulation timepoint (N = 616 isolates sampled from each simulation). Data are displayed as in Fig. 1. These simulations featured saltational transformation (Table 1).

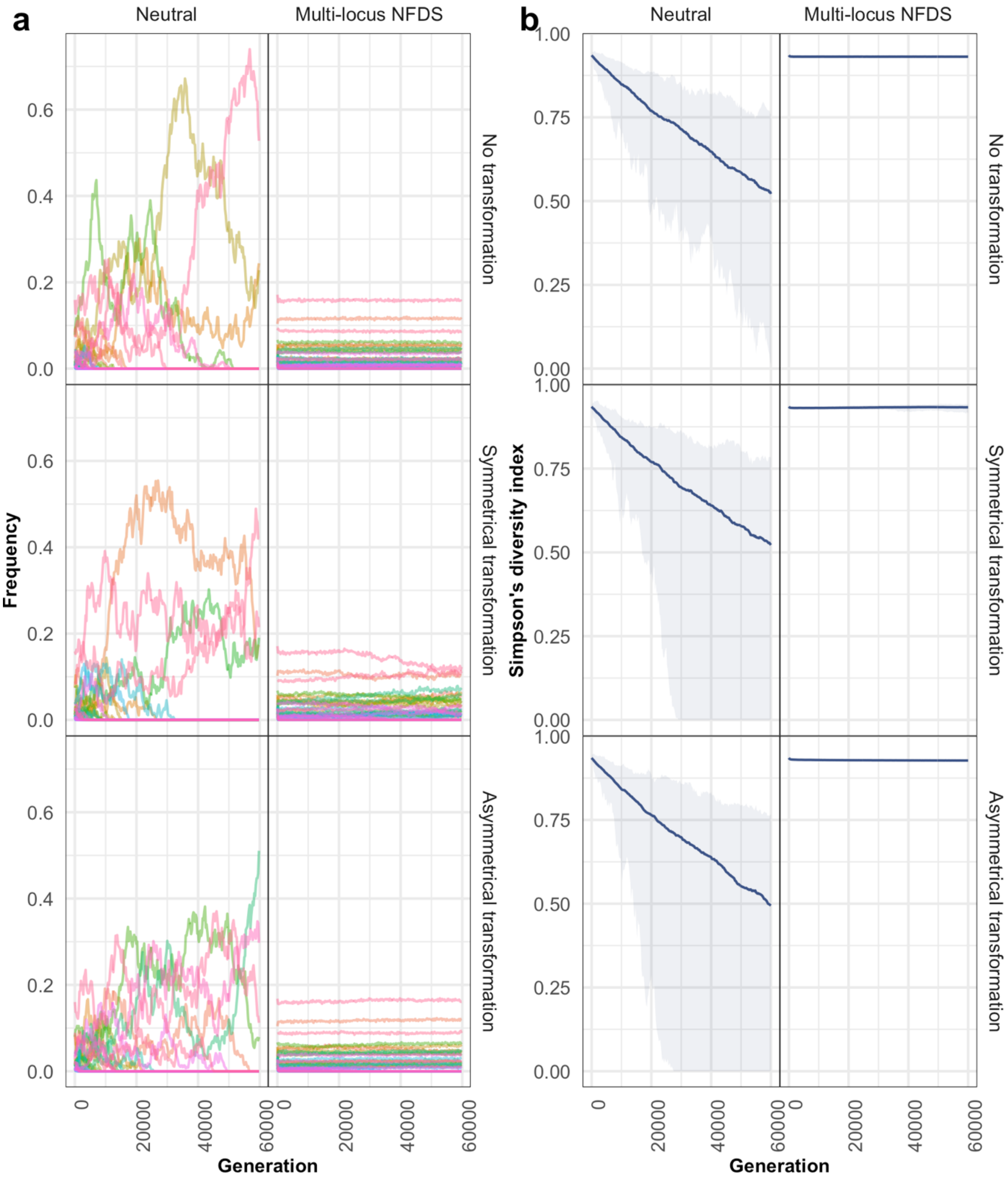

Figure S8: Plots describing the dynamics of simulated populations. Data are displayed as in Fig. S1. These simulations featured saltational transformation (Table 1).

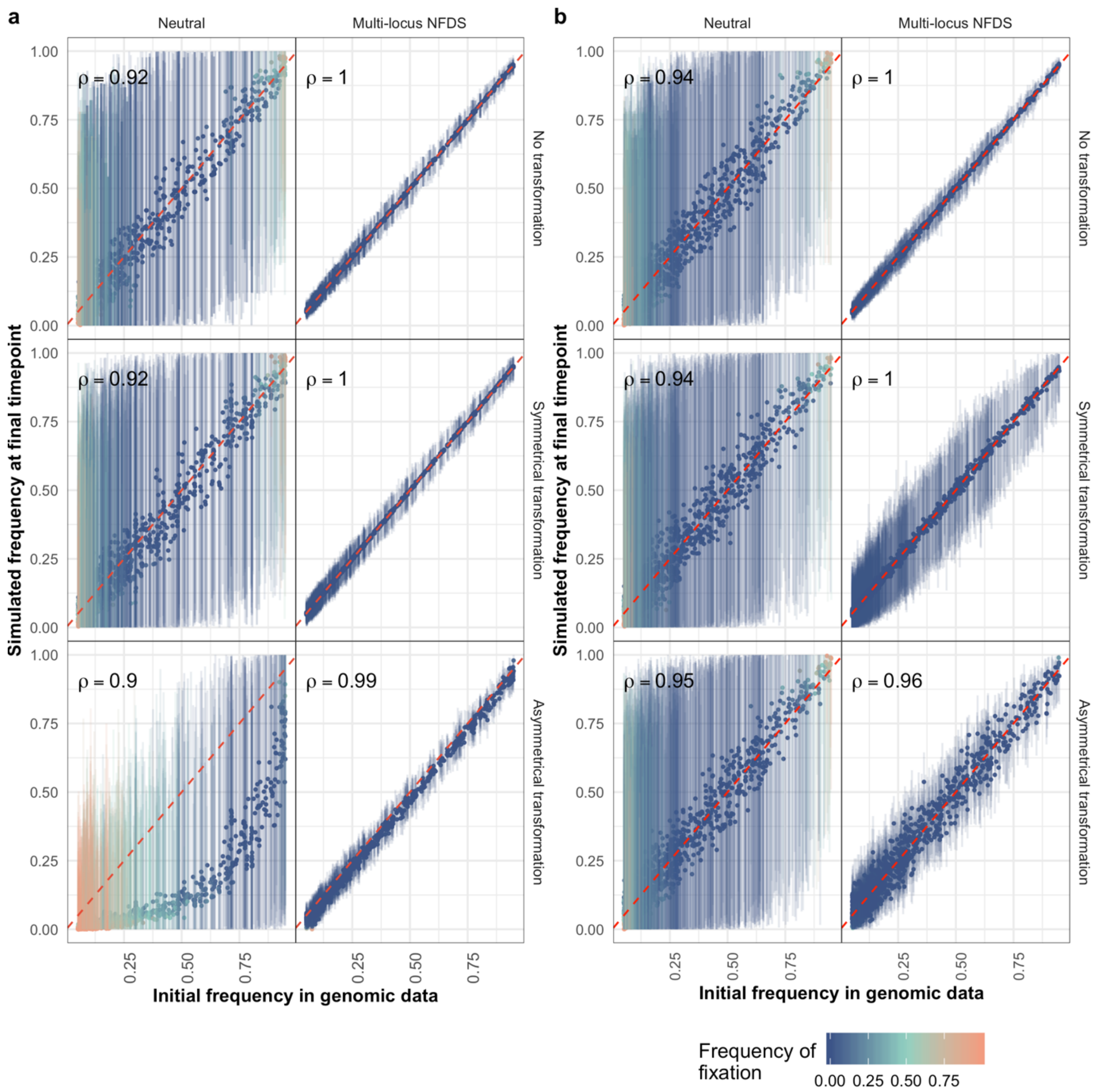

Figure S9: Scatterplots comparing the frequency of alleles at the initial timepoint in the genomic data to their frequency in the final simulation timepoint (*N* = 616 isolates sampled from each simulation). Data are displayed as in Fig. 1. These simulations featured inward migration (Table 1).

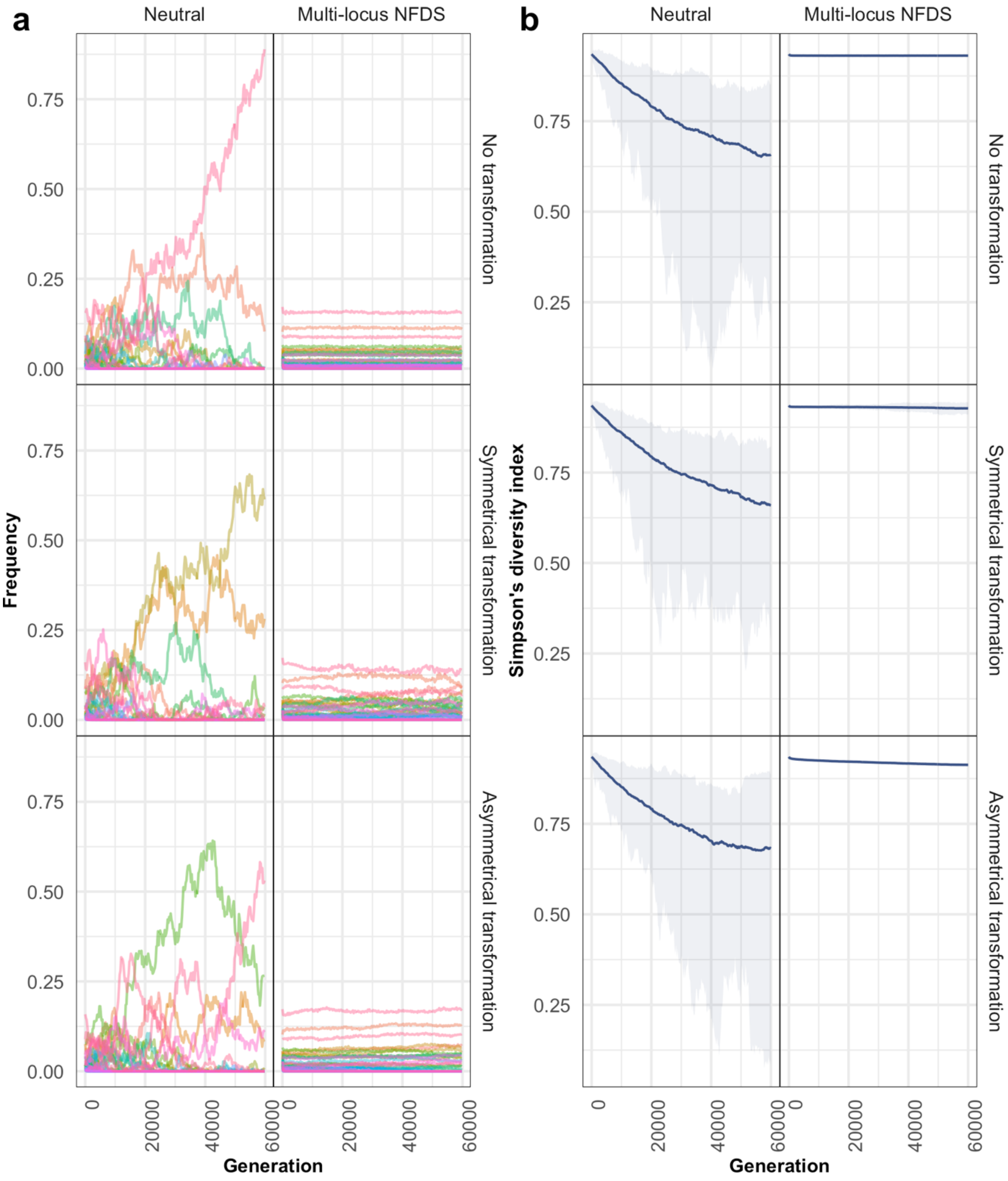

Figure S10: Plots describing the dynamics of simulated populations. Data are displayed as in Fig. S2. These simulations featured inward migration (Table 1).

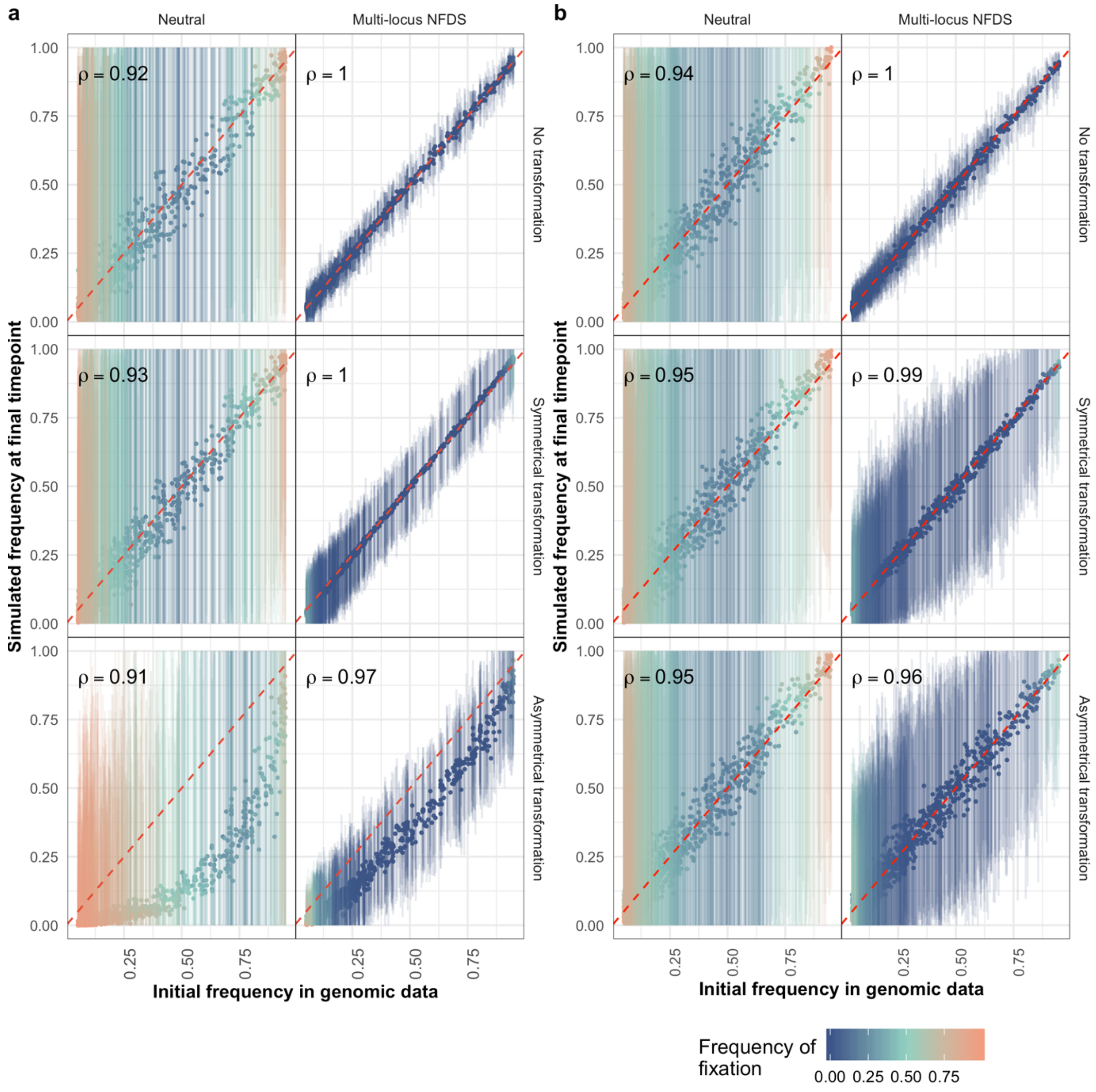

Figure S11: Scatterplots comparing the frequency of alleles at the initial timepoint in the genomic data to their frequency in the final simulation timepoint (*N* = 616 isolates sampled from each simulation). Data are displayed as in Fig. 1. These simulations featured weak multi-locus NFDS (Table 1).

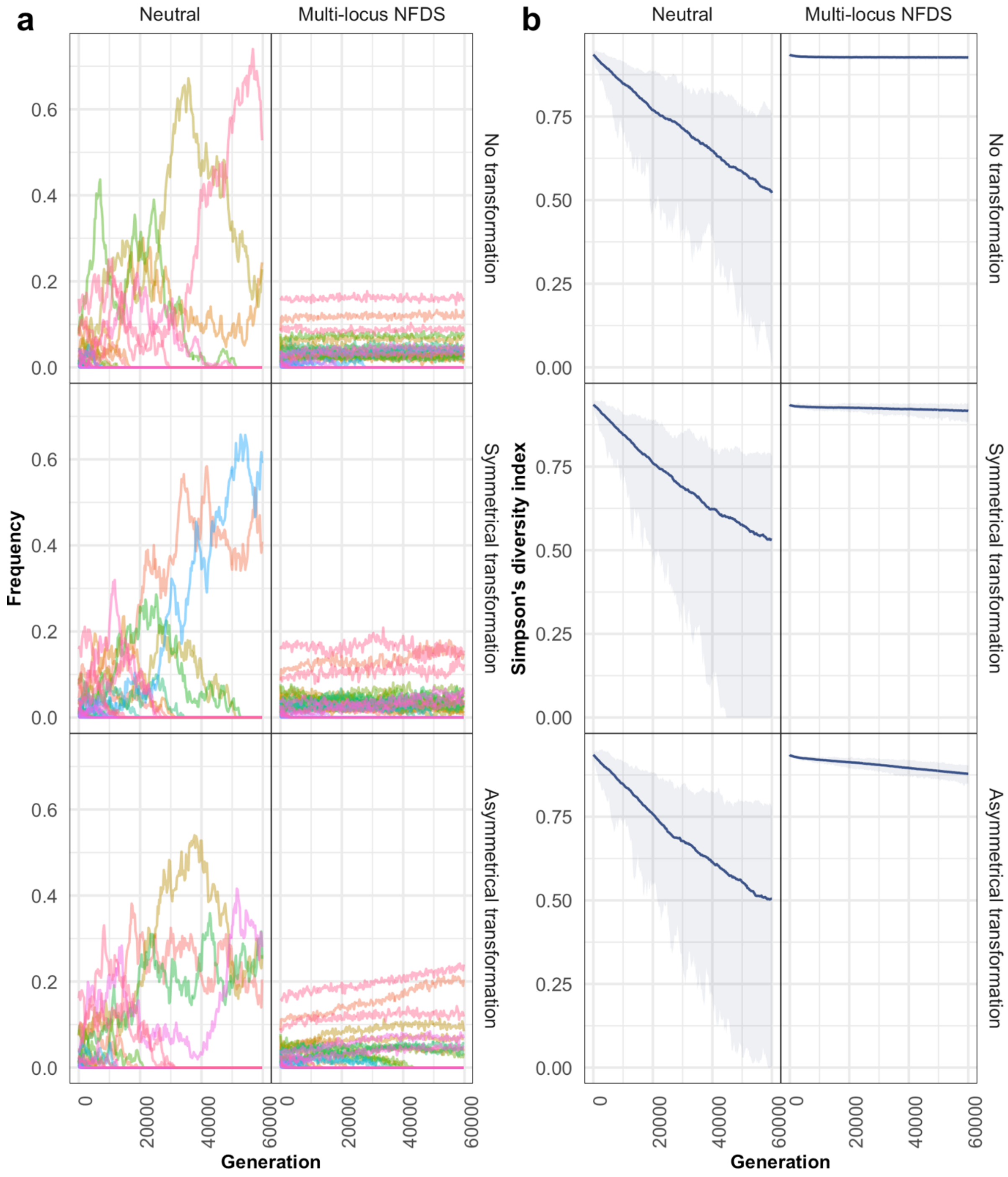

Figure S12: Plots describing the dynamics of simulated populations. Data are displayed as in Fig. S1. These simulations featured weak multi-locus NFDS (Table 1).

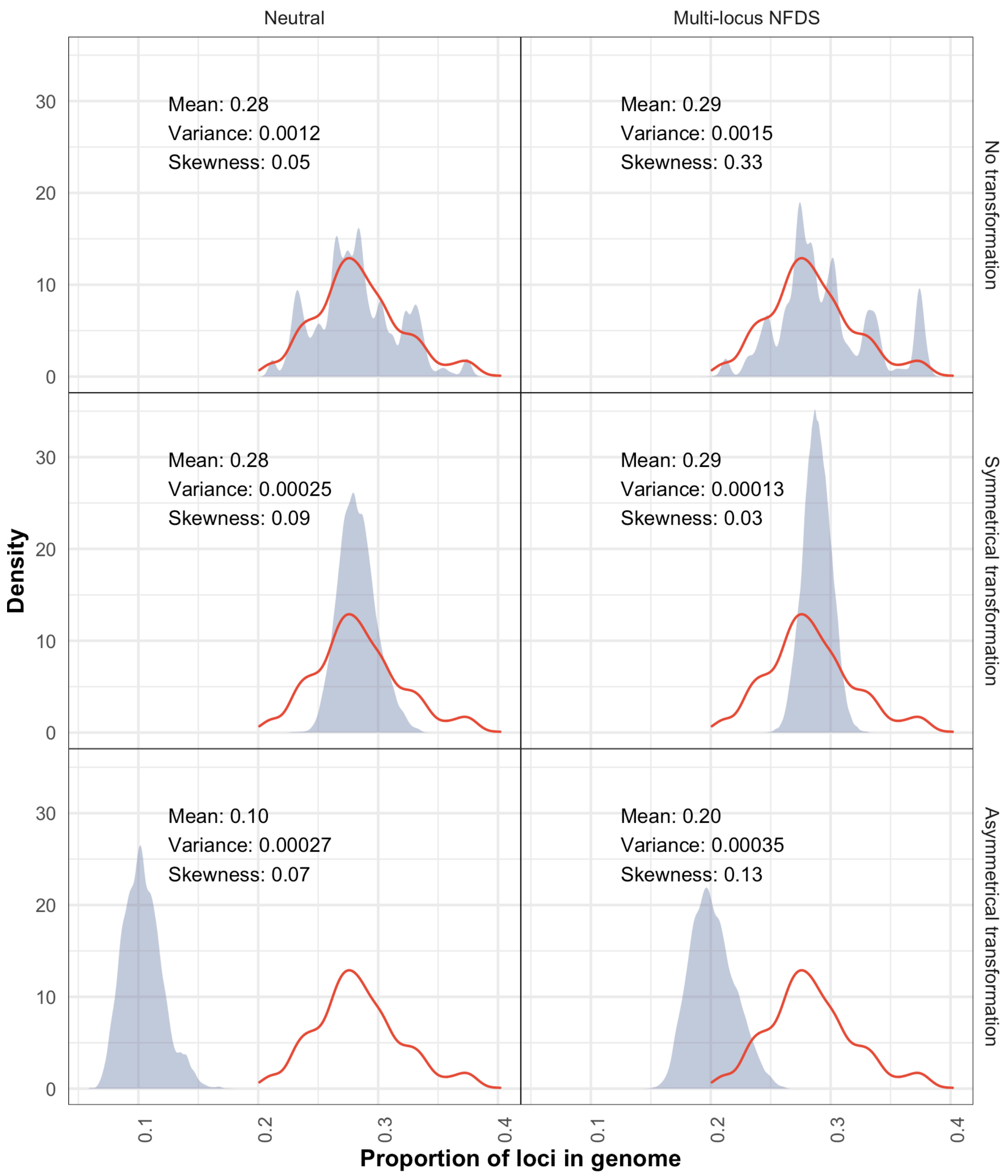

Figure S13: Density plots comparing the distribution of the proportion of intermediate-frequency accessory loci encoded by individual isolates in the genomic data with those from the final timepoint of simulations. Data are displayed as in Fig. 2. The red outline shows the distribution from the 616 genomes in the original dataset (mean: 0.28, variance: 0.00132, skewness: 0.10). These simulations featured weak multi-locus NFDS (Table 1).

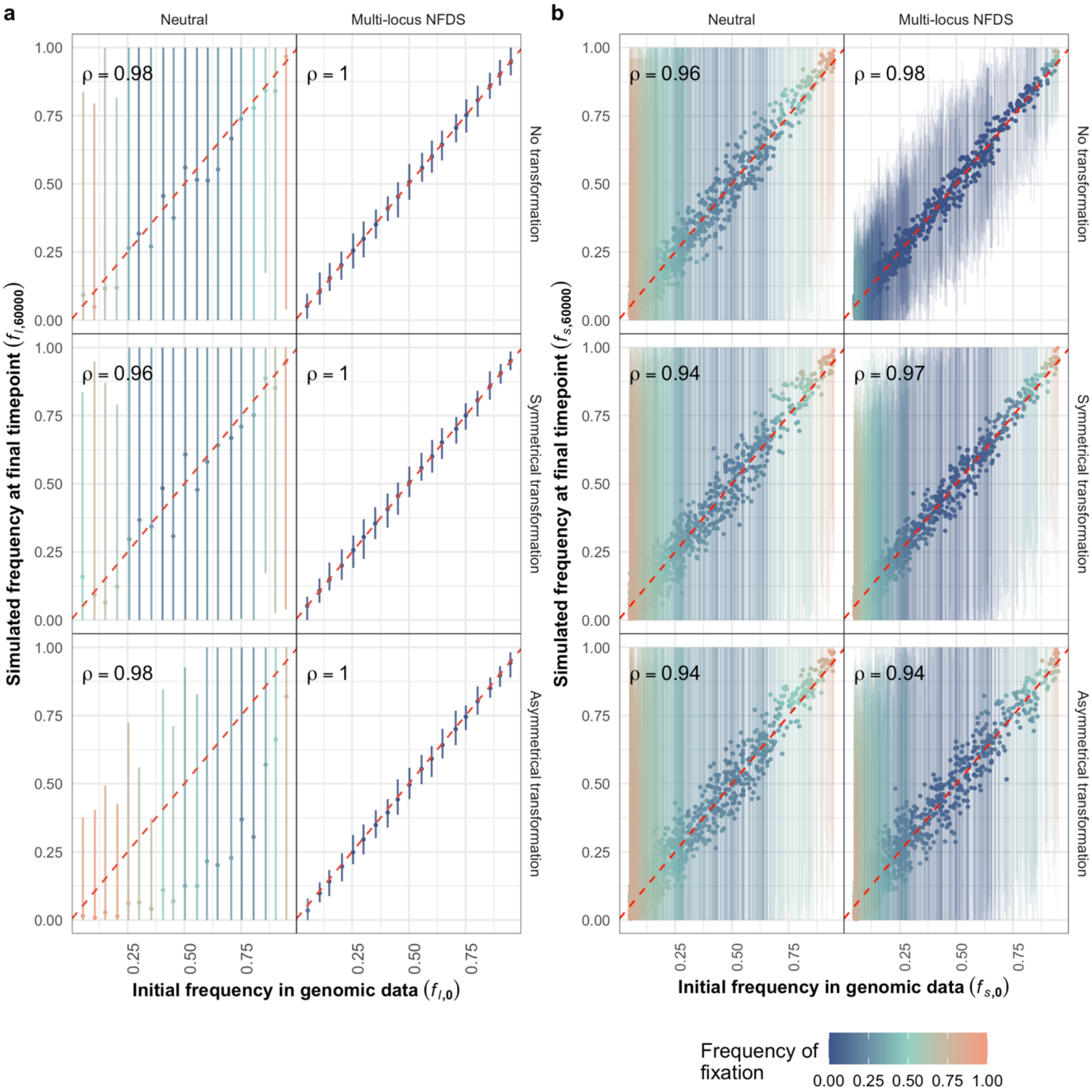

Figure S14: Scatterplots comparing the frequency of alleles at the initial timepoint in the genomic data to their frequency in the final simulation timepoint (N = 616 isolates sampled from each simulation). Data are displayed as in Fig. 1. These simulations included only a reduced subset of ten accessory loci being subject to multi-locus NFDS (Table 1).

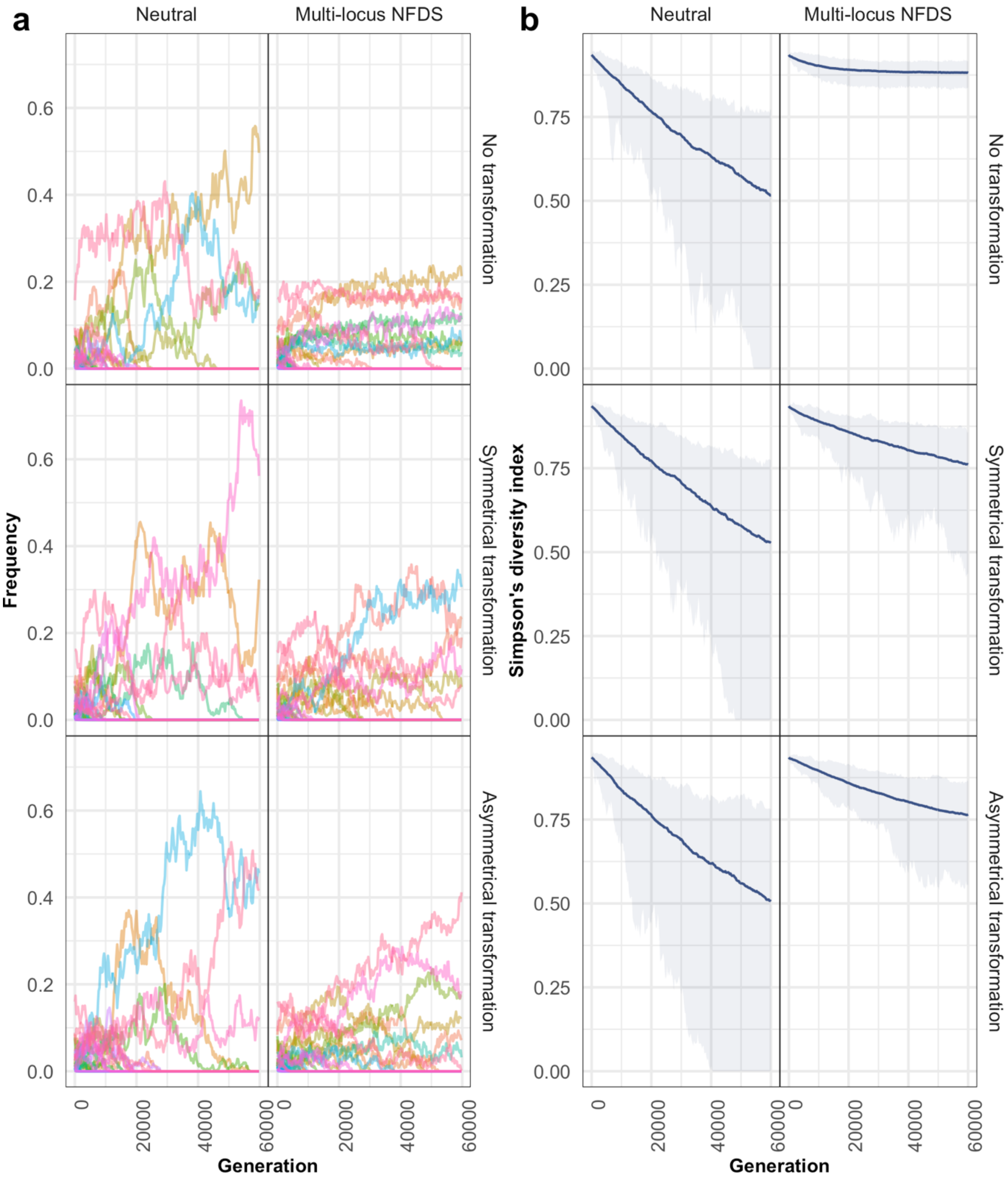

Figure S15: Plots describing the dynamics of simulated populations. Data are displayed as in Fig. S1. These simulations included only a reduced subset of ten accessory loci being subject to multi-locus NFDS (Table 1).

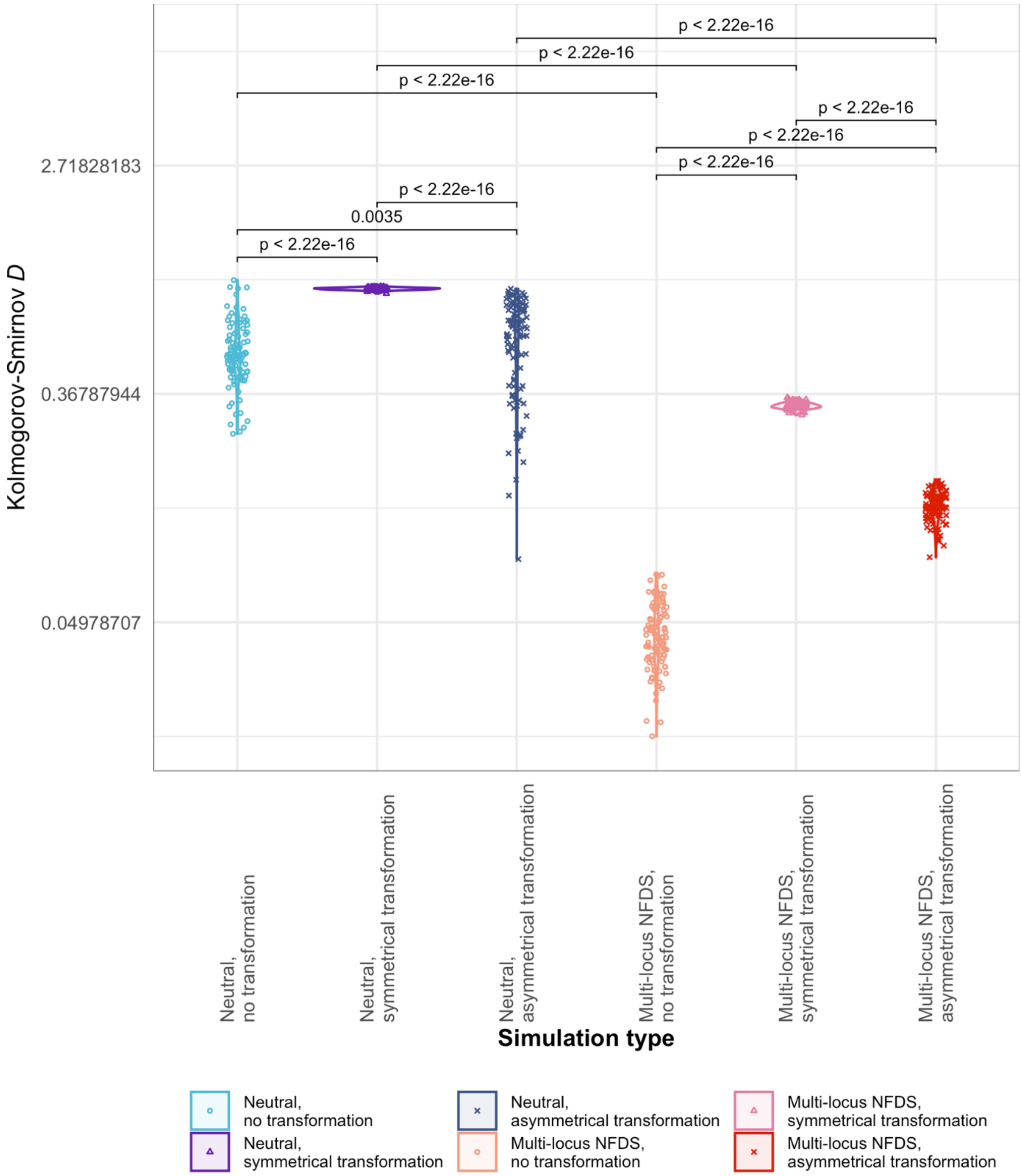

Figure S16: Violin plots comparing the observed distribution of pairwise Jaccard distances, calculated from the accessory loci encoded by genomes, with those from the final timestep of simulations without migration (Fig. 3). Data are shown as in Fig. S2.

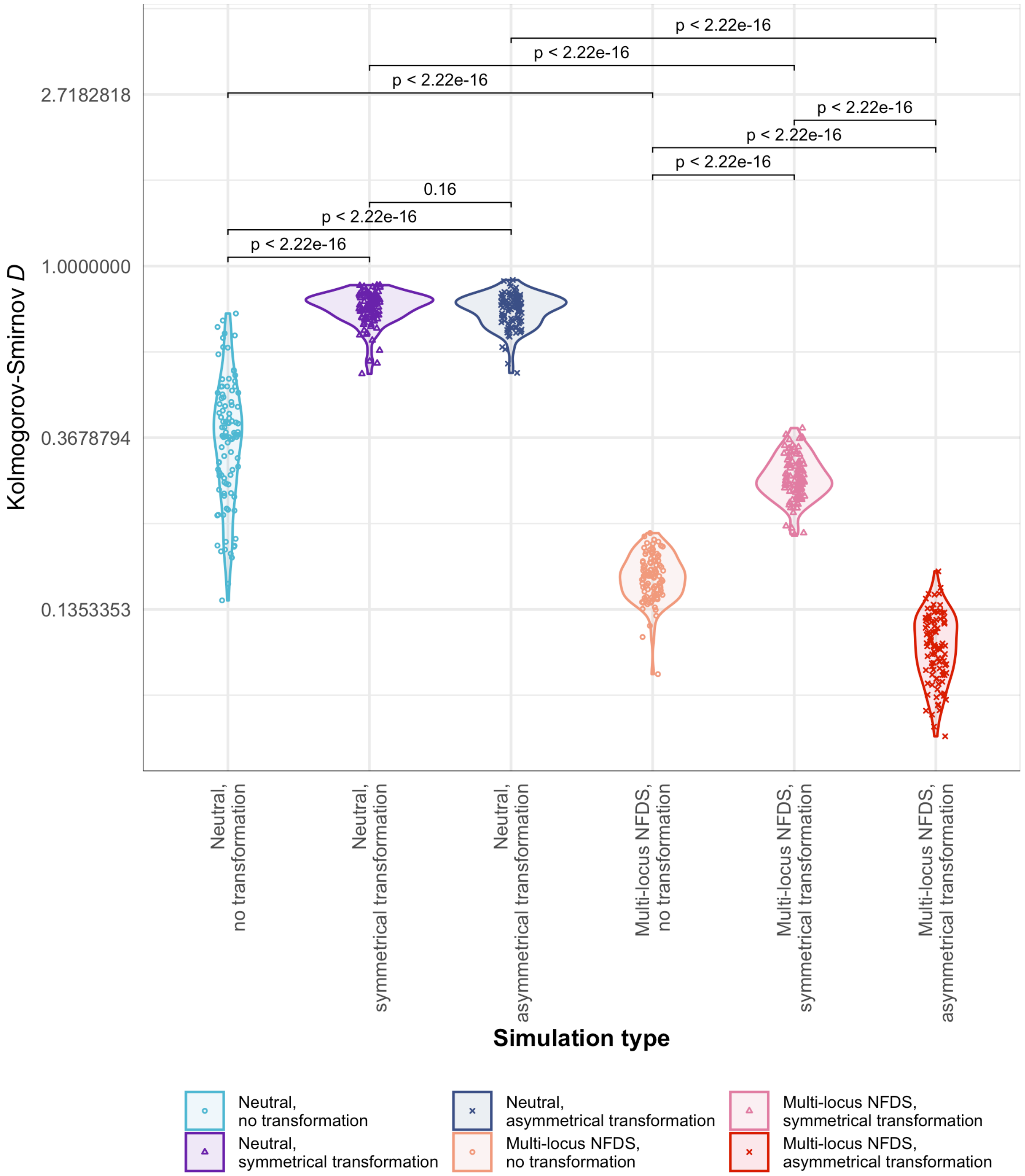

Figure S17: Violin plots comparing the observed distribution of pairwise Hamming distances, calculated from core genome SNPs, to those from the final timesteps of simulations without migration (Fig. 3). Data are shown as in Fig. S2.

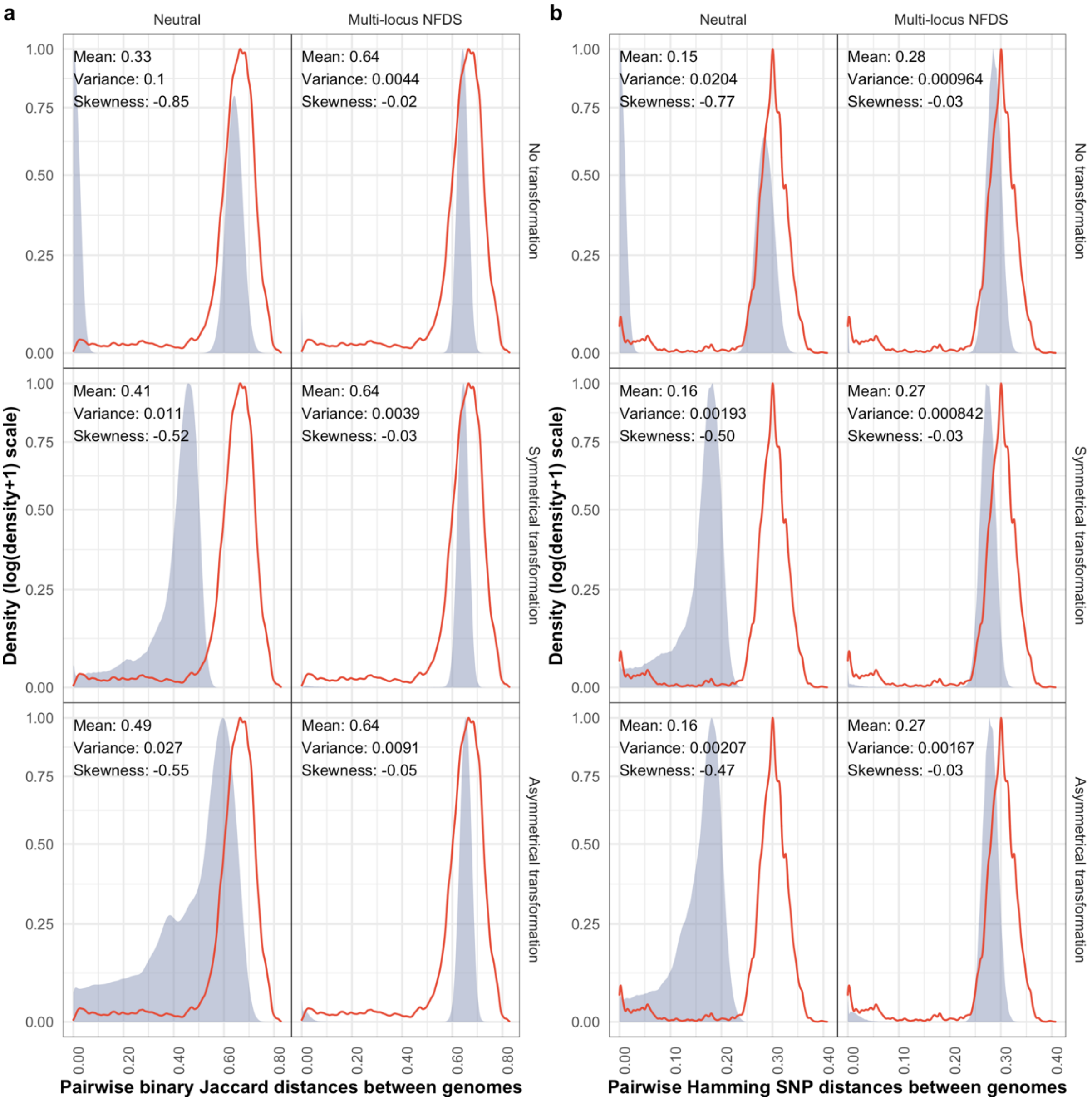

Figure S18: Density plots comparing the distributions of pairwise genetic distances between isolates in the genome data and at the final timepoint of simulations. Data are displayed as in Fig. 3. **a** Pairwise binary Jaccard distances calculated from isolates' accessory loci compared with the distribution from the genomic data (mean: 0.63, variance: 0.015, skewness: -0.22). **b** Pairwise Hamming distances calculated from single nucleotide polymorphisms compared with the distribution from the genomic data (mean: 0.29, variance: 0.0043, skewness: -0.13). These simulations were initiated with populations in which the alleles at each accessory locus, and SNP site, had been permuted across genotypes (Table 1).

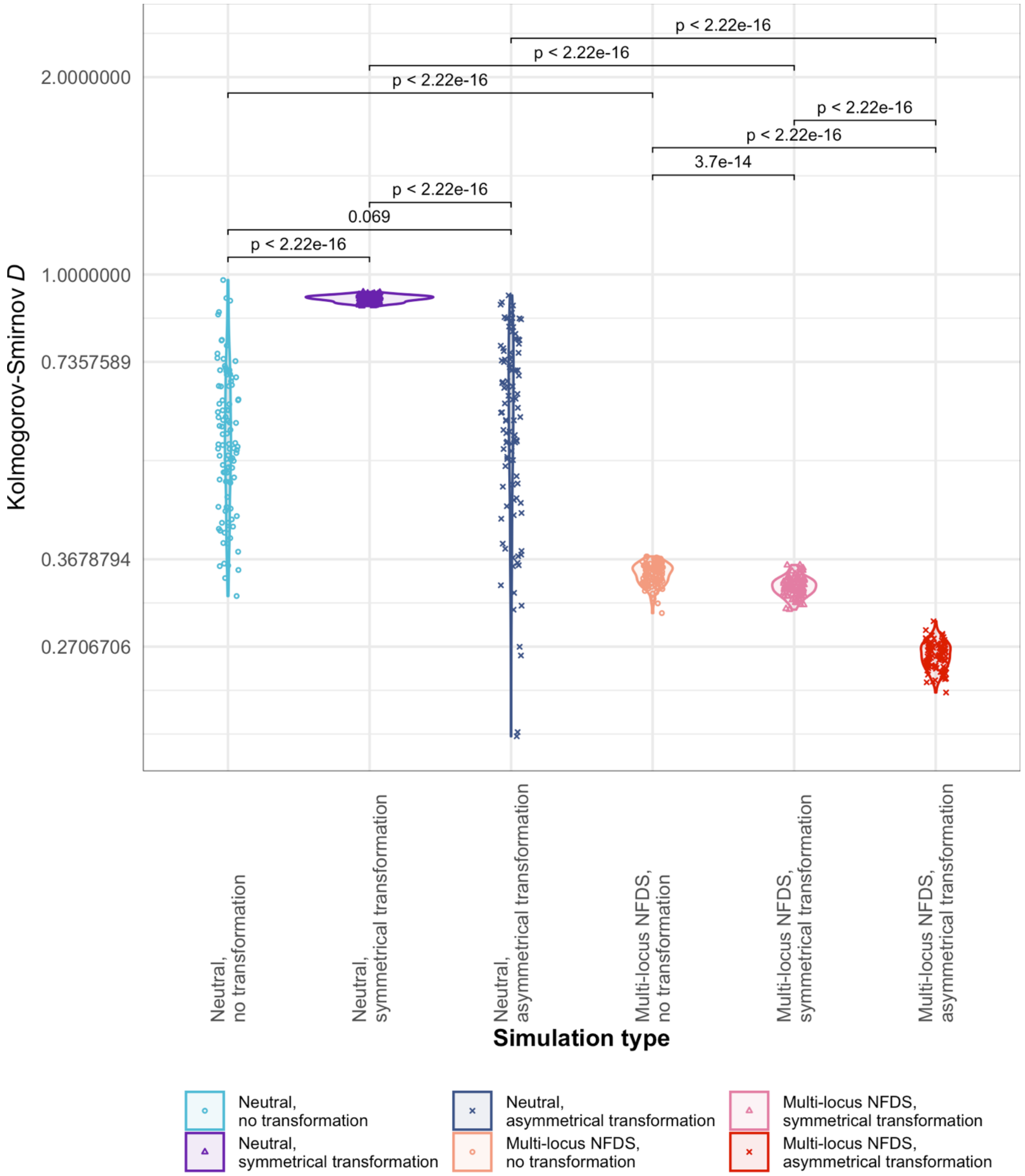

Figure S19: Violin plots comparing the observed distribution of pairwise Jaccard distances, calculated from the accessory loci encoded by genomes, with those from the final timestep of simulations. Data are shown as in Fig. S2. These simulations were initiated with populations in which the alleles at each accessory locus, and SNP site, had been permuted across genotypes (Table 1).

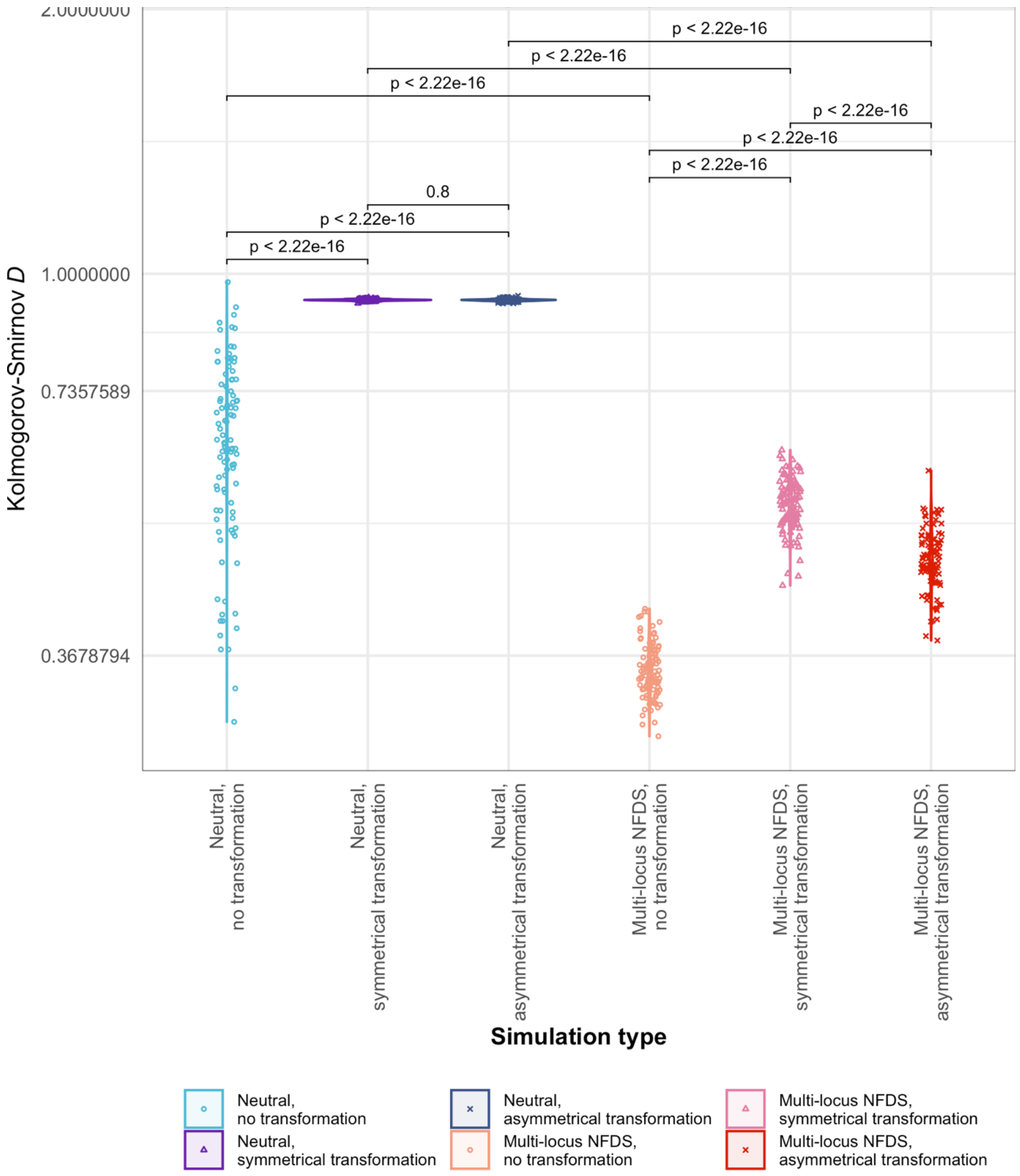

Figure S20: Violin plots comparing the observed distribution of pairwise Hamming distances, calculated from core genome SNPs, to those from the final timesteps of simulations. Data are shown as in Fig. S2. These simulations were initiated with populations in which the alleles at each accessory locus, and SNP site, had been permuted across genotypes (Table 1).

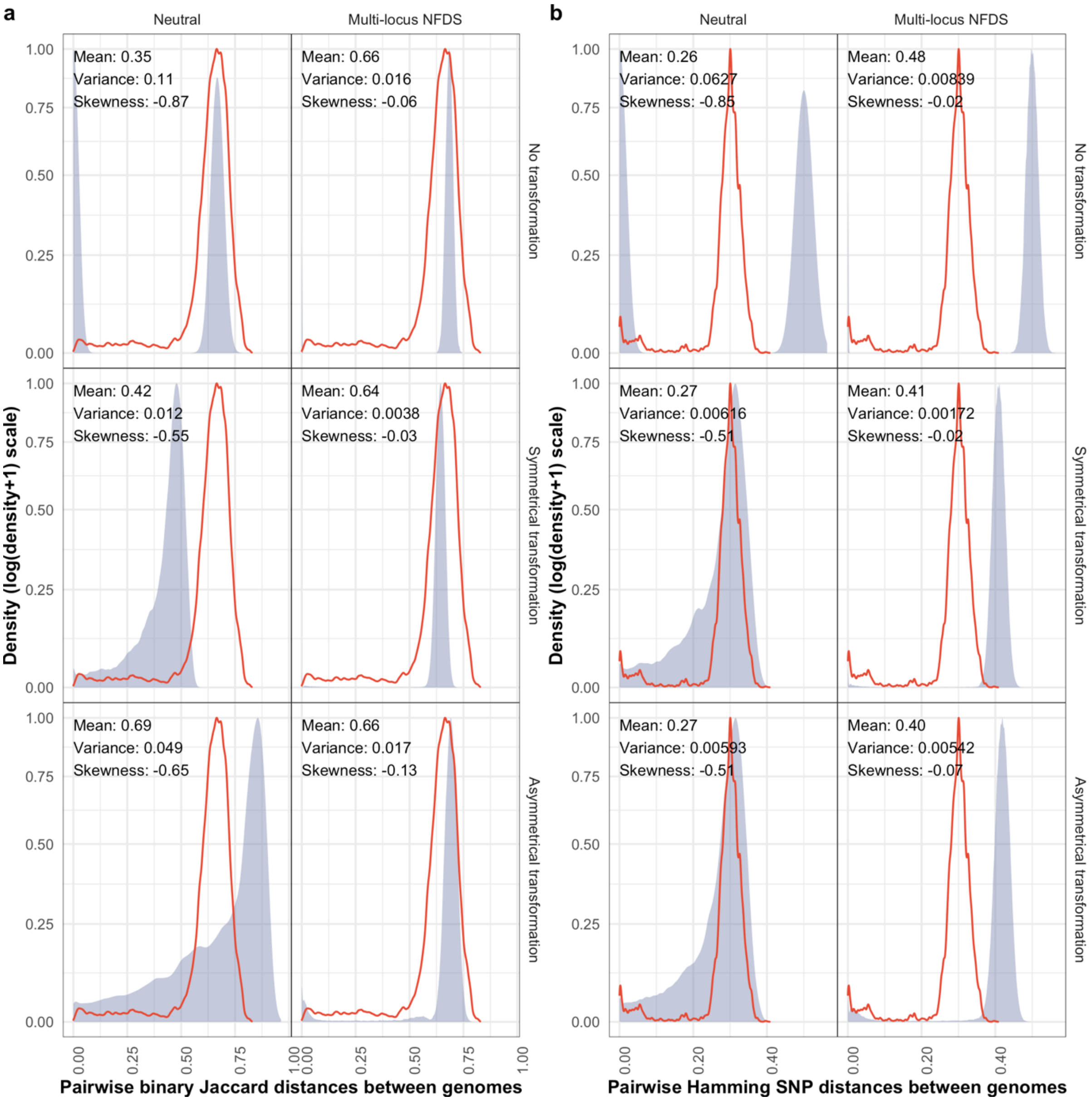

Figure S21: Density plots comparing the distributions of pairwise genetic distances between isolates in the genome data and at the final timepoint of simulations. Data are displayed as in Fig. 3. **a** Pairwise binary Jaccard distances calculated from isolates' accessory loci compared with the distribution from the genomic data (mean: 0.63, variance: 0.015, skewness: -0.22). **b** Pairwise Hamming distances calculated from single nucleotide polymorphisms compared with the distribution from the genomic data (mean: 0.29, variance: 0.0043, skewness: -0.13). These simulations were initiated with populations in which the alleles at each accessory locus, and SNP site, were randomly generated (Table 1).

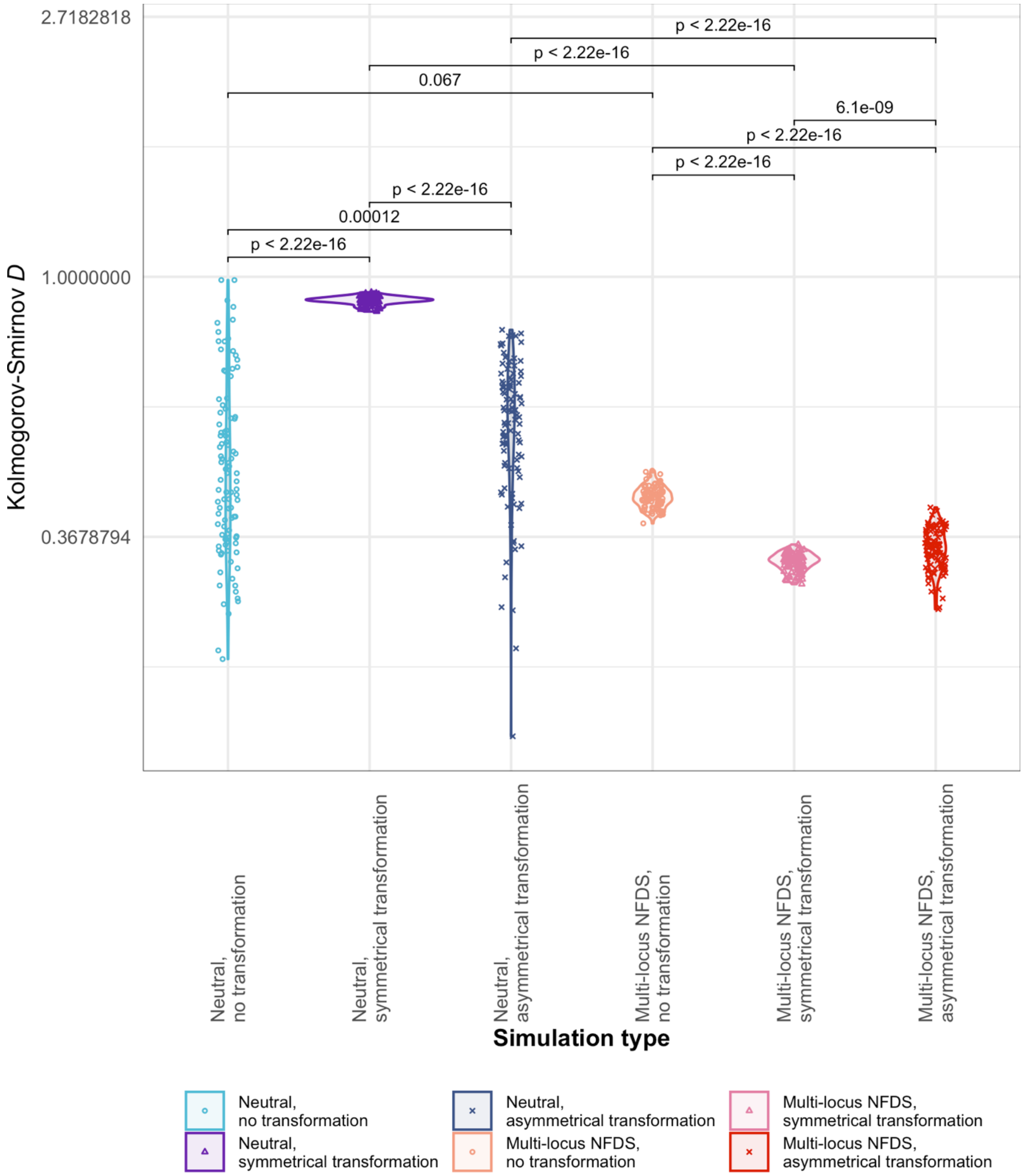

Figure S22: Violin plots comparing the observed distribution of pairwise Jaccard distances, calculated from the accessory loci encoded by genomes, with those from the final timestep of simulations. Data are shown as in Fig. S2. These simulations were initiated with populations in which the alleles at each accessory locus, and SNP site, were randomly generated (Table 1).

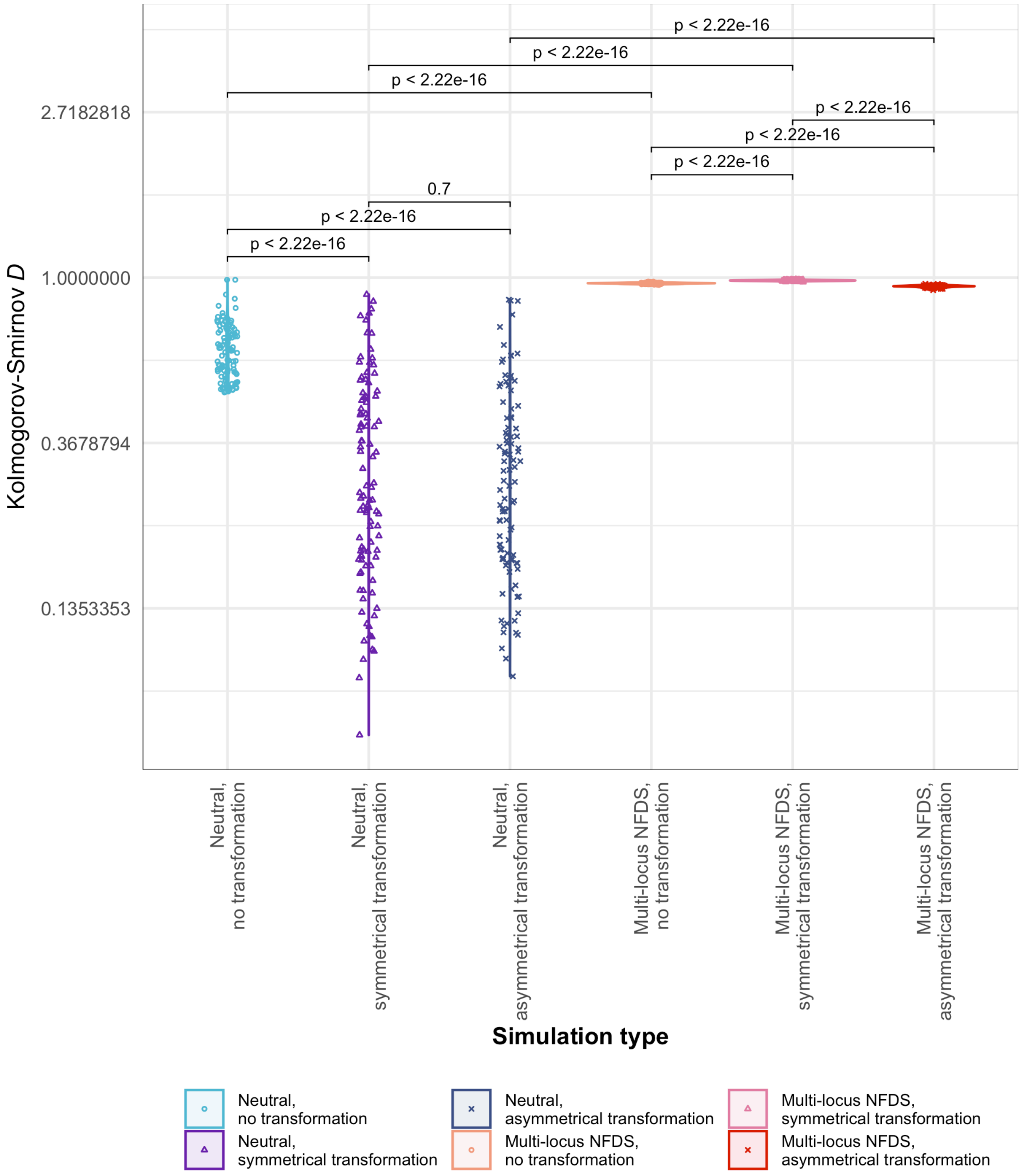

Figure S23: Violin plots comparing the observed distribution of pairwise Hamming distances, calculated from core genome SNPs, to those from the final timesteps of simulations. Data are shown as in Fig. S2. These simulations were initiated with populations in which the alleles at each accessory locus, and SNP site, were randomly generated (Table 1).

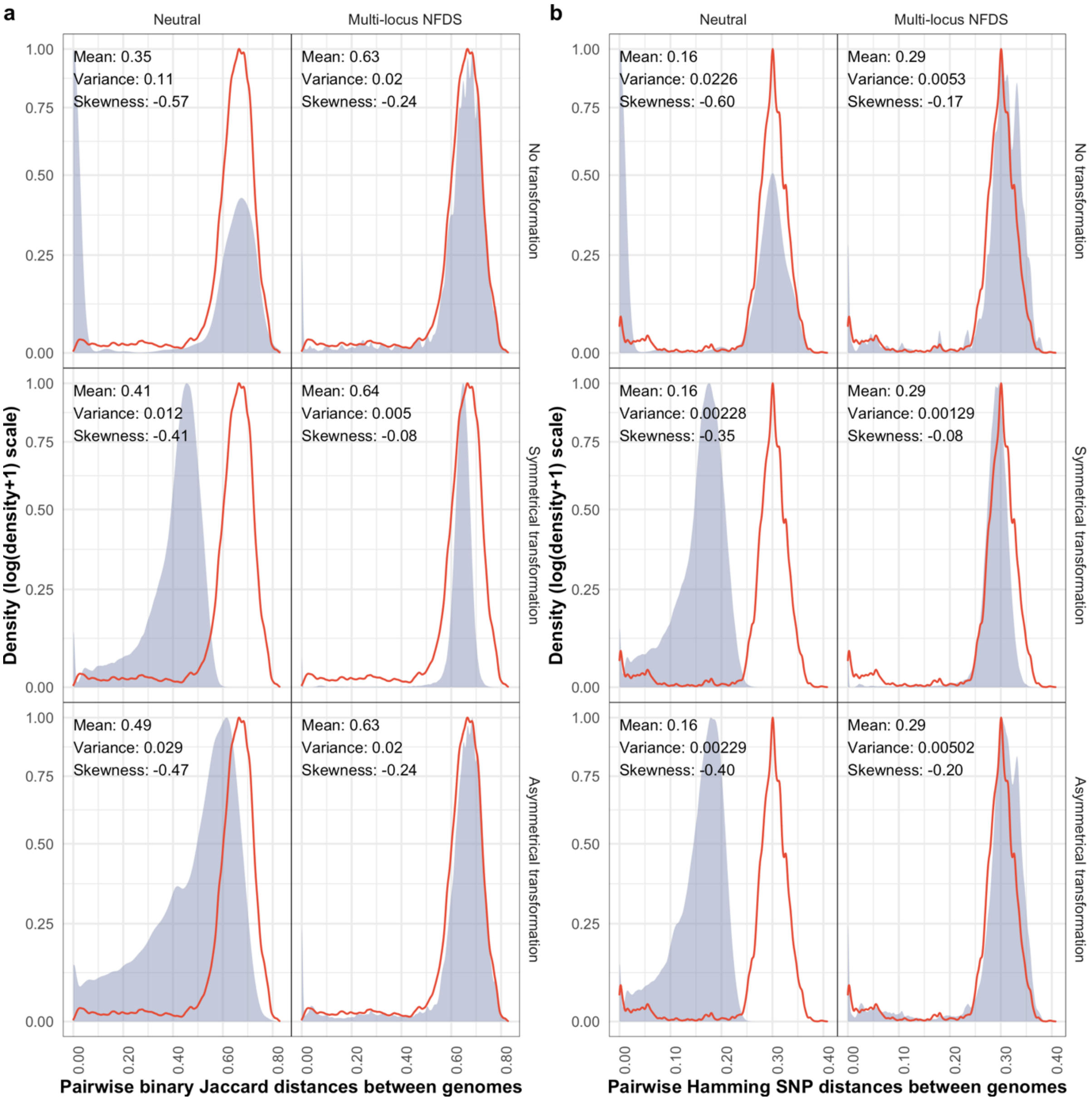

Figure S24: Density plots comparing the distributions of pairwise genetic distances between isolates in the genome data and at the final timepoint of simulations. Data are displayed as in Fig. 3. **a** Pairwise binary Jaccard distances calculated from isolates' accessory loci compared with the distribution from the genomic data (mean: 0.63, variance: 0.015, skewness: -0.22). **b** Pairwise Hamming distances calculated from single nucleotide polymorphisms compared with the distribution from the genomic data (mean: 0.29, variance: 0.0043, skewness: -0.13). These simulations featured saltational transformation (Table 1).

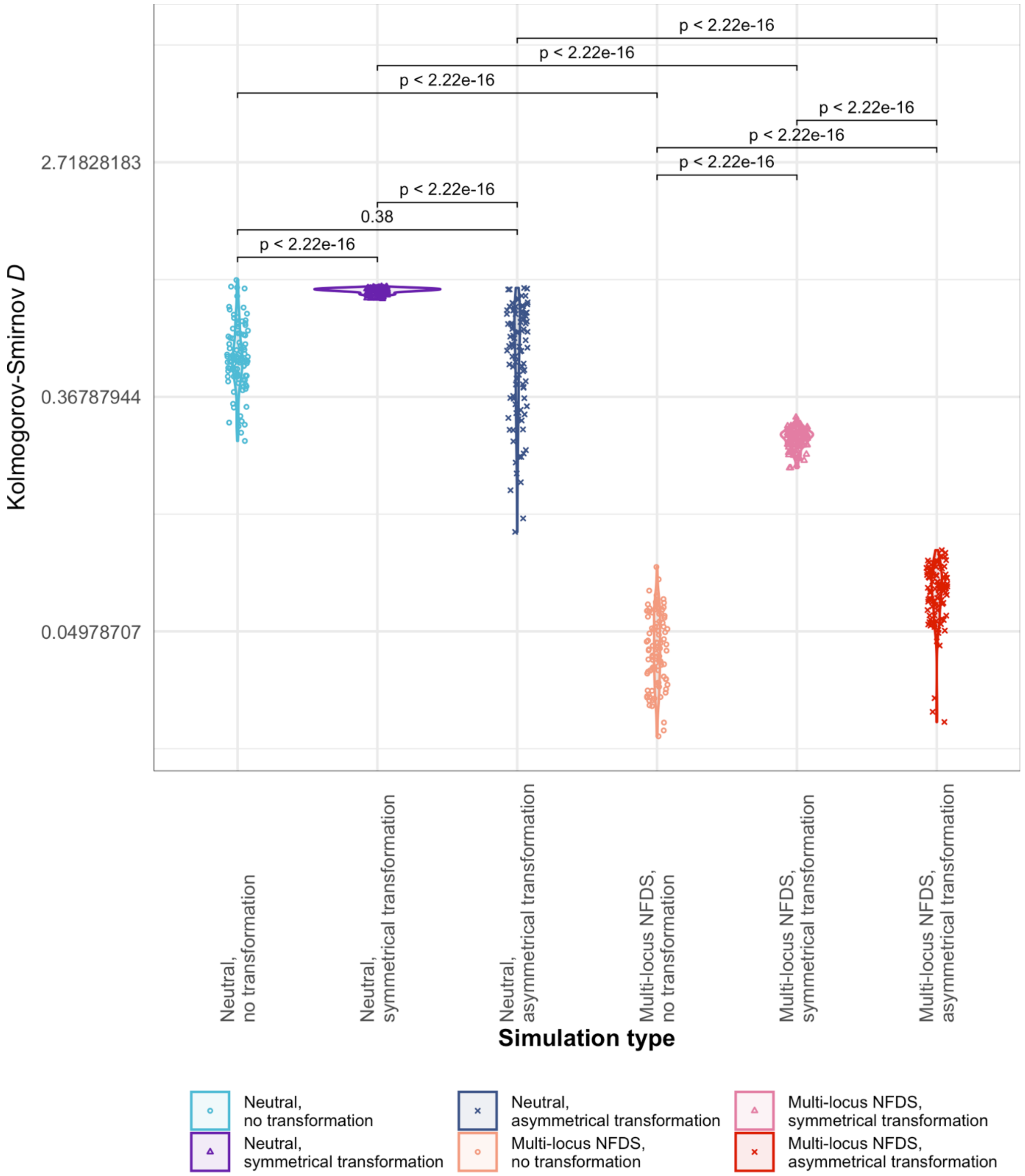

Figure S25: Violin plots comparing the observed distribution of pairwise Jaccard distances, calculated from the accessory loci encoded by genomes, with those from the final timestep of simulations. Data are shown as in Fig. S2. These simulations featured saltational transformation (Table 1).

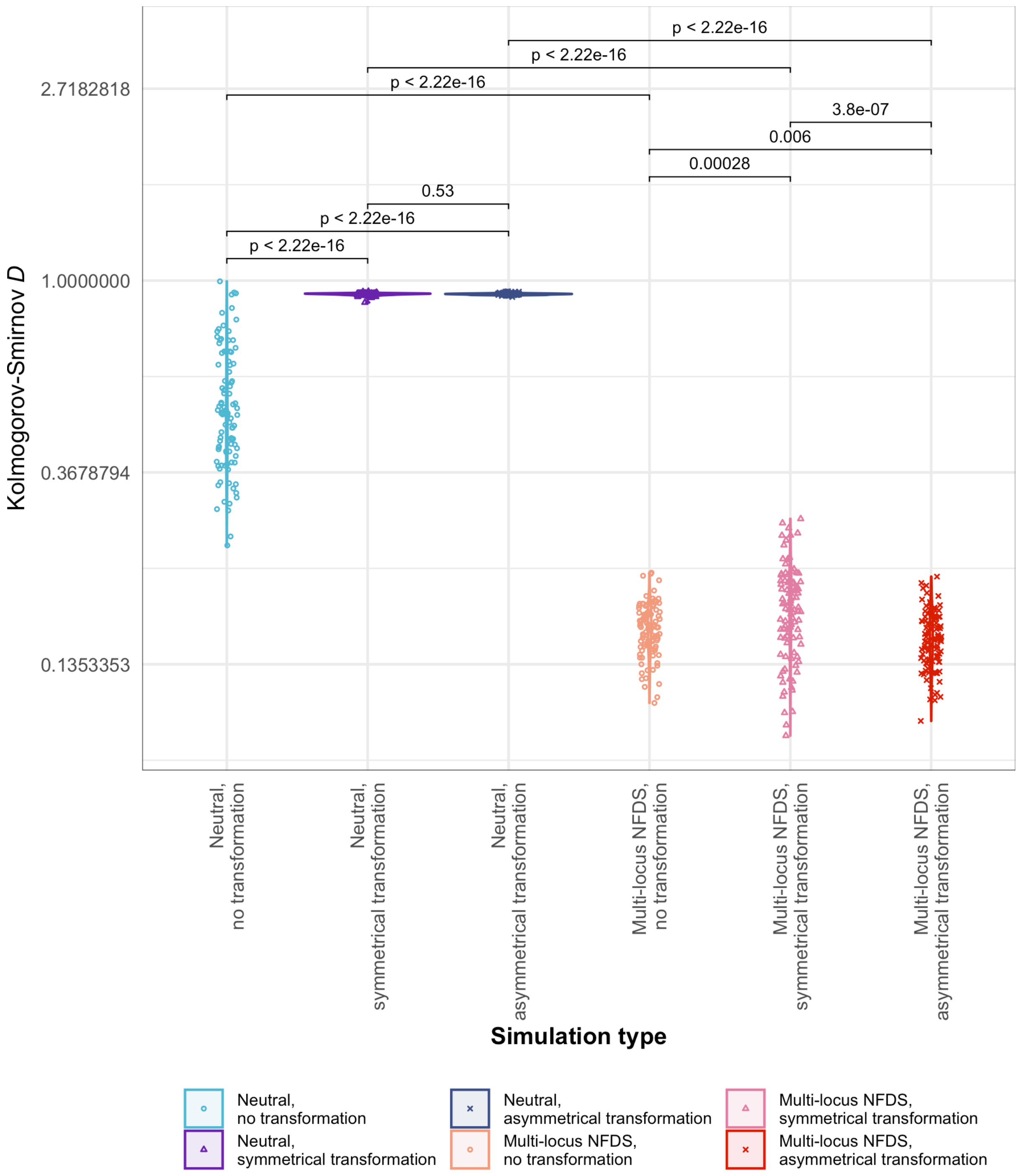

Figure S26: Violin plots comparing the observed distribution of pairwise Hamming distances, calculated from core genome SNPs, to those from the final timesteps of simulations. Data are shown as in Fig. S2. These simulations featured saltational transformation (Table 1).

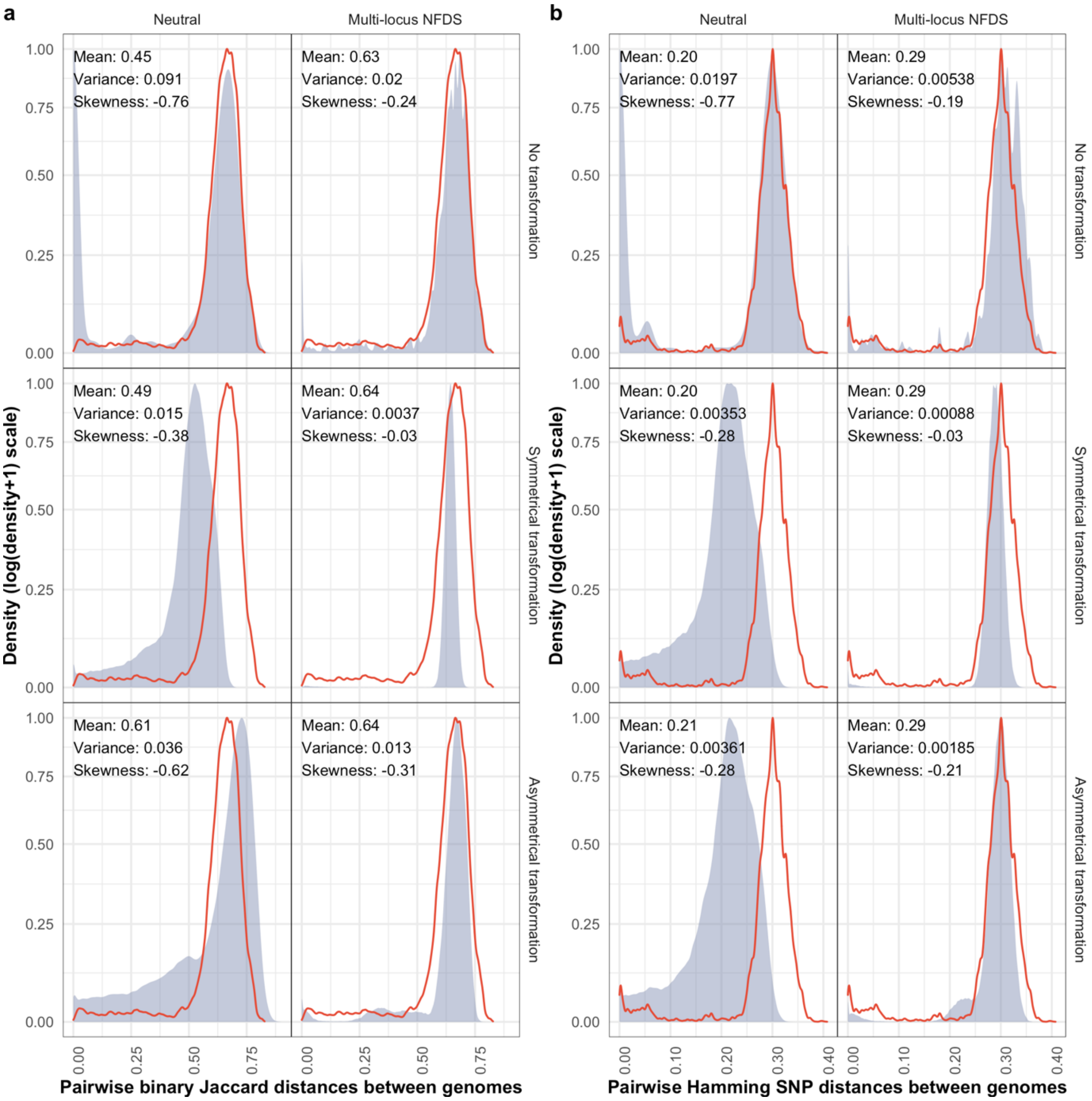

Figure S27: Density plots comparing the distributions of pairwise genetic distances between isolates in the genome data and at the final timepoint of simulations. Data are displayed as in Fig. 3. **a** Pairwise binary Jaccard distances calculated from isolates' accessory loci compared with the distribution from the genomic data (mean: 0.63, variance: 0.015, skewness: -0.22). **b** Pairwise Hamming distances calculated from single nucleotide polymorphisms compared with the distribution from the genomic data (mean: 0.29, variance: 0.0043, skewness: -0.13). These simulations featured inward migration (Table 1).

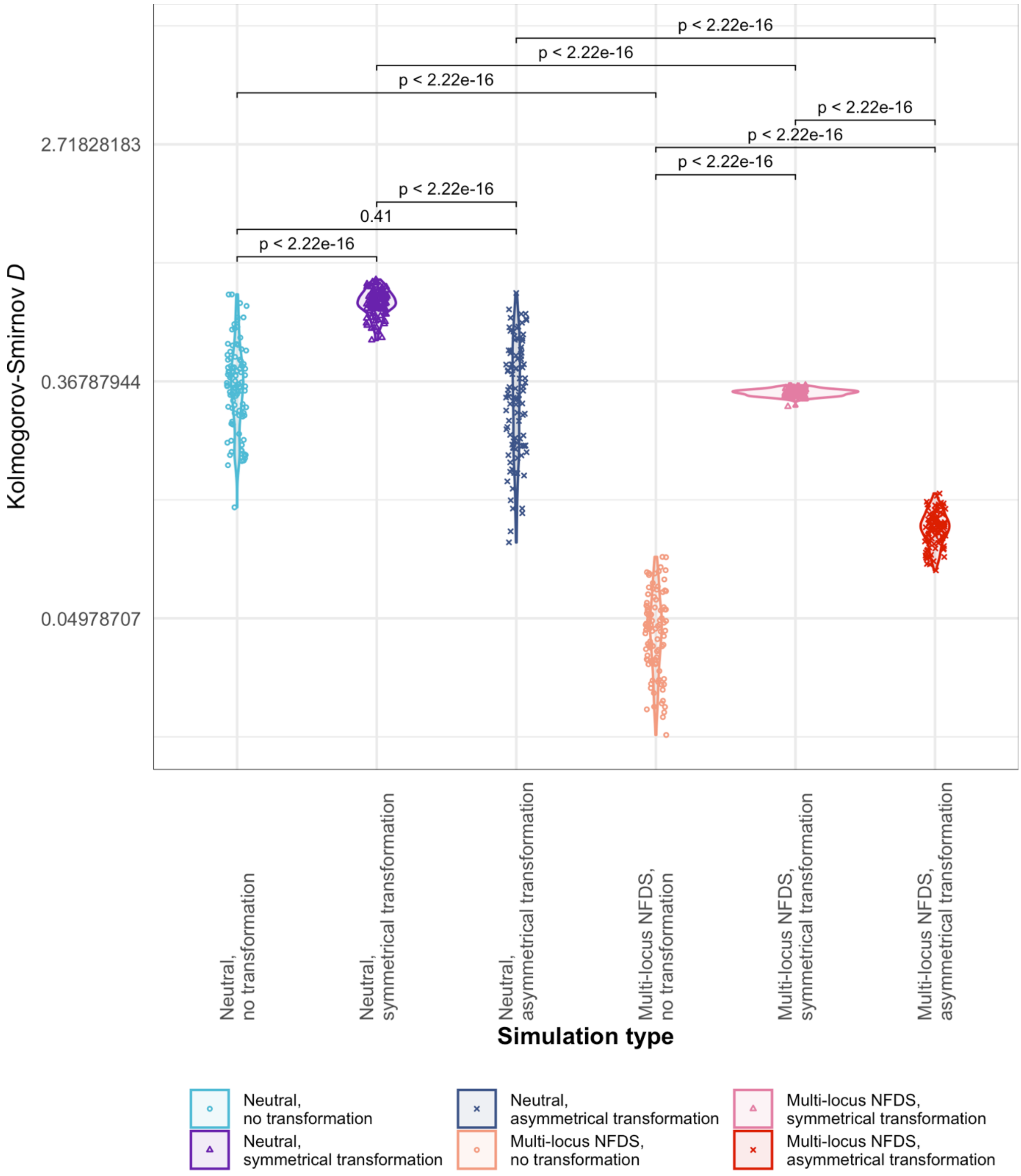

Figure S28: Violin plots comparing the observed distribution of pairwise Jaccard distances, calculated from the accessory loci encoded by genomes, with those from the final timestep of simulations. Data are shown as in Fig. S2. These simulations featured inward migration (Table 1).

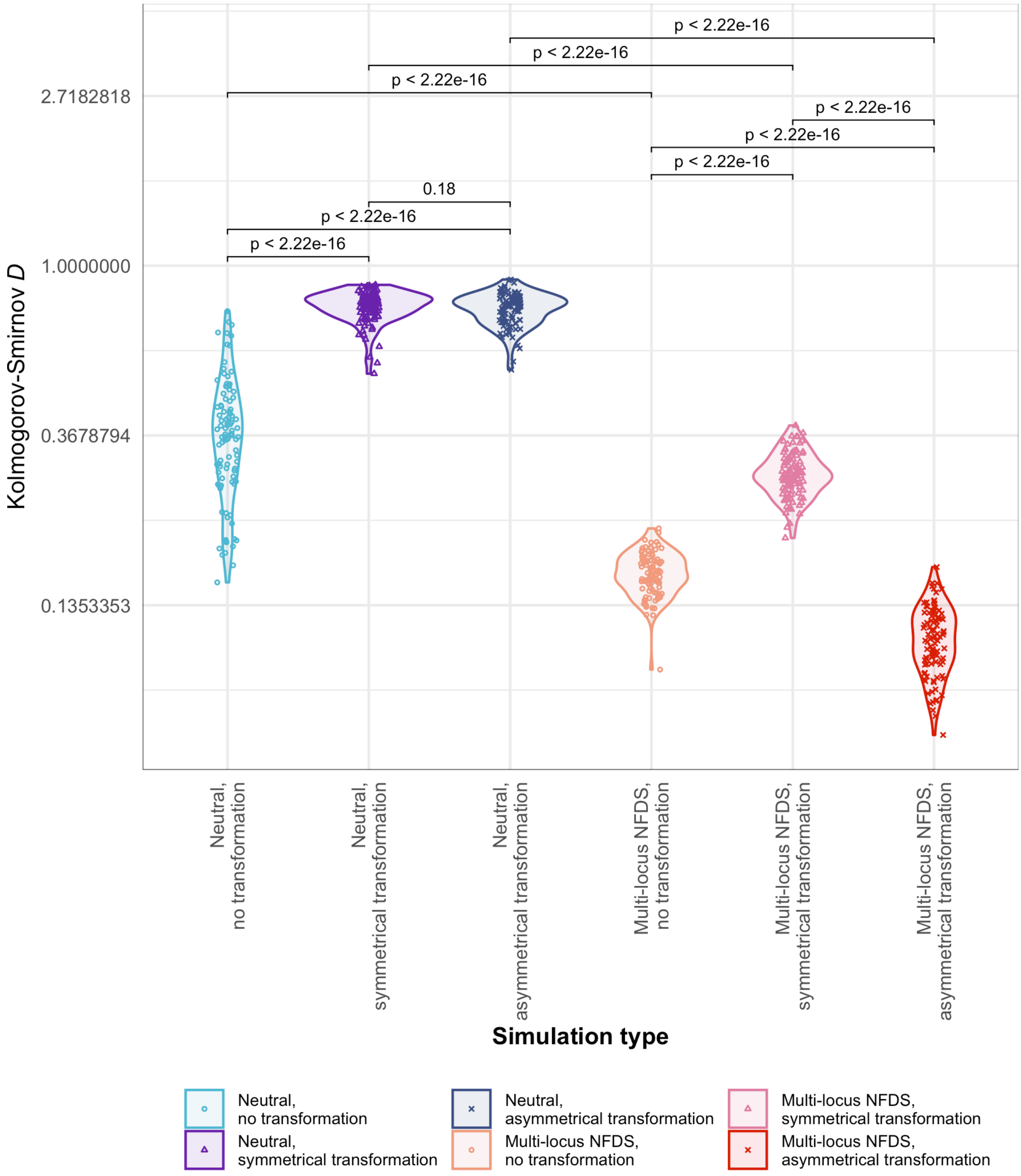

Figure S29: Violin plots comparing the observed distribution of pairwise Hamming distances, calculated from core genome SNPs, to those from the final timesteps of simulations. Data are shown as in Fig. S2. These simulations featured inward migration (Table 1).

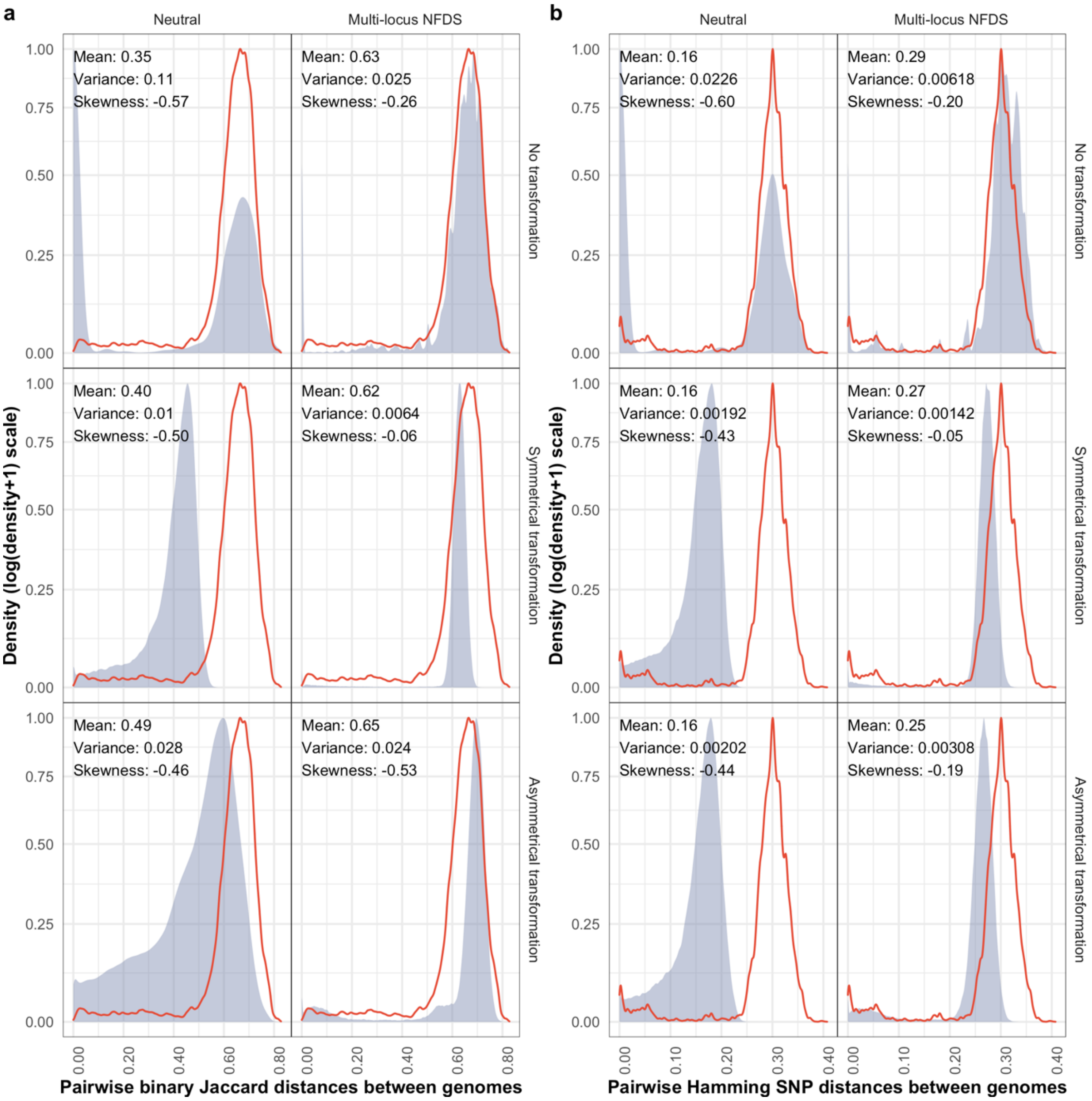

Figure S30: Density plots comparing the distributions of pairwise genetic distances between isolates in the genome data and at the final timepoint of simulations. Data are displayed as in Fig. 3. **a** Pairwise binary Jaccard distances calculated from isolates' accessory loci compared with the distribution from the genomic data (mean: 0.63, variance: 0.015, skewness: -0.22). **b** Pairwise Hamming distances calculated from single nucleotide polymorphisms compared with the distribution from the genomic data (mean: 0.29, variance: 0.0043, skewness: -0.13). These simulations featured weak multi-locus NFDS (Table 1).

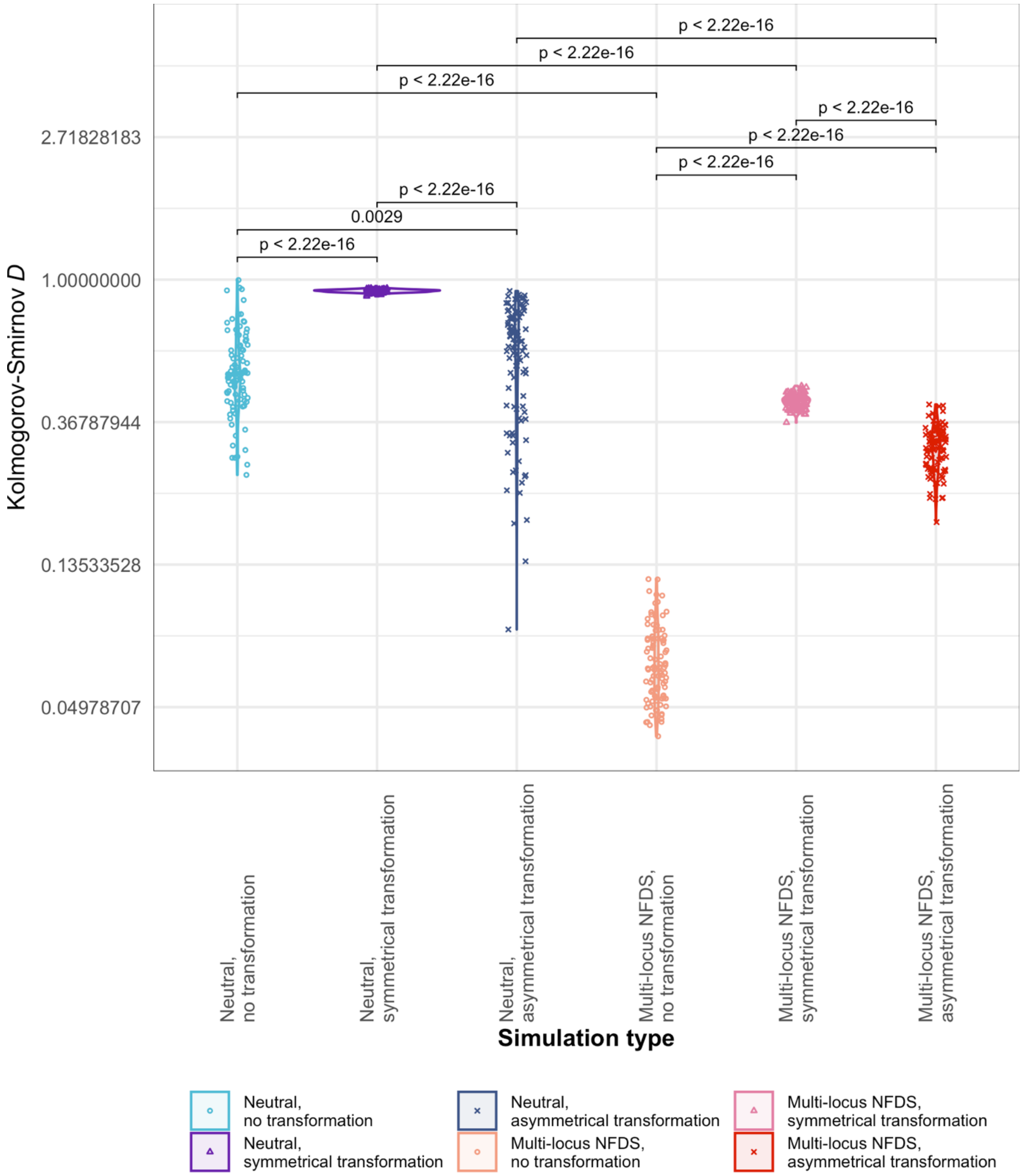

Figure S31: Violin plots comparing the observed distribution of pairwise Jaccard distances, calculated from the accessory loci encoded by genomes, with those from the final timestep of simulations. Data are shown as in Fig. S2. These simulations featured weak multi-locus NFDS (Table 1).

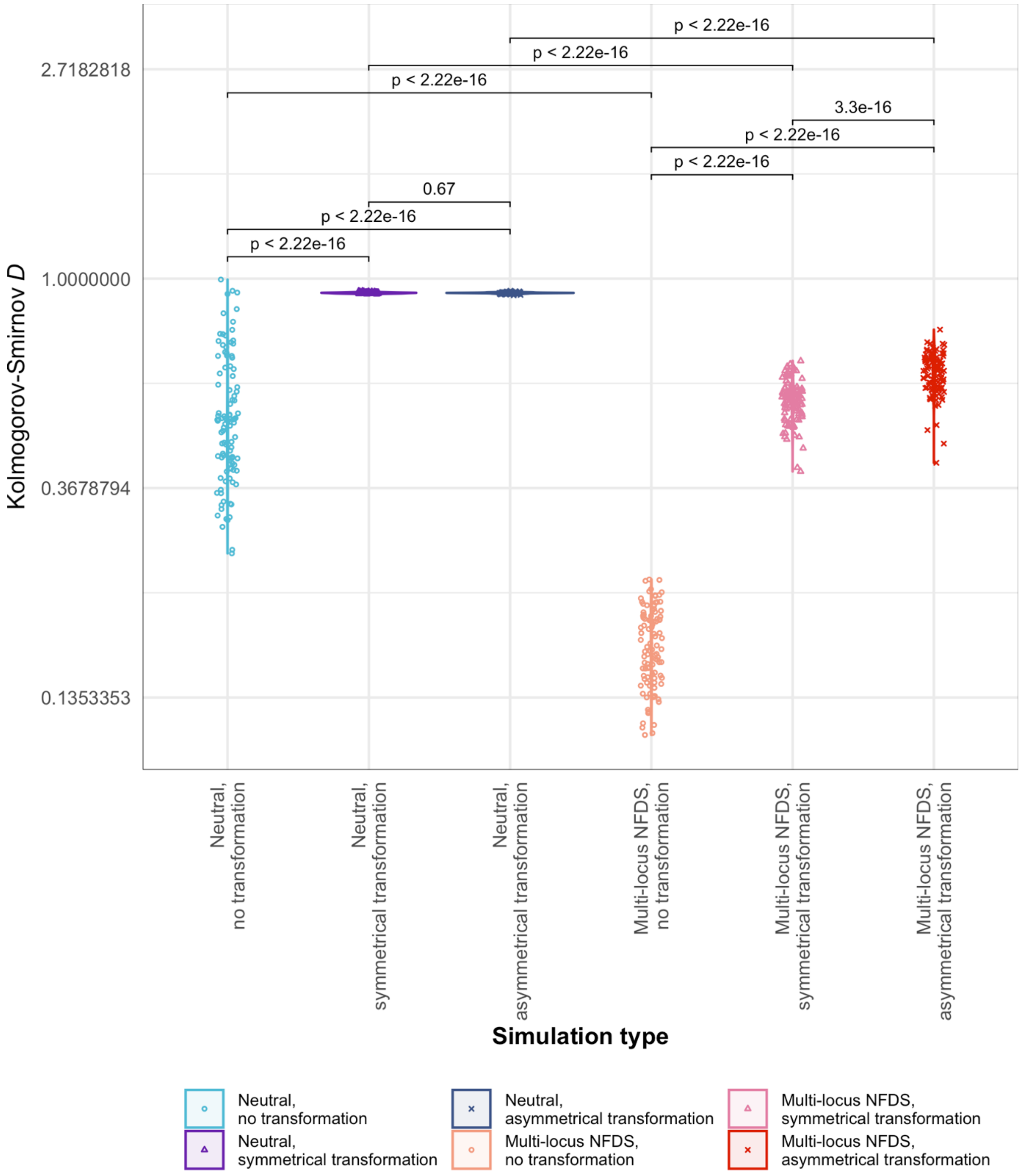

Figure S32: Violin plots comparing the observed distribution of pairwise Hamming distances, calculated from core genome SNPs, to those from the final timesteps of simulations. Data are shown as in Fig. S2. These simulations featured weak multi-locus NFDS (Table 1).

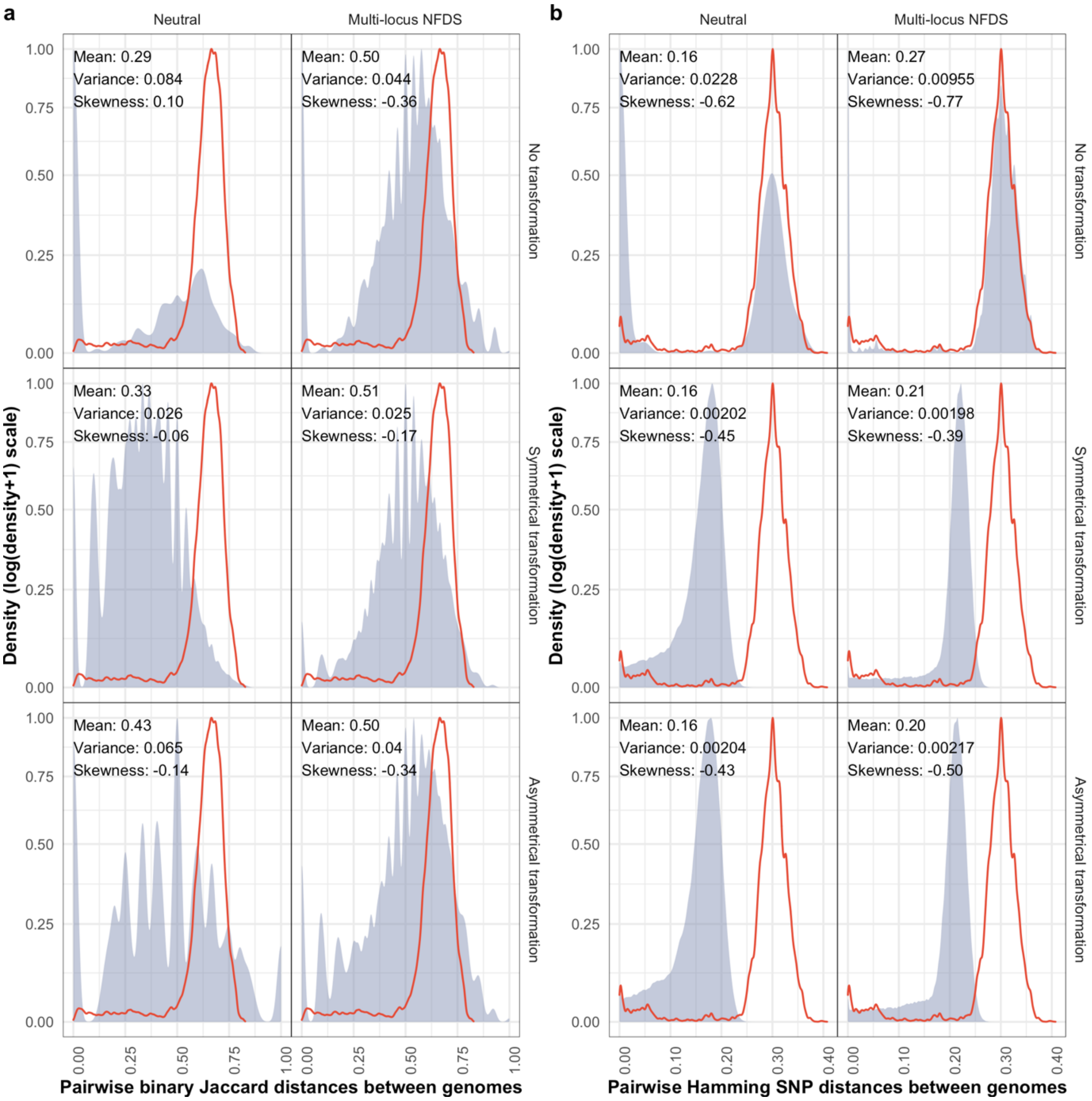

Figure S33: Density plots comparing the distributions of pairwise genetic distances between isolates in the genome data and at the final timepoint of simulations. Data are displayed as in Fig. 3. **a** Pairwise binary Jaccard distances calculated from isolates' accessory loci compared with the distribution from the genomic data (mean: 0.63, variance: 0.015, skewness: -0.22). **b** Pairwise Hamming distances calculated from single nucleotide polymorphisms compared with the distribution from the genomic data (mean: 0.29, variance: 0.0043, skewness: -0.13). These simulations included only a reduced subset of ten accessory loci being subject to multi-locus NFDS (Table 1).

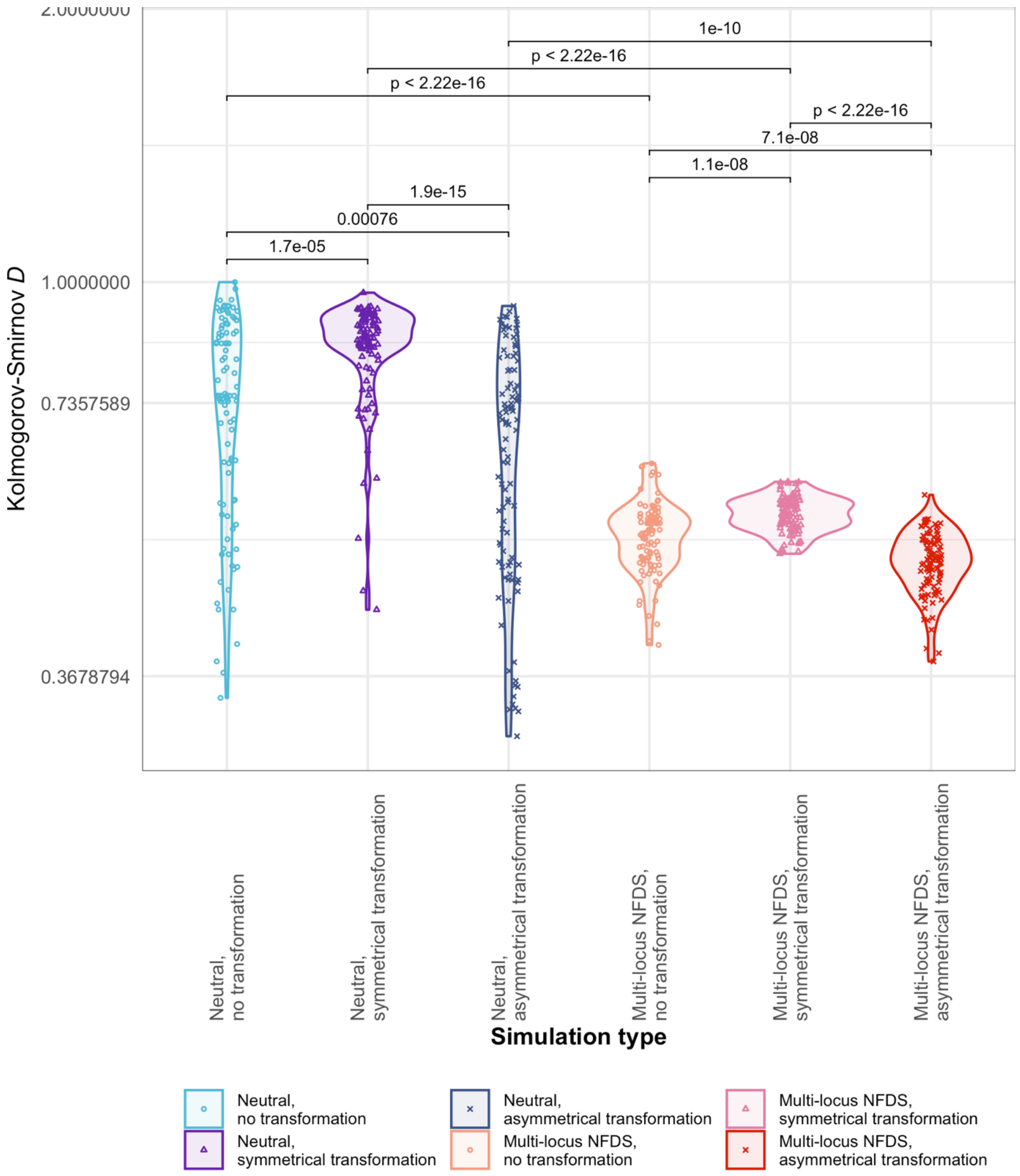

Figure S34: Violin plots comparing the observed distribution of pairwise Jaccard distances, calculated from the accessory loci encoded by genomes, with those from the final timestep of simulations. Data are shown as in Fig. S2. These simulations included only a reduced subset of ten accessory loci being subject to multi-locus NFDS (Table 1).

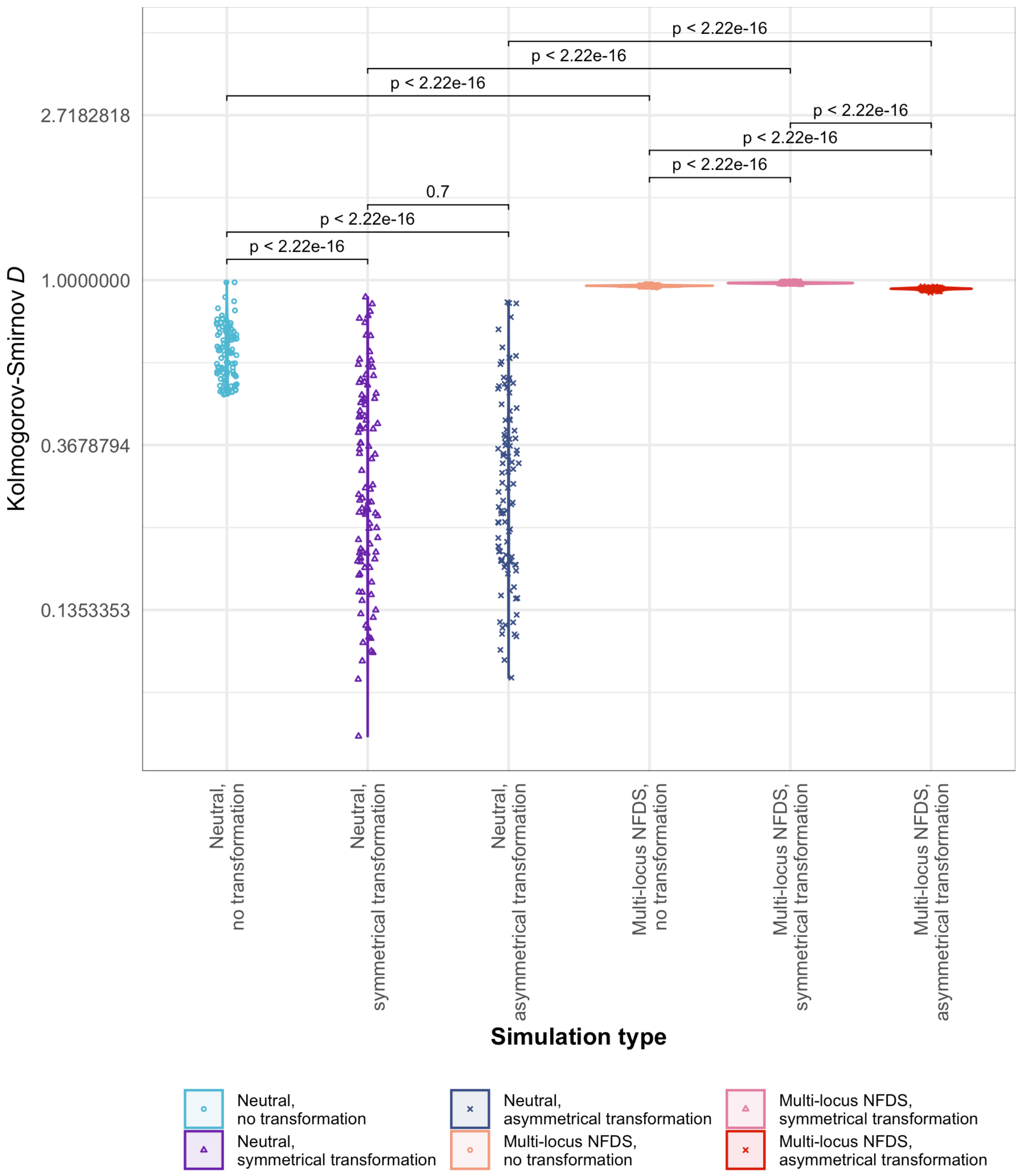

Figure S35: Violin plots comparing the observed distribution of pairwise Hamming distances, calculated from core genome SNPs, to those from the final timesteps of simulations. Data are shown as in Fig. S2. These simulations included only a reduced subset of ten accessory loci being subject to multi-locus NFDS (Table 1).

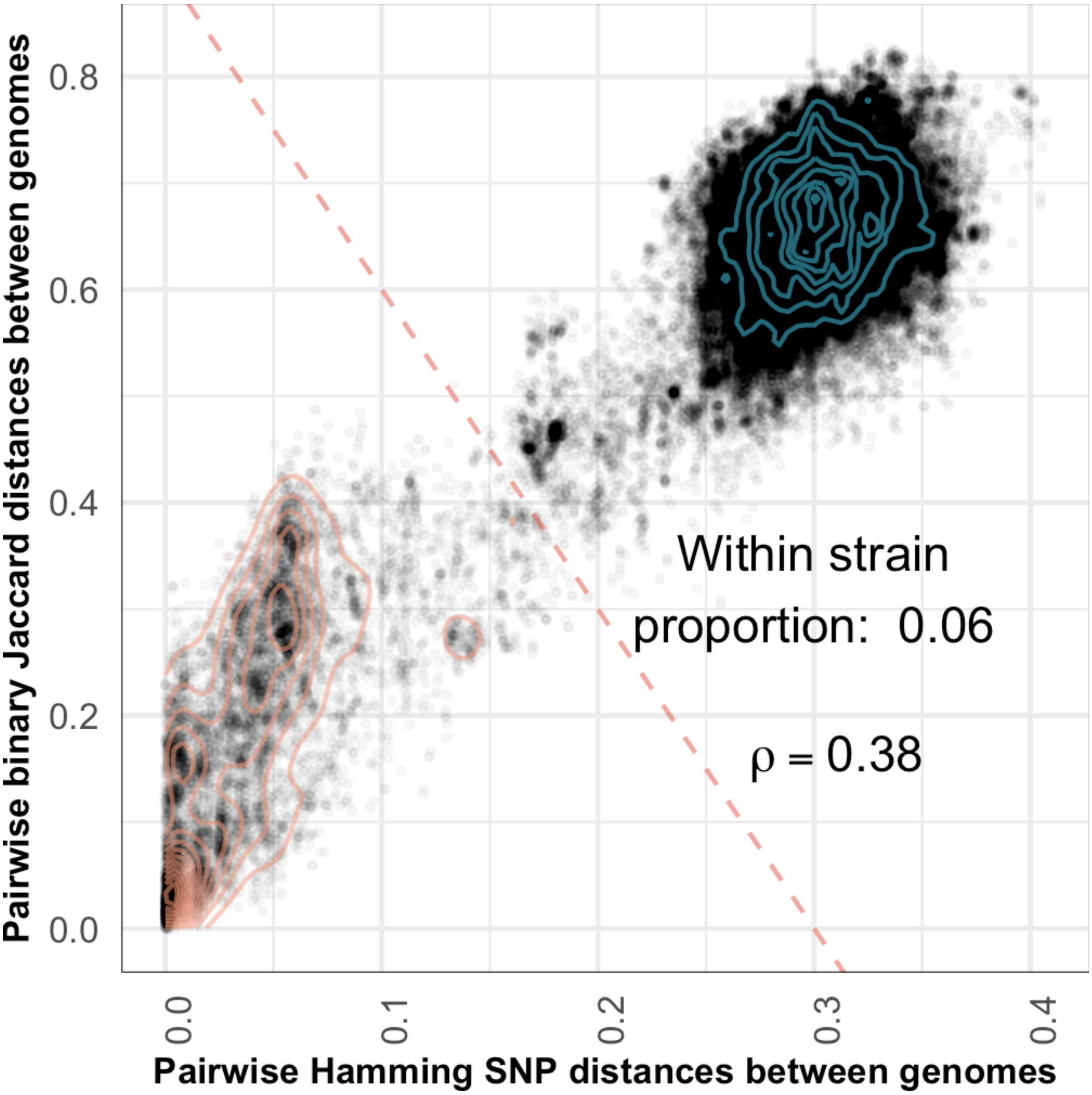

Figure S36: Scatterplot showing the genetic distances (N = 189,420) between isolates in the genomic data, with the horizontal axis representing divergence in core genome single nucleotide polymorphisms (S = 1090), and the vertical axis representing divergence in accessory loci (L = 1090). The red diagonal is a threshold distinguishing within and between strain distances, the densities of which are summarised by the isocontours (orange and blue lines, respectively). The proportion of points classified as comparing isolates of the same strain is annotated on the plot, as is Spearman's ρ.

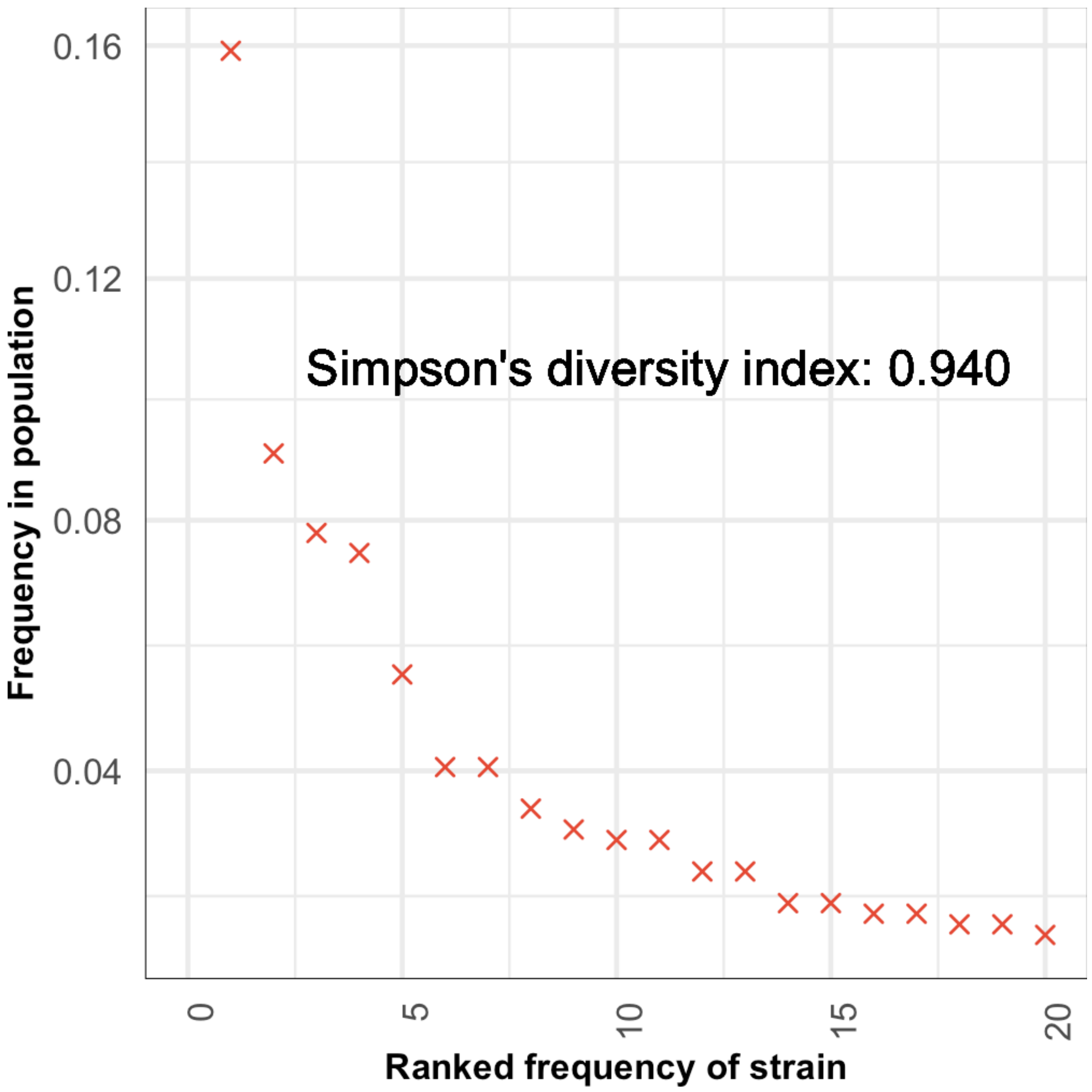

Figure S37: Scatterplot showing the rank-frequency distribution of strains in the overall set of 616 genomes as red crosses. The Simpson's diversity index of the strains is displayed on the plot.

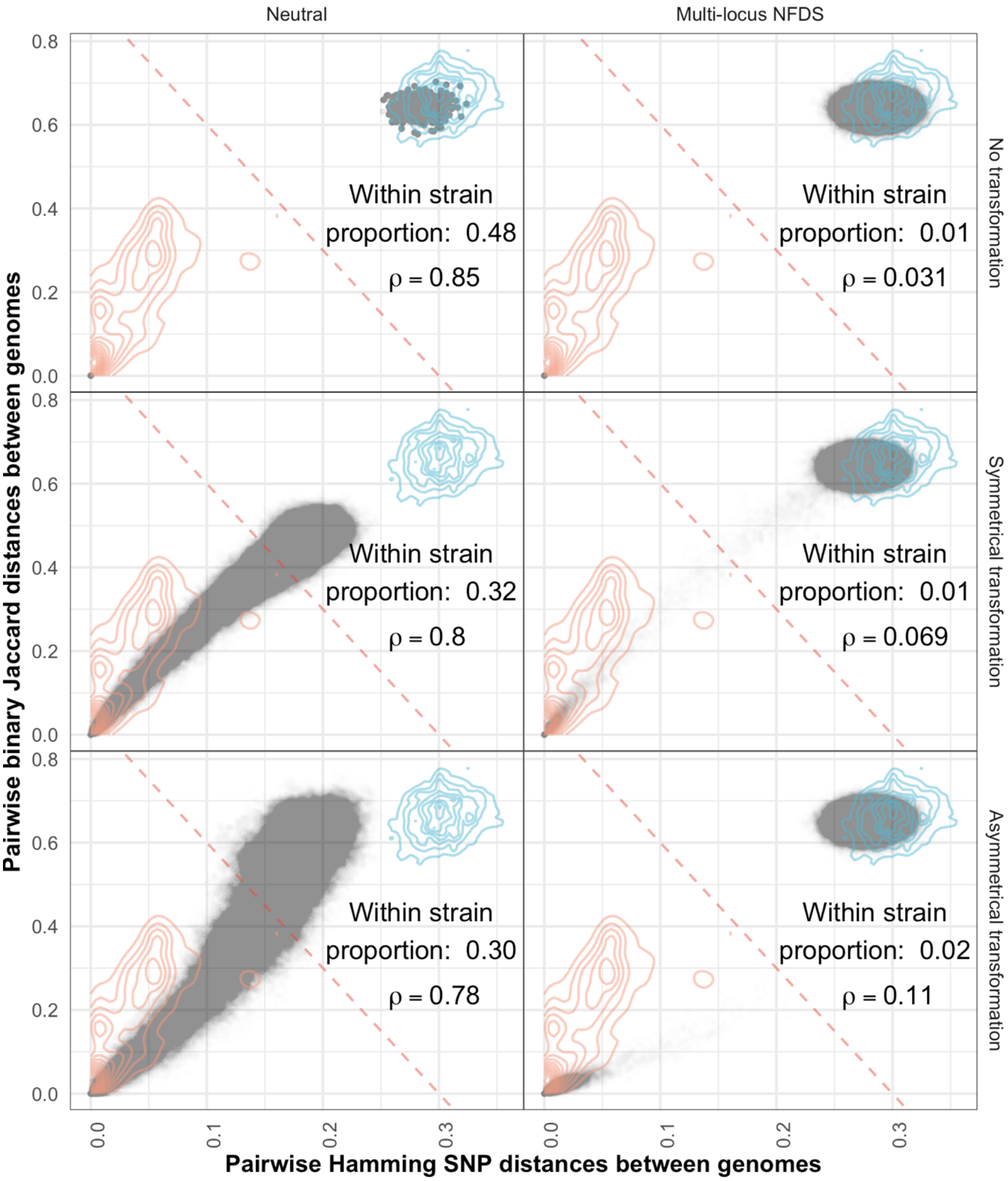

Figure S38: Scatterplots comparing the distributions of pairwise genetic distances between isolates at the final timepoint of simulations. Data are shown as in Fig. 4. The proportion of pairwise comparisons classified as within-strain (0.06 in the genomic data), based on the displayed threshold, and Spearman's correlation statistic (ρ; 0.38 in the genomic data) are shown in each panel; all correlation *p* values were $<10^{-10}$. These simulations were initiated with populations in which the alleles at each accessory locus, and SNP site, had been permuted across genotypes (Table 1).

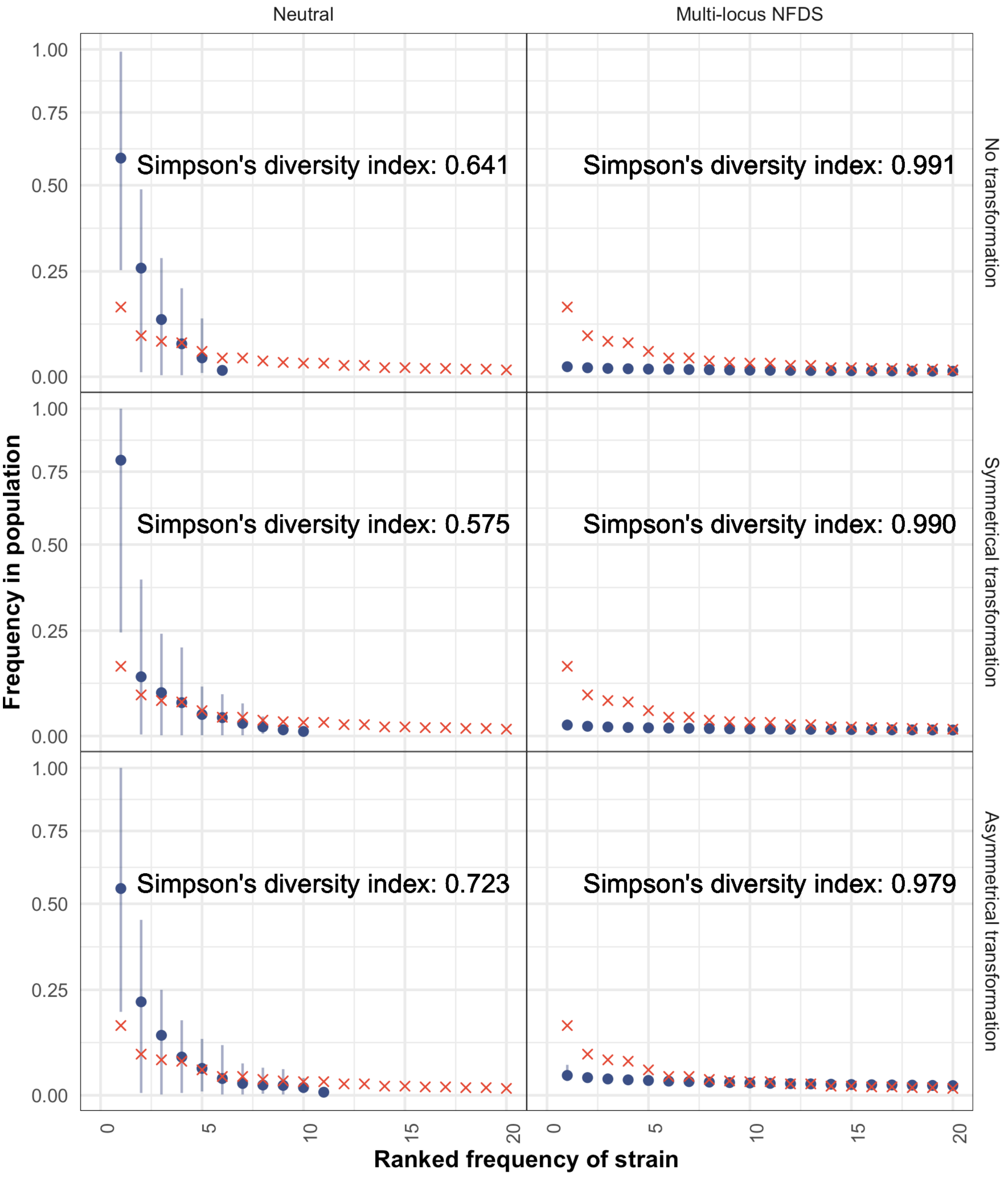

Figure S39: Scatterplots comparing the rank-frequency distributions of strains in the overall set of genomic data (red crosses) and those from samples of isolates from the final timepoint of 100 replicate simulations (blue points). Data are shown as in Fig. 5. Each panel shows the Simpson's diversity index (0.940 in the genomic data), calculated from the strain frequencies. These simulations were initiated with populations in which the alleles at each accessory locus, and SNP site, had been permuted across genotypes (Table 1).

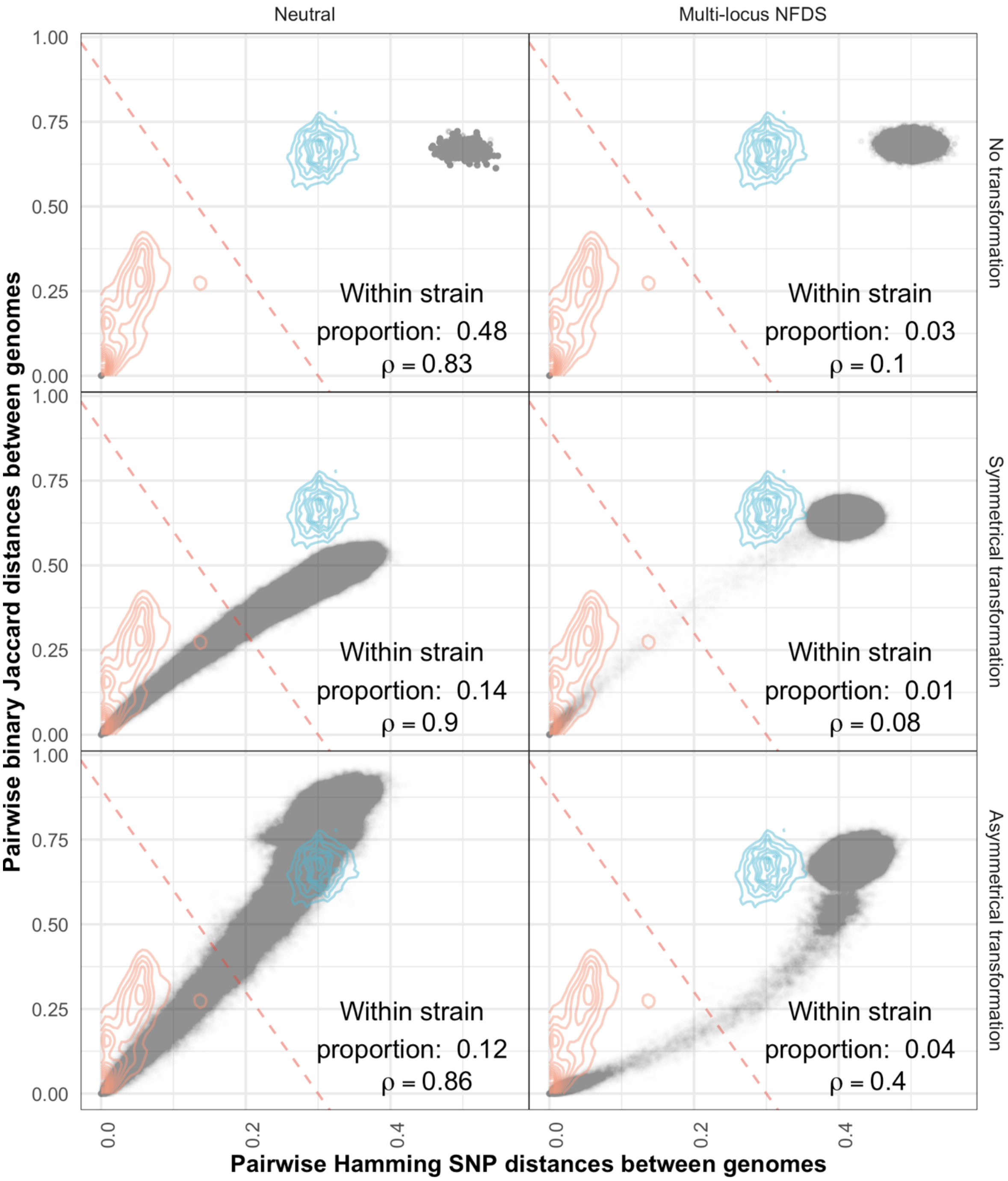

Figure S40: Scatterplots comparing the distributions of pairwise genetic distances between isolates at the final timepoint of simulations. Data are shown as in Fig. 4. The proportion of pairwise comparisons classified as within-strain (0.06 in the genomic data), based on the displayed threshold, and Spearman's correlation statistic (ρ; 0.38 in the genomic data) are shown in each panel; all correlation *p* values were $<10^{-10}$. These simulations were initiated with populations in which the alleles at each accessory locus, and SNP site, were randomly generated (Table 1).

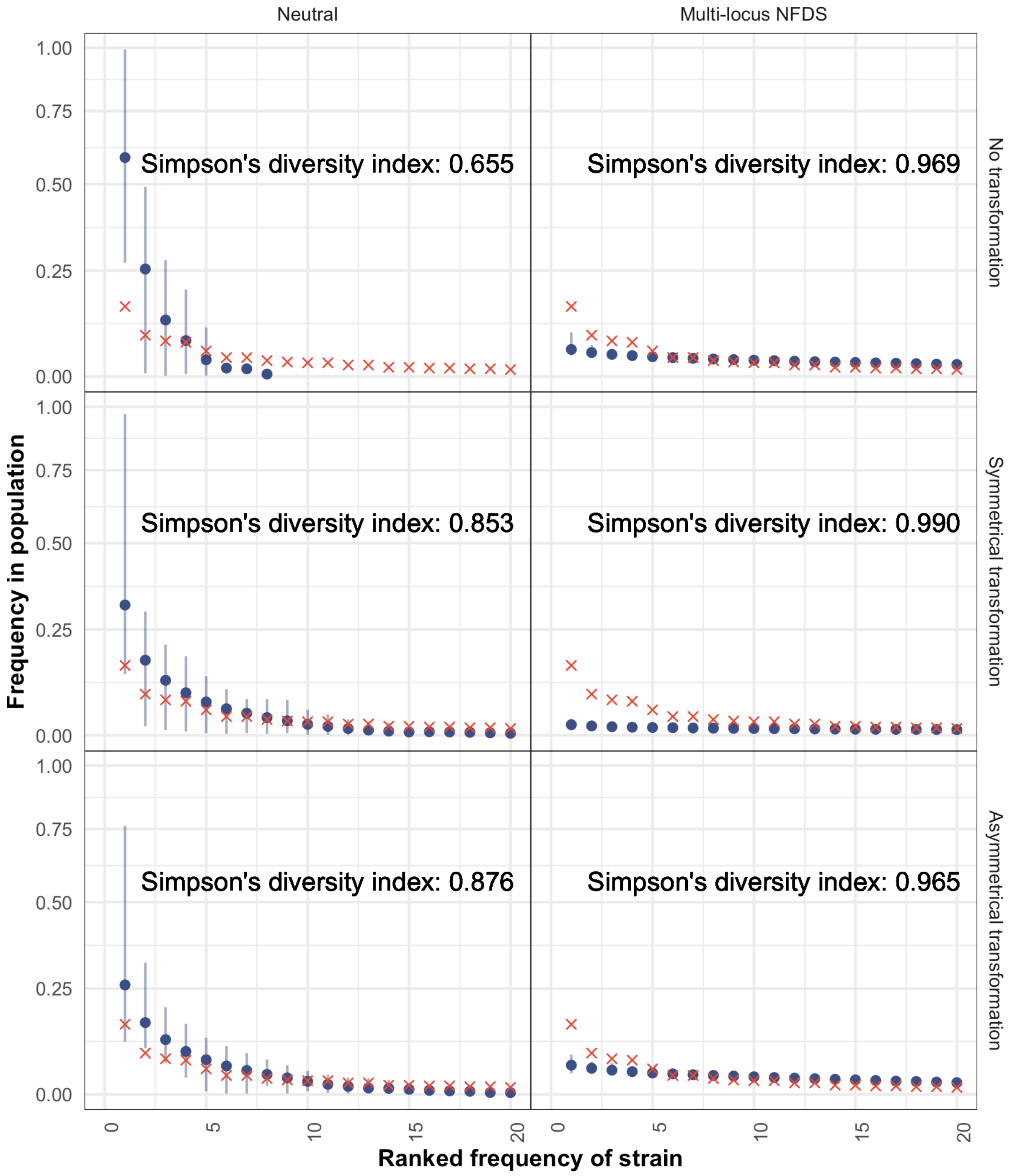

Figure S41: Scatterplots comparing the rank-frequency distributions of strains in the overall set of genomic data (red crosses) and those from samples of isolates from the final timepoint of 100 replicate simulations (blue points). Data are shown as in Fig. 5. Each panel shows the Simpson's diversity index (0.940 in the genomic data), calculated from the strain frequencies. These simulations were initiated with populations in which the alleles at each accessory locus, and SNP site, were randomly generated (Table 1).

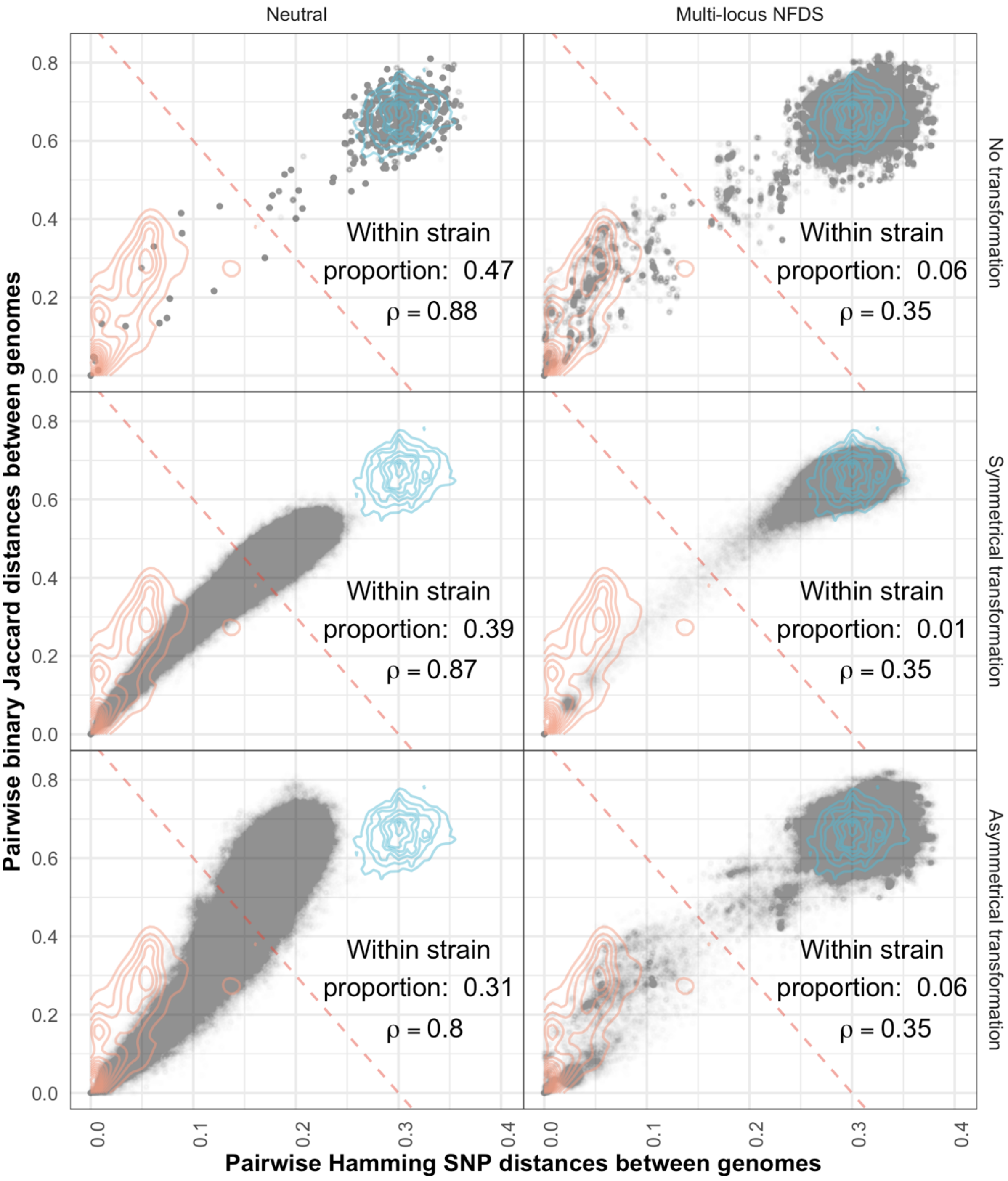

Figure S42: Scatterplots comparing the distributions of pairwise genetic distances between isolates at the final timepoint of simulations. Data are shown as in Fig. 4. The proportion of pairwise comparisons classified as within-strain (0.06 in the genomic data), based on the displayed threshold, and Spearman's correlation statistic (ρ; 0.38 in the genomic data) are shown in each panel; all correlation *p* values were $<10^{-10}$. These simulations featured saltational transformation (Table 1).

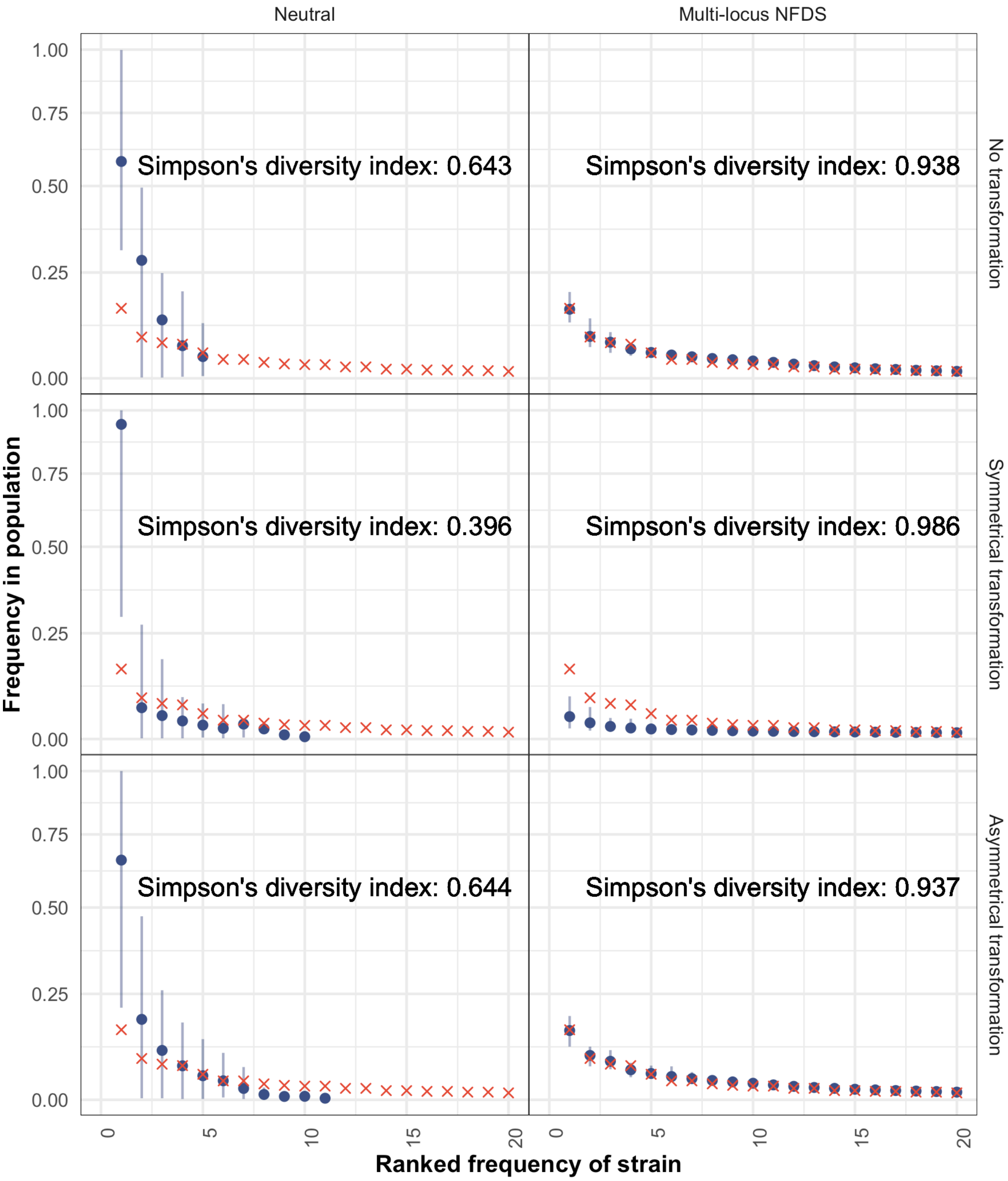

Figure S43: Scatterplots comparing the rank-frequency distributions of strains in the overall set of genomic data (red crosses) and those from samples of isolates from the final timepoint of 100 replicate simulations (blue points). Data are shown as in Fig. 5. Each panel shows the Simpson's diversity index (0.940 in the genomic data), calculated from the strain frequencies. These simulations featured saltational transformation (Table 1).

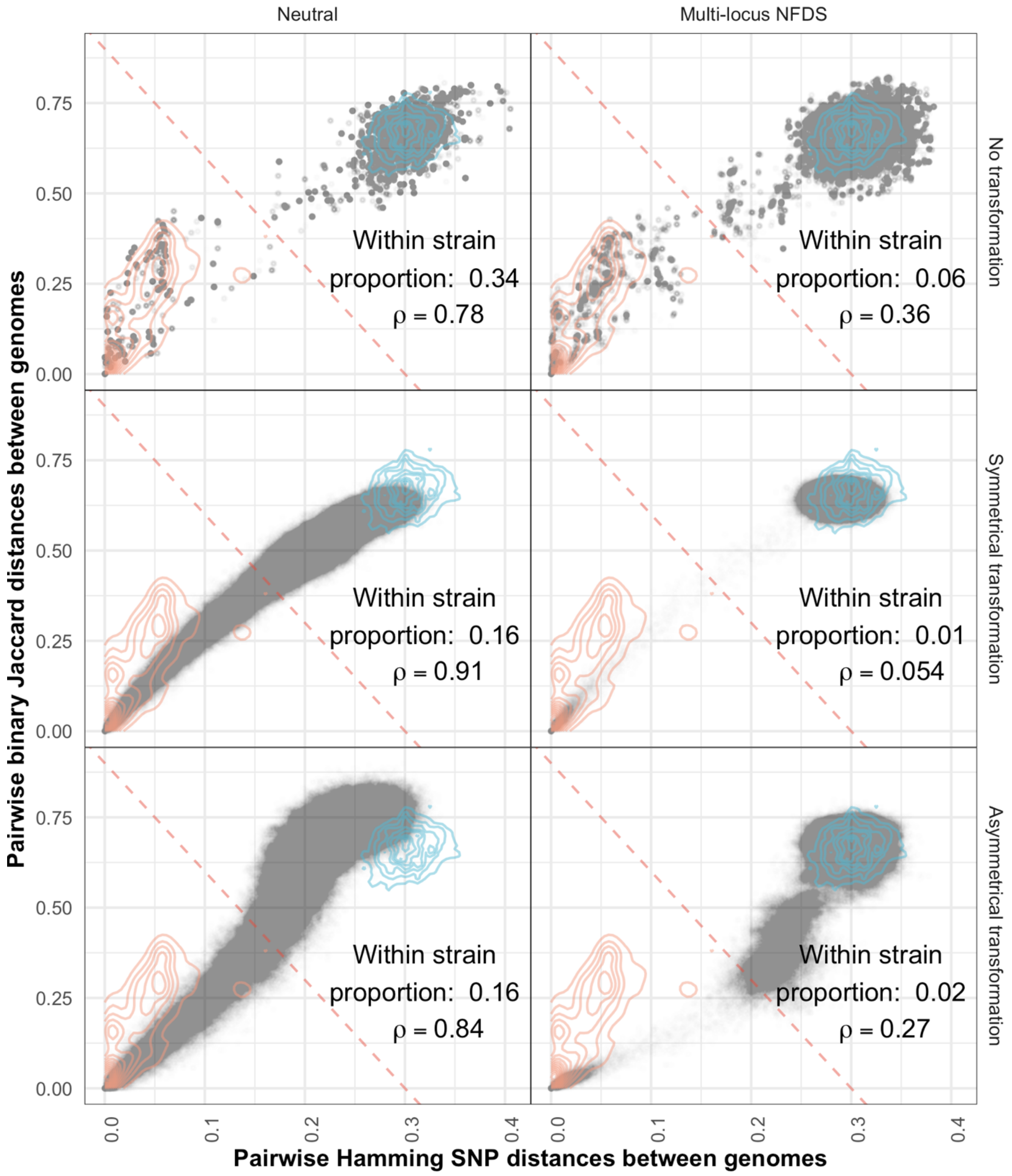

Figure S44: Scatterplots comparing the distributions of pairwise genetic distances between isolates at the final timepoint of simulations. Data are shown as in Fig. 4. The proportion of pairwise comparisons classified as within-strain (0.06 in the genomic data), based on the displayed threshold, and Spearman's correlation statistic (ρ; 0.38 in the genomic data) are shown in each panel; all correlation p values were $<10^{-10}$. These simulations featured inward migration (Table 1).

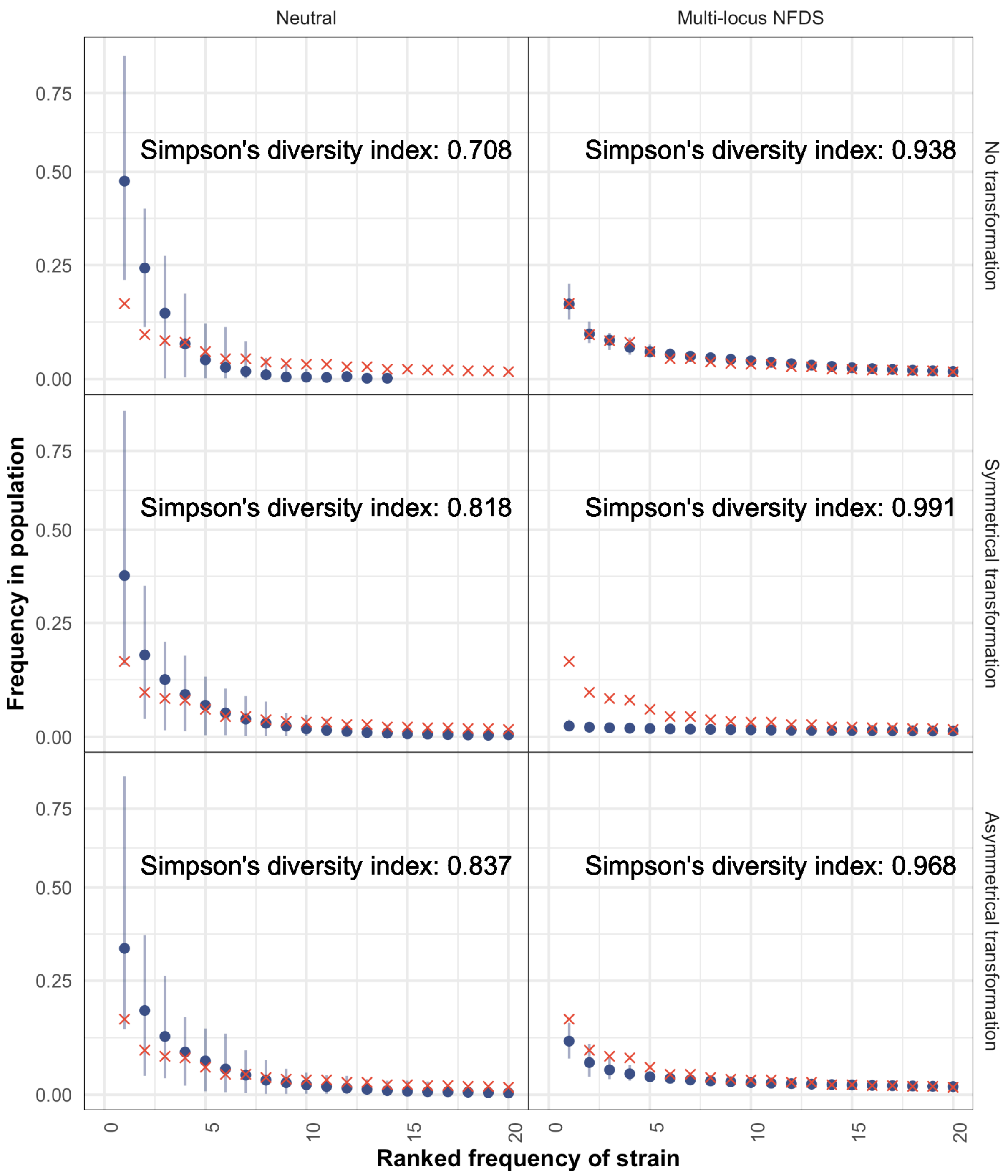

Figure S45: Scatterplots comparing the rank-frequency distributions of strains in the overall set of genomic data (red crosses), and those from samples of isolates from the final timepoint of 100 replicate simulations (blue points). Each panel shows the Simpson's diversity index (0.940 in the genomic data), calculated from the strain frequencies. Data are shown as in Fig. 5. These simulations featured inward migration (Table 1).

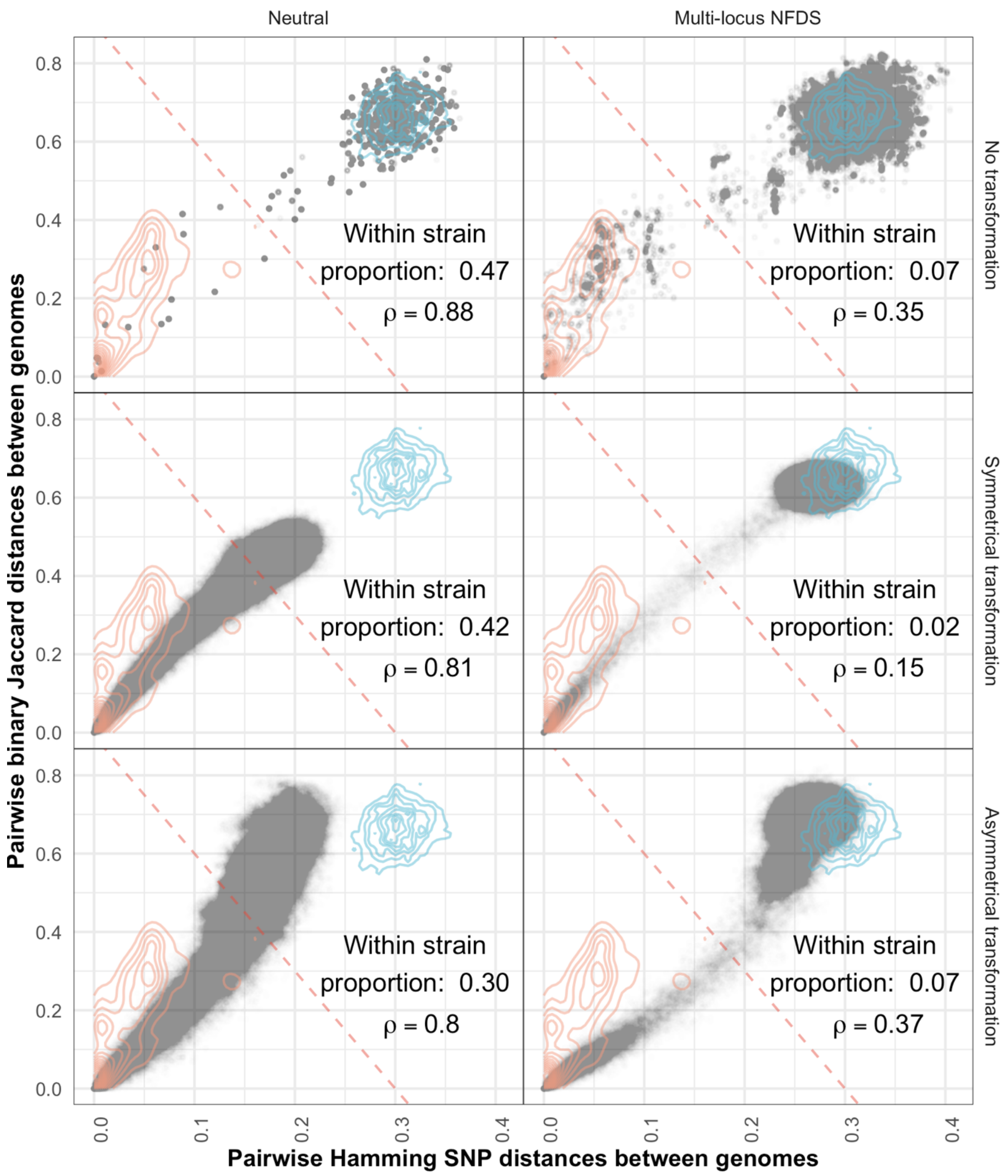

Figure S46: Scatterplots comparing the distributions of pairwise genetic distances between isolates at the final timepoint of simulations. Data are shown as in Fig. 4. The proportion of pairwise comparisons classified as within-strain (0.06 in the genomic data), based on the displayed threshold, and Spearman's correlation statistic (ρ; 0.38 in the genomic data) are shown in each panel; all correlation *p* values were $<10^{-10}$. These simulations featured weak multi-locus NFDS (Table 1).

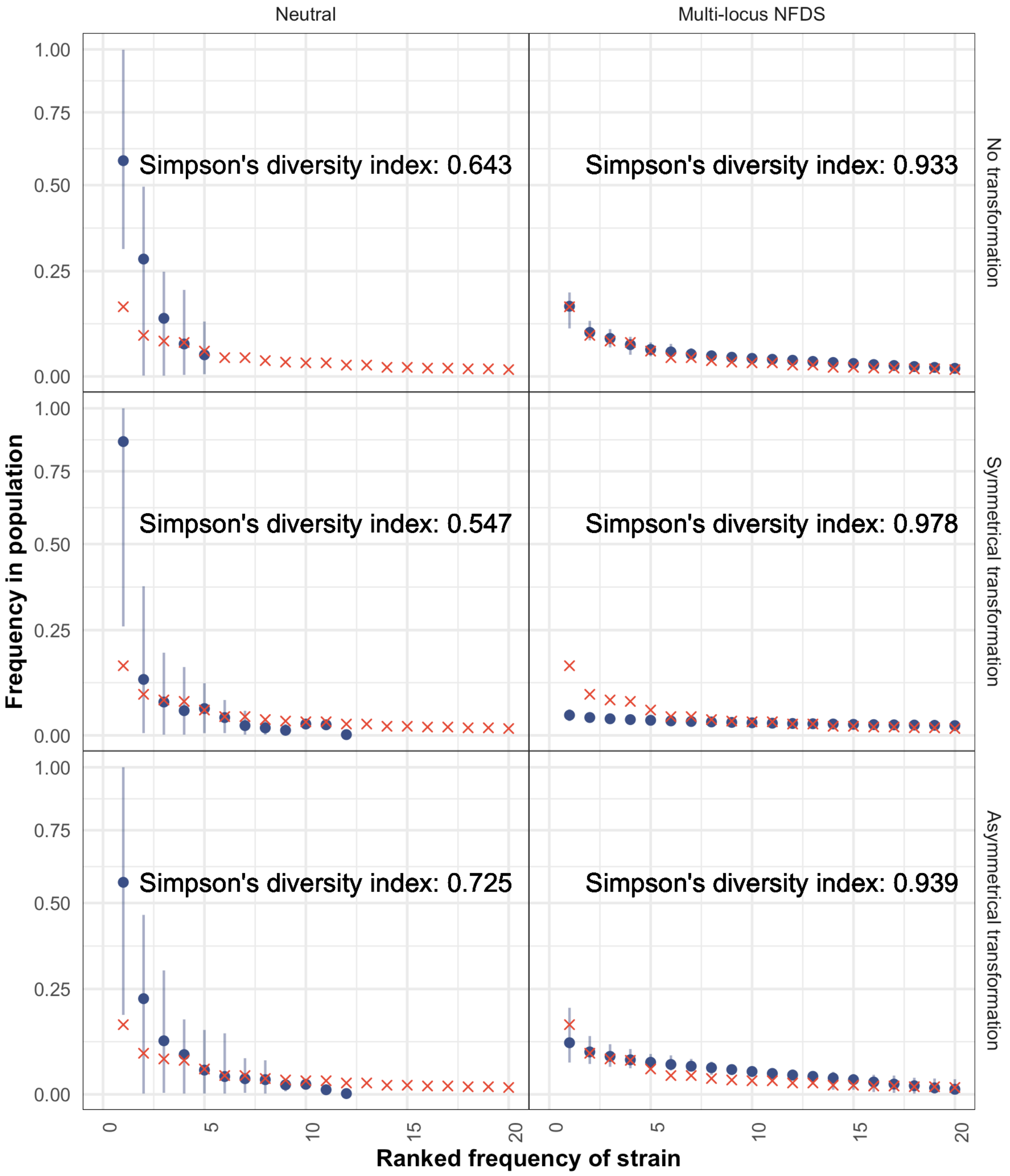

Figure S47: Scatterplots comparing the rank-frequency distributions of strains in the overall set of genomic data (red crosses) and those from samples of isolates from the final timepoint of 100 replicate simulations (blue points). Each panel shows the Simpson's diversity index (0.940 in the genomic data), calculated from the strain frequencies. Data are shown as in Fig. 5. These simulations featured weak multi-locus NFDS (Table 1).

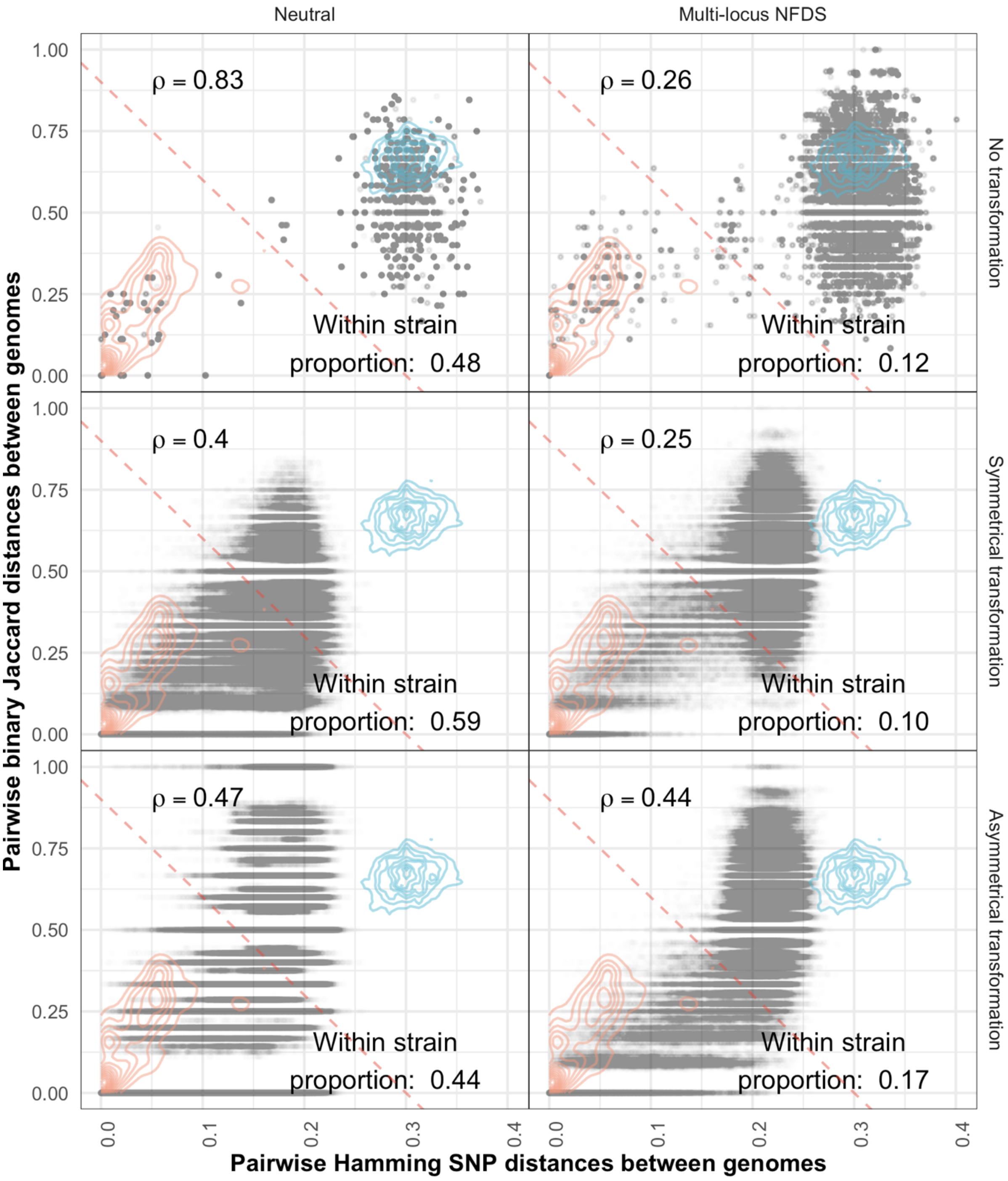

Figure S48: Scatterplots comparing the distributions of pairwise genetic distances between isolates at the final timepoint of simulations. Data are shown as in Fig. 4. The proportion of pairwise comparisons classified as within-strain (0.06 in the genomic data), based on the displayed threshold, and Spearman's correlation statistic (ρ; 0.38 in the genomic data) are shown in each panel; all correlation *p* values were $<10^{-10}$. These simulations included only a reduced subset of ten accessory loci being subject to multi-locus NFDS (Table 1).

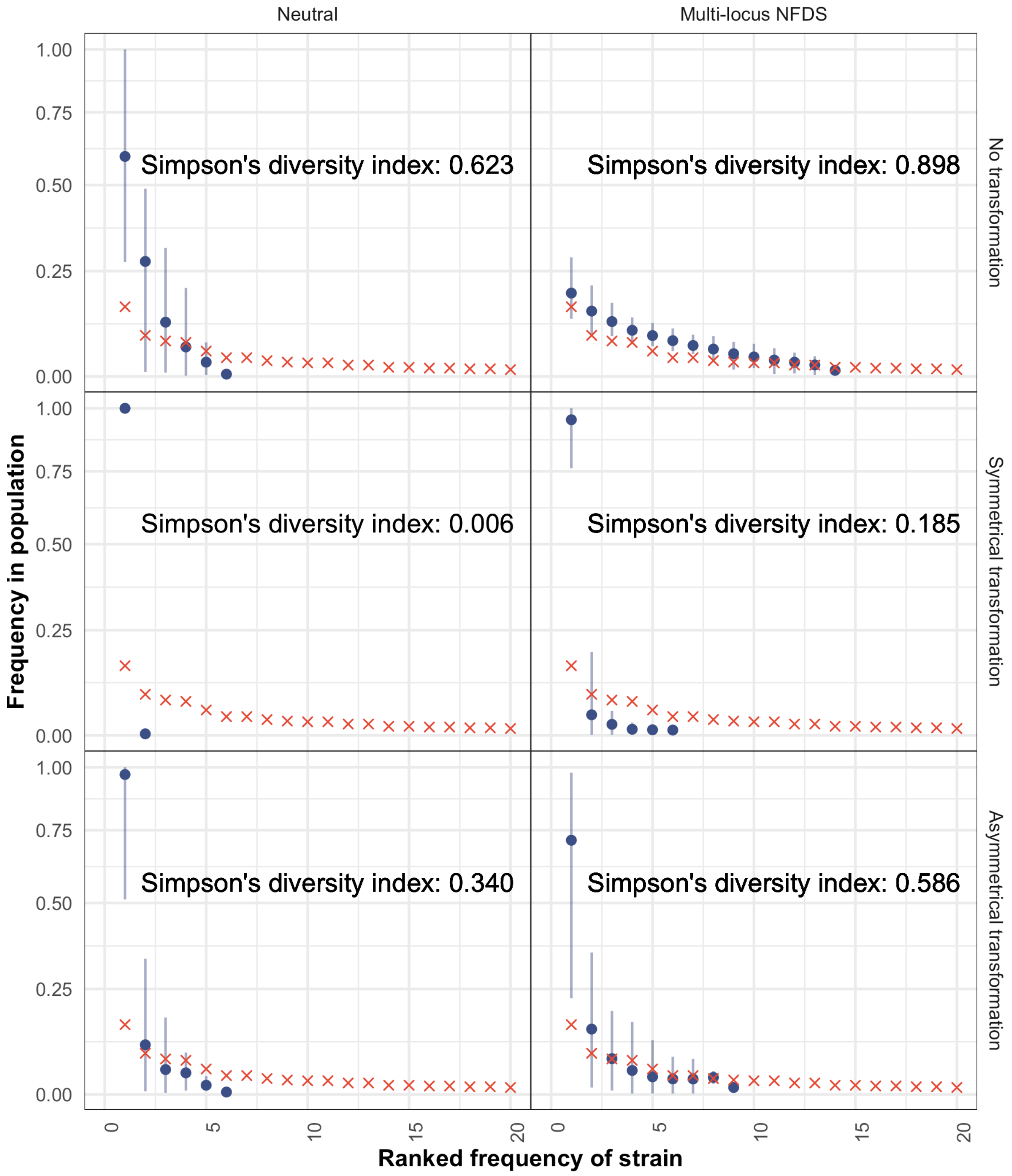

Figure S49: Scatterplots comparing the rank-frequency distributions of strains in the overall set of genomic data (red crosses) and those from samples of isolates from the final timepoint of 100 replicate simulations (blue points). Each panel shows the Simpson's diversity index (0.940 in the genomic data), calculated from the strain frequencies. Data are shown as in Fig. 5. These simulations included only a reduced subset of ten accessory loci being subject to multi-locus NFDS (Table 1).

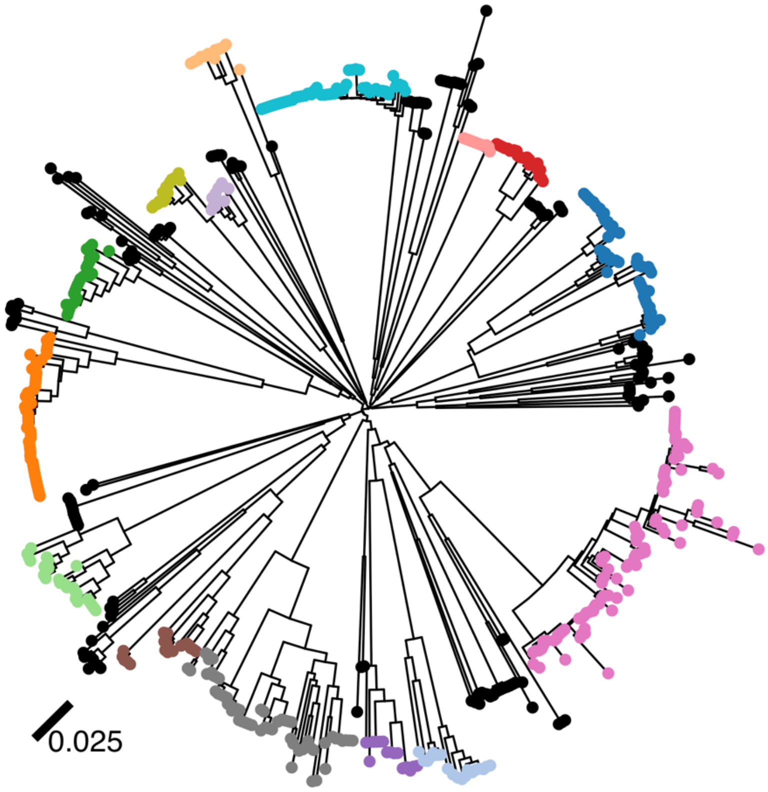

Figure S50: Neighbour-joining tree constructed from the intermediate-frequency core genome SNPs in the genomic data (*S* = 1090). Tips corresponding to isolates belonging to common strains, with more than 10 representatives in the population, are coloured according to this categorisation; other tips, corresponding to isolates of rarer strains, are coloured black.

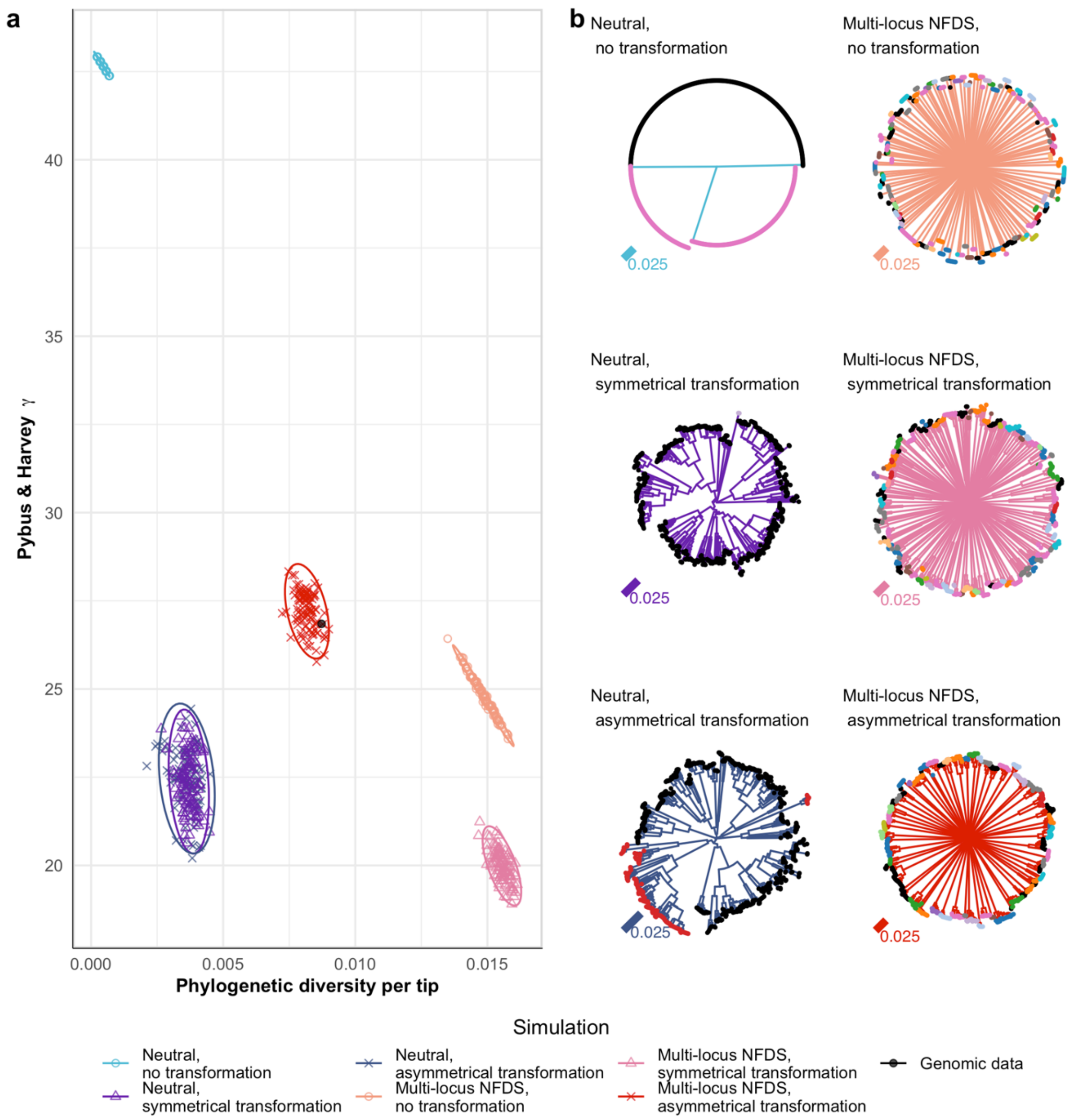

Figure S51: Comparison of trees generated from the genomic data and simulation outputs. Data are shown as in Fig. 6. **a** Scatterplot comparing the characteristics of the neighbour-joining trees **b** Representative trees from individual simulations from each parameter set. These simulations were initiated with populations in which the alleles at each accessory locus, and SNP site, had been permuted across genotypes (Table 1).

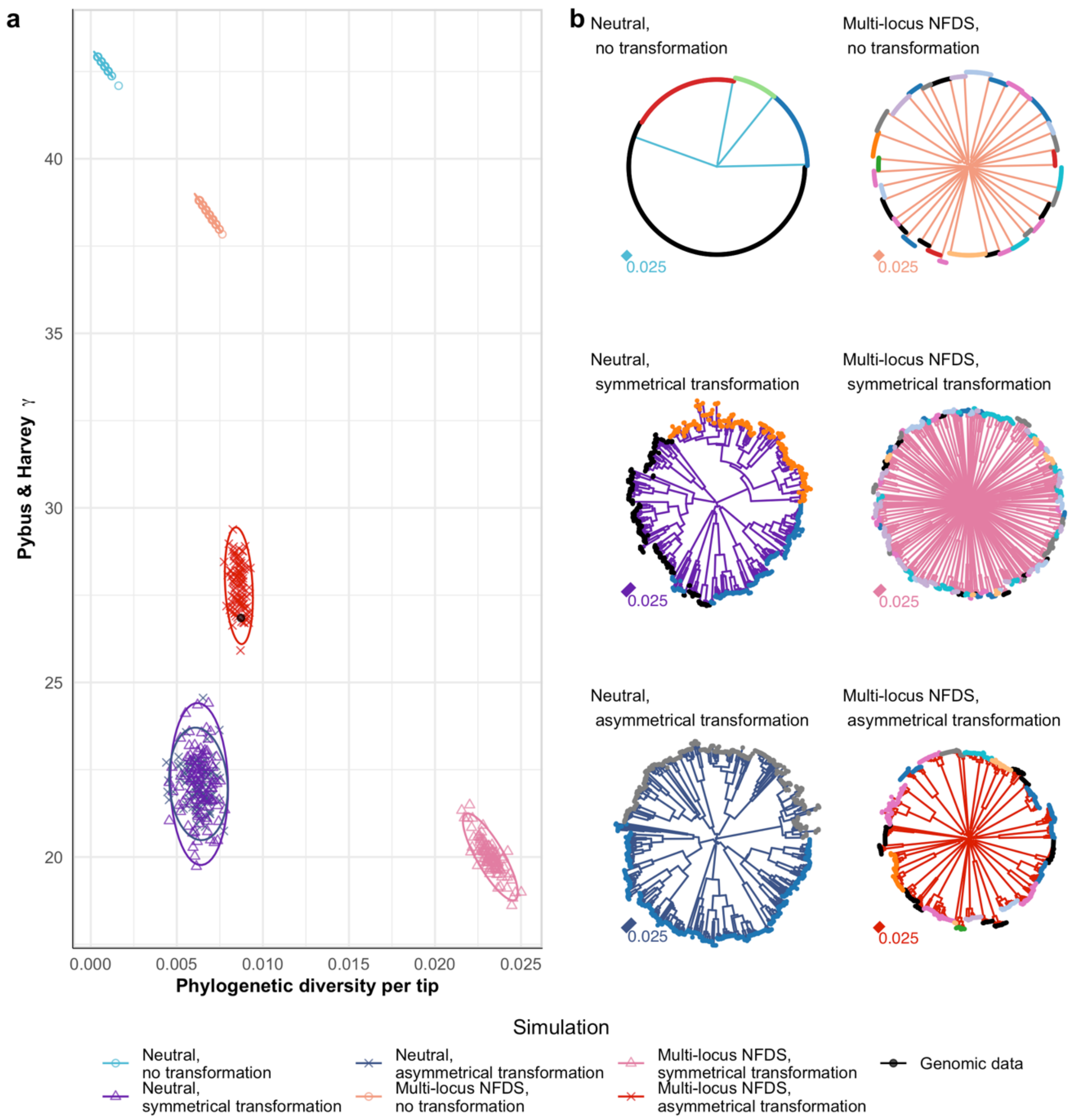

Figure S52: Comparison of trees generated from the genomic data and simulation outputs. Data are shown as in Fig. 6. **a** Scatterplot comparing the characteristics of the neighbour-joining trees **b** Representative trees from individual simulations from each parameter set. These simulations were initiated with populations in which the alleles at each accessory locus, and SNP site, were randomly generated (Table 1).

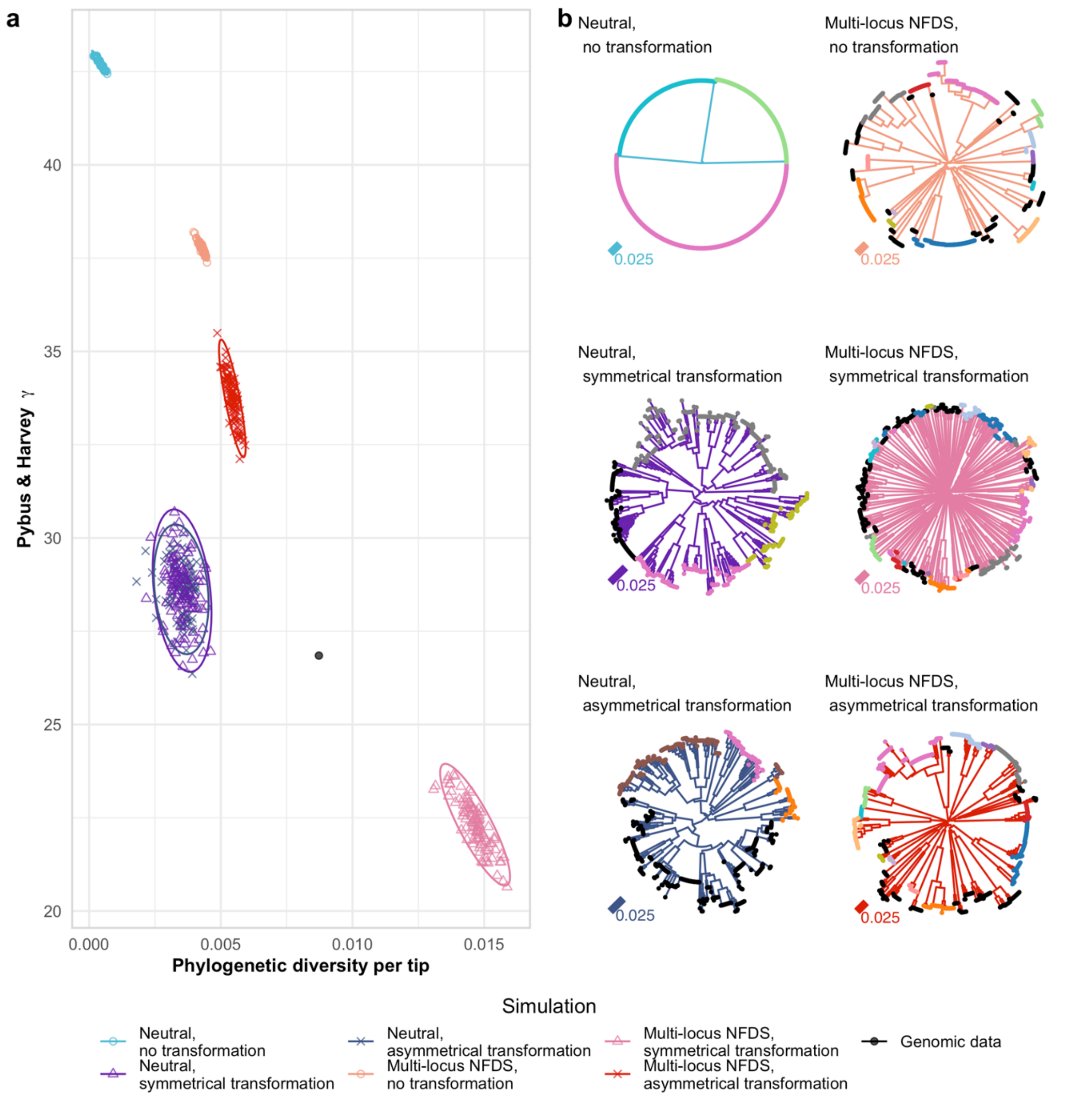

Figure S53: Comparison of trees generated from the genomic data and simulation outputs. Data are shown as in Fig. 6. **a** Scatterplot comparing the characteristics of the neighbour-joining trees **b** Representative trees from individual simulations from each parameter set. These simulations featured saltational transformation (Table 1).

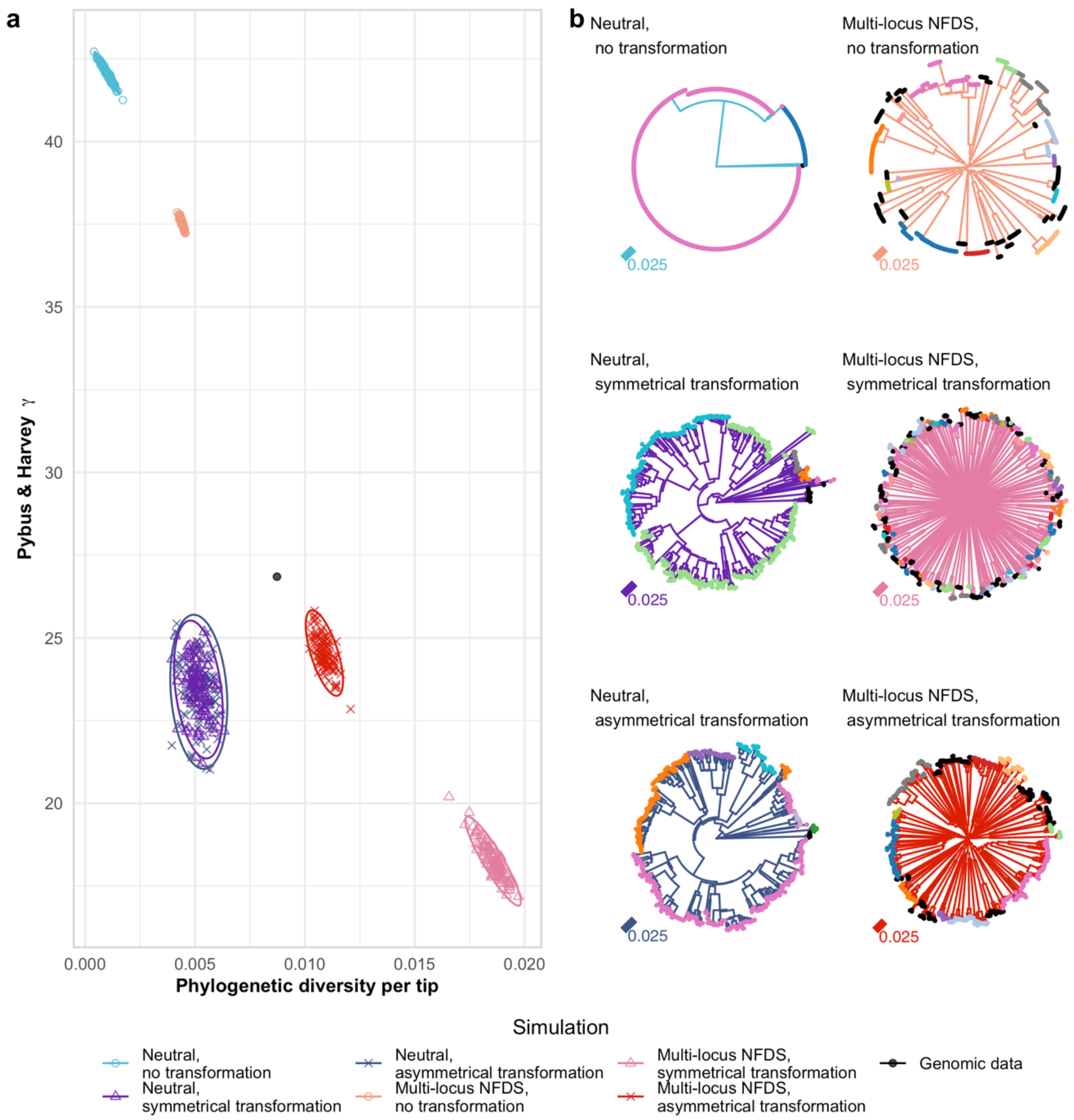

Figure S54: Comparison of trees generated from the genomic data and simulation outputs. Data are shown as in Fig. 6. **a** Scatterplot comparing the characteristics of the neighbour-joining trees **b** Representative trees from individual simulations from each parameter set. These simulations featured inward migration (Table 1).

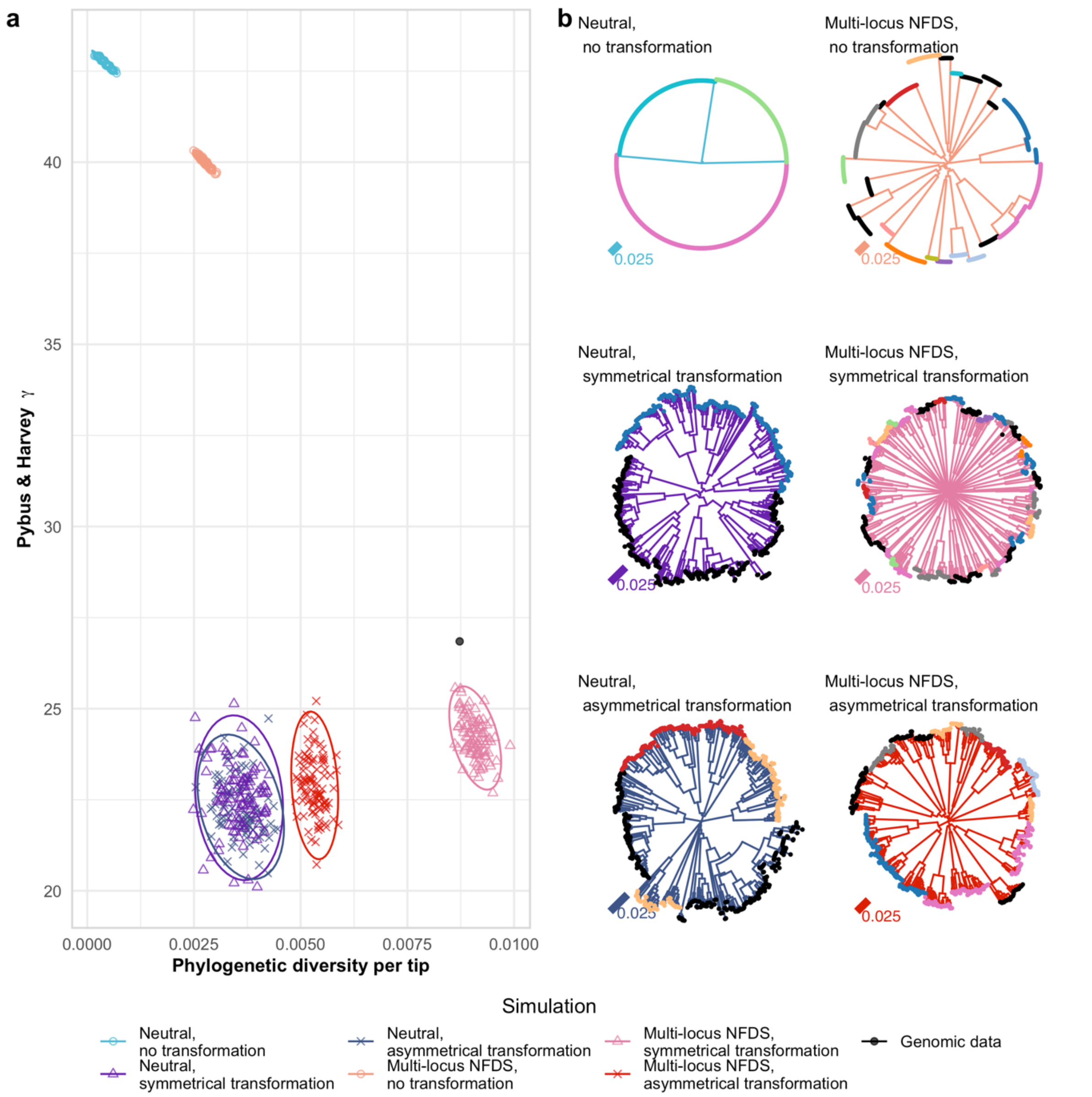

Figure S55: Comparison of trees generated from the genomic data and simulation outputs. Data are shown as in Fig. 6. **a** Scatterplot comparing the characteristics of the neighbour-joining trees **b** Representative trees from individual simulations from each parameter set. These simulations featured weak multi-locus NFDS (Table 1).

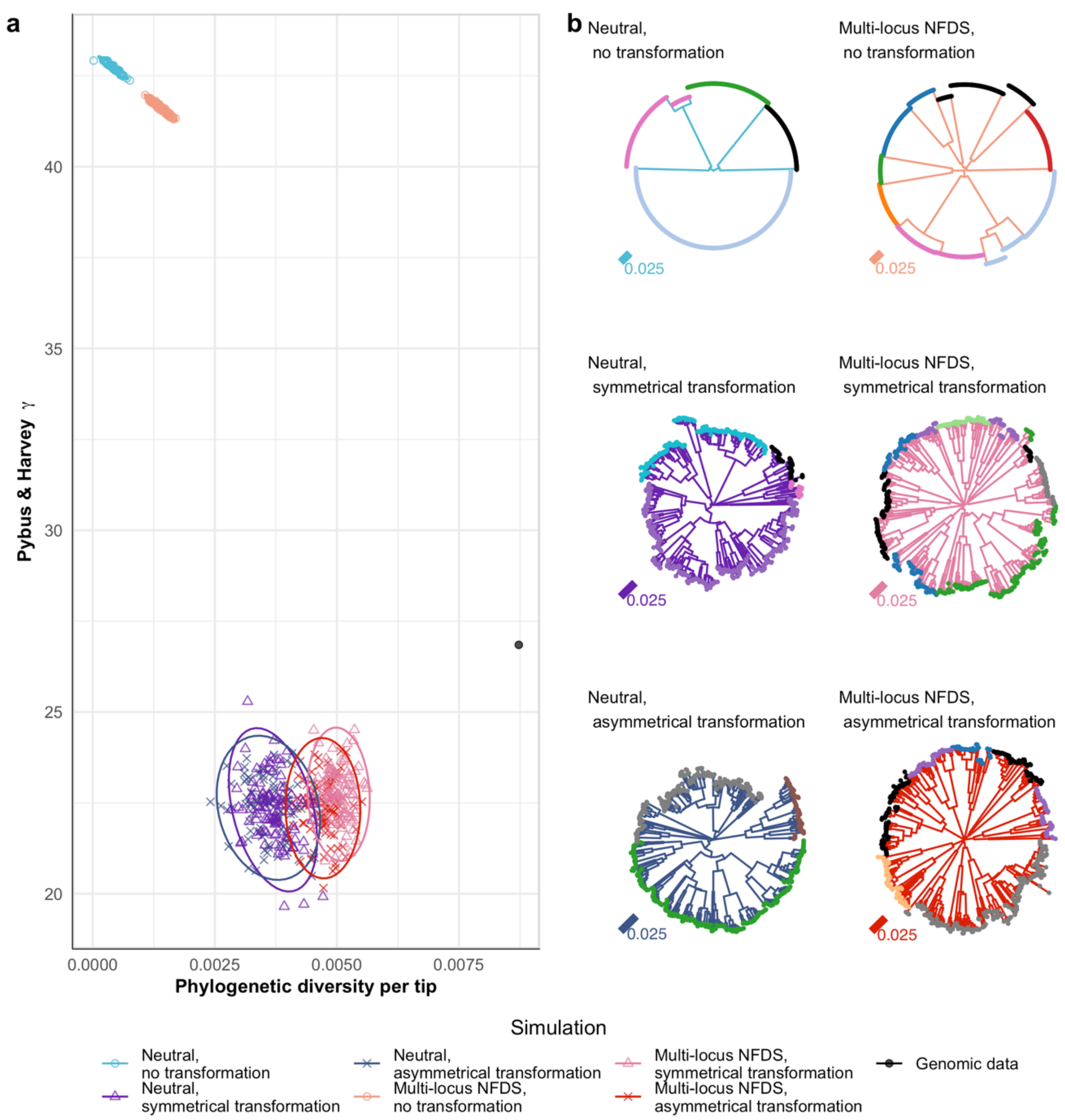

Figure S56: Comparison of trees generated from the genomic data and simulation outputs. Data are shown as in Fig. 6. **a** Scatterplot comparing the characteristics of the neighbour-joining trees **b** Representative trees from individual simulations from each parameter set. These simulations included only a reduced subset of ten accessory loci being subject to multi-locus NFDS (Table 1).

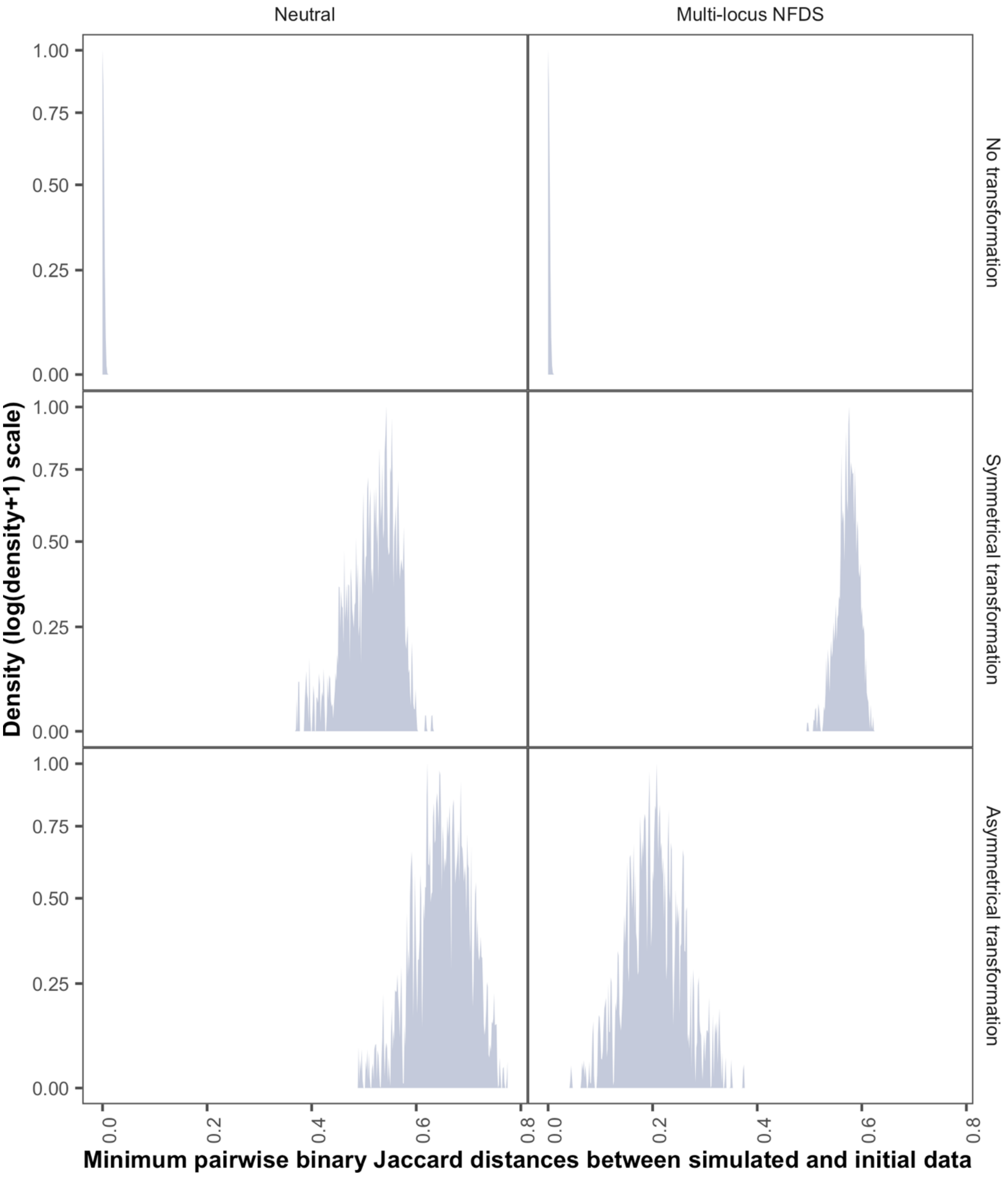

Figure S57: Density plot (using a bandwidth of 0.01) showing divergence of genotypes from the initial genomic data. The accessory locus content of each isolate sampled from the final timestep (*N* = 616) of each set of 100 replicate simulations was compared to the genomic data by calculating the pairwise binary Jaccard distances. These distributions show each simulated isolate's minimum distance to a genotype in the genomic data (overall *N* = 61,600 per panel).

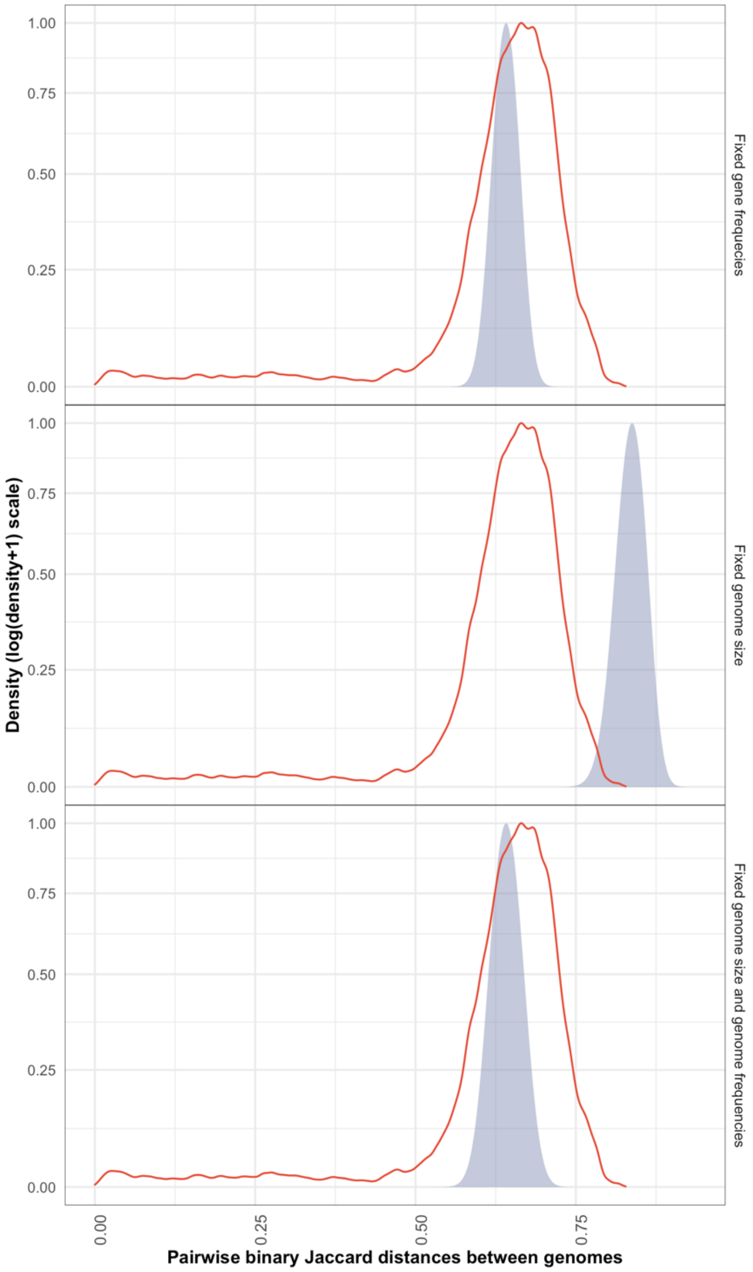

Figure S58: Density plot comparing the distributions of pairwise binary Jaccard distances, calculated from genotypes' accessory gene content, between isolates in the genome data (red line; N = 189,420) and from genotypes generated by permuting the $g_{i,l}$ matrix 100 times (blue filled area; N = 18,942,000 in each panel). The top plot shows the distances between genotypes generated by permuting the gene content while preserving genes at their equilibrium frequency. The middle plot shows the distances between genotypes generated by permuting the gene content while preserving the number of accessory genes in each genome. The bottom plot shows the distances between genotypes generated when permuting gene content under both constraints.

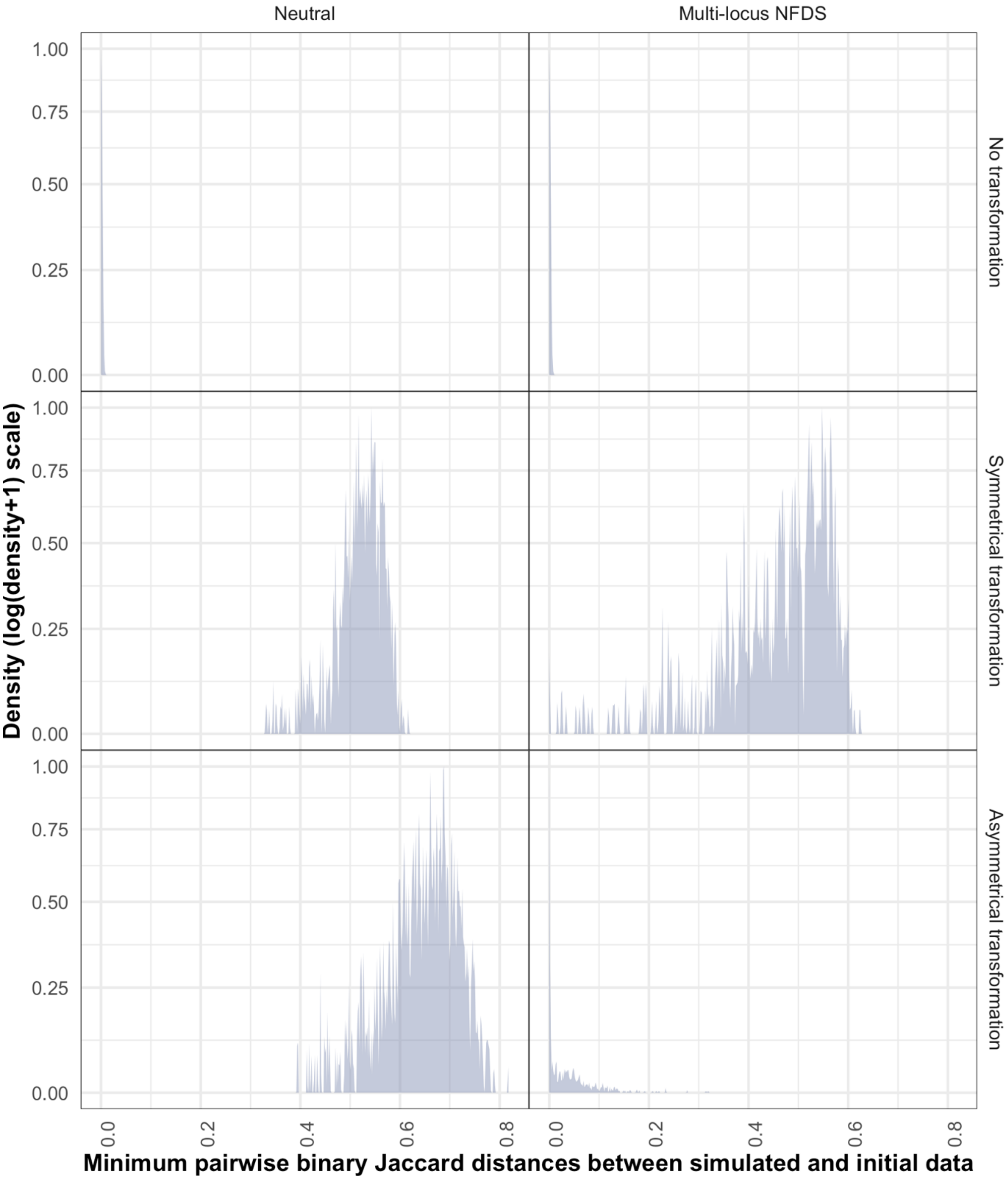

Figure S59: Density plot (using a bandwidth of 0.01) showing divergence of genotypes from the initial genomic data, as displayed in Fig. S45. These simulations featured saltational transformation (Table 1).
